# Mapping medically relevant RNA isoform diversity in the aged human frontal cortex with deep long-read RNA-seq

**DOI:** 10.1038/s41587-024-02245-9

**Published:** 2024-05-22

**Authors:** Bernardo Aguzzoli Heberle, J. Anthony Brandon, Madeline L. Page, Kayla A. Nations, Ketsile I. Dikobe, Brendan J. White, Lacey A. Gordon, Grant A. Fox, Mark E. Wadsworth, Patricia H. Doyle, Brittney A. Williams, Edward J. Fox, Anantharaman Shantaraman, Mina Ryten, Sara Goodwin, Elena Ghiban, Robert Wappel, Senem Mavruk-Eskipehlivan, Justin B. Miller, Nicholas T. Seyfried, Peter T. Nelson, John D. Fryer, Mark T. W. Ebbert

**Affiliations:** 1 Sanders-Brown Center on Aging, University of Kentucky, Lexington, KY USA; 2 Department of Neuroscience, College of Medicine, University of Kentucky, Lexington, KY USA; 3 Department of Pharmacology and Nutritional Sciences, College of Medicine, University of Kentucky, Lexington, KY USA; 4 Department of Biochemistry, Emory University School of Medicine, Atlanta, GA USA; 5 Department of Neurology, Emory University School of Medicine, Atlanta, GA USA; 6 UK Dementia Research Institute at The University of Cambridge, Cambridge, UK; 7 Department of Clinical Neurosciences, School of Clinical Medicine, University of Cambridge, Cambridge, UK; 8 Department of Genetics and Genomic Medicine, Great Ormond Street Institute of Child Health, University College London, London, UK; 9 Cold Spring Harbor Laboratory, Cold Spring Harbor, NY USA; 10 Division of Biomedical Informatics, Internal Medicine, College of Medicine, University of Kentucky, Lexington, KY USA; 11 Department of Pathology and Laboratory Medicine, University of Kentucky, Lexington, KY USA; 12 Microbiology, Immunology and Molecular Genetics, College of Medicine, University of Kentucky, Lexington, KY USA; 13 Department of Neuroscience, Mayo Clinic, Scottsdale, AZ USA

**Keywords:** RNA sequencing, RNA splicing, Neural ageing, Alzheimer's disease

## Abstract

Determining whether the RNA isoforms from medically relevant genes have distinct functions could facilitate direct targeting of RNA isoforms for disease treatment. Here, as a step toward this goal for neurological diseases, we sequenced 12 postmortem, aged human frontal cortices (6 Alzheimer disease cases and 6 controls; 50% female) using one Oxford Nanopore PromethION flow cell per sample. We identified 1,917 medically relevant genes expressing multiple isoforms in the frontal cortex where 1,018 had multiple isoforms with different protein-coding sequences. Of these 1,018 genes, 57 are implicated in brain-related diseases including major depression, schizophrenia, Parkinson’s disease and Alzheimer disease. Our study also uncovered 53 new RNA isoforms in medically relevant genes, including several where the new isoform was one of the most highly expressed for that gene. We also reported on five mitochondrially encoded, spliced RNA isoforms. We found 99 differentially expressed RNA isoforms between cases with Alzheimer disease and controls.

## Abstract (web summary)

The landscape of RNA isoforms in human cortex is revealed by deep long-read RNA sequencing.

## Main

Human protein-coding genes average more than eight RNA isoforms, resulting in almost four distinct protein-coding sequences^1,2^. As a result of practical limitations in standard short-read sequencing technologies, researchers have historically been forced to collapse all isoforms into a single gene expression measurement, a major oversimplification of the underlying biology. Many unique isoforms from a single gene body appear to have unique interactomes at the protein level^3^. Distinct functions for individual isoforms from a single gene body have already been demonstrated for a handful of genes^4–6^. Notably, isoforms can play entirely different, or even opposite, roles within a given cell; a classic example includes two well-studied *BCL-X* (*BCL2L1*) transcripts with opposite functions, where *BCL-X*_*L*_ is anti-apoptotic and *BCL-X*_*S*_ is pro-apoptotic^6^. Changes in the expression ratio between the *BCL-X* isoforms are implicated in cancer and are being studied as therapeutic targets^7^, demonstrating the importance of understanding individual RNA isoform function rather than treating them as a ‘single’ gene.

Knowing which tissues and cell types express each isoform is an important first step in understanding isoform function. The limitations of using short-read sequencing for studying differential RNA isoform expression/usage^8,9^ include relying on heuristics to assemble and quantify isoforms^10–12^. As a result of these limitations, detailed analysis of individual isoforms has been limited to highly studied genes. In principle, long reads can sequence the entire isoforms directly^12^. However, the imperfections of long-read data^13^ still require some heuristics to estimate the expression of each isoform^13,14^. Recent long-read RNA sequencing (RNA-seq) studies used targeted approaches to uncover aberrant splicing events in sporadic Alzheimer disease (AD)^15^, dystrophinopathies^16^ and cancers^17,18^. Two other studies demonstrated that long-read sequencing can discover new RNA isoforms across several human tissues, including the brain^19,20^. Although both studies revealed important biology, including reporting new RNA isoforms, they had limited sequencing coverage (averaging <6 million aligned reads per sample). Read depth is essential to accurately quantify individual RNA isoforms, given that a total of >250,000 annotated RNA isoforms have been reported, as of July 2023 (ref. ^2^). In addition, neither of the studies focused on the medical relevance of using long-read RNA-seq. Although long-read sequencing does not resolve all challenges related to isoform sequencing (for example, those related to RNA degradation), our goal is to demonstrate the utility and importance of using long-read sequencing for both academic research and clinical diagnostics in the context of RNA isoforms (for example, reporting newly discovered RNA isoforms in medically relevant genes and variant interpretation in genes expressing multiple RNA isoforms).

In the present study, we demonstrate that RNA isoform quantification through deep long-read sequencing can be a step toward understanding the function of individual RNA isoforms, and provide insights into how they may impact human health and disease. Specifically, in addition to discovering new (that is, unannotated) RNA isoforms in known medically relevant genes, we also discovered new spliced mitochondria-encoded RNA isoforms and entirely new gene bodies in nuclear DNA and demonstrated the complexity of RNA isoform diversity for medically relevant genes within a single tissue (human frontal cortex from patients with AD and controls). Last, we showed the potential of differential RNA isoform expression analysis to reveal disease-relevant transcriptomic signatures unavailable at the gene level (that is, when collapsing all isoforms into a single expression measurement). Summary data from the present study are readily explorable through a public web application to visualize individual RNA isoform expression in aged human frontal cortex tissue (https://ebbertlab.com/brain_rna_isoform_seq.html).

## Results

### Methodological and results overview

**Fig. 1:**
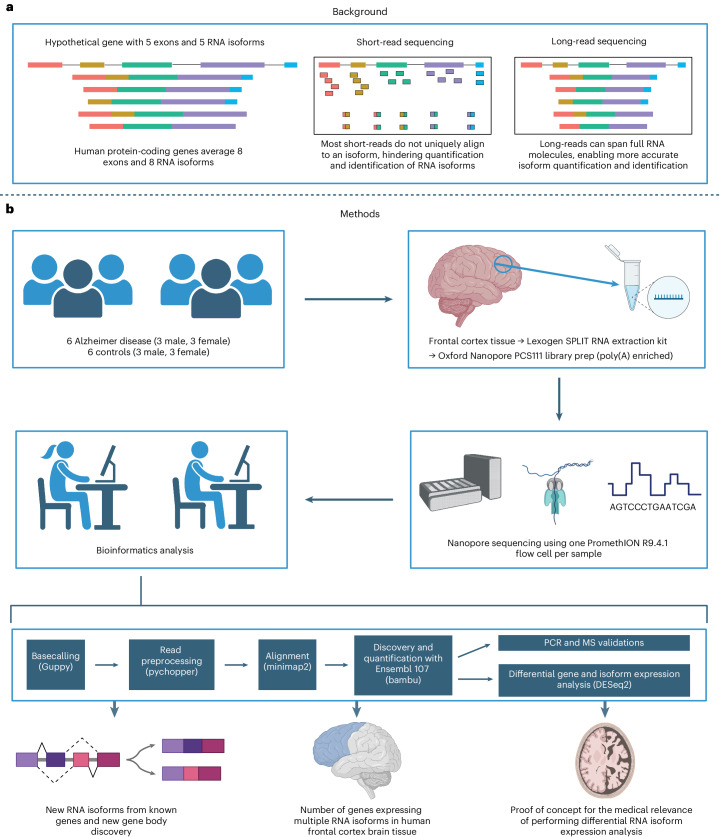
Study design and rationale. **a**, Background explaining the improvements long-read sequencing brings to the study of RNA isoforms. **b**, Details for experimental design, methods and a summary of the topics explored in this article. MS, mass spectrometry. Created with BioRender.com.

**Extended Data Fig. 1:**
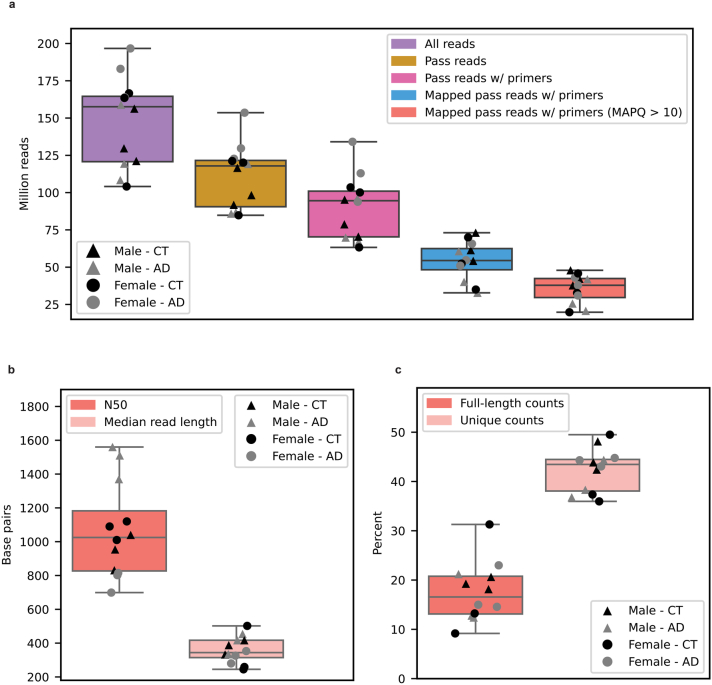
Basic sequencing metrics. AD = Alzheimer’s disease cases, CT = Cognitively unimpaired aged controls. **a**, Number of reads per sample after each step of the analysis. All downstream analysis were done with Mapped pass reads with both primers and MAPQ > 10. **b**, N50 and median read length for Mapped pass reads with both primers and MAPQ > 10. **c**, Percentage of reads that are full-length or unique as determined by bambu. Full-length counts = reads containing all exon-exon boundaries (that is, intron chain) from its respective transcript. Unique counts = reads that were assigned to a single transcript. All boxplots from this panel come from n = 12 biologically independent samples. Male AD n = 3, Female AD n = 3, Male CT n = 3, Female CT n = 3. All boxplots in this panel follow this format: center line, median; box limits, upper and lower quartiles; whiskers, 1.5x interquartile range.

Traditional RNA-seq studies relied on short-read sequencing approaches that excel at quantifying gene-level expression, but cannot accurately assemble and quantify a large proportion of RNA isoforms^11,21^ (Fig. 1a). Thus, we sequenced 12 postmortem, aged, dorsolateral prefrontal cortex (Brodmann area 9/46) brain samples individually from six patients with AD and six cognitively unimpaired controls (50% female; Fig. 1b). All samples had postmortem intervals <5 h and an RNA integrity score (RIN) ≥ 9.0; demographics, summary sequencing statistics and read length distributions are shown in Supplementary Table 1 and Supplementary Figs. 1–4. Poly(A)-enriched complementary DNA from each sample was sequenced using one PromethION flow cell. Sequencing yielded a median of 35.5 million aligned reads per sample after excluding reads lacking the primer on either end and those with a mapping quality <10 (Extended Data Fig. 1a). By excluding all reads missing primers, reads included in the present study should closely represent the RNA as it was at extraction.

We performed RNA isoform quantification and discovery (including new gene bodies) using bambu^14^ (Fig. 1b)—a tool with emphasis on reducing false-positive RNA isoform discovery compared with other commonly used tools^14^. Bambu was highlighted as a top performer in a recent benchmark study^13^. However, as a tradeoff for higher precision, bambu is unable to discover new RNA isoforms that only differ from annotated RNA isoforms at the transcription start and/or end site (for example, shortened 5′-UTR). When it comes to quantification, the increasing complexity of annotations can impact quantification owing to non-unique reads being split between multiple transcripts. For example, if a read maps equally well to two RNA isoforms, each isoform will receive credit for 0.5 reads.

**Extended Data Fig. 2:**
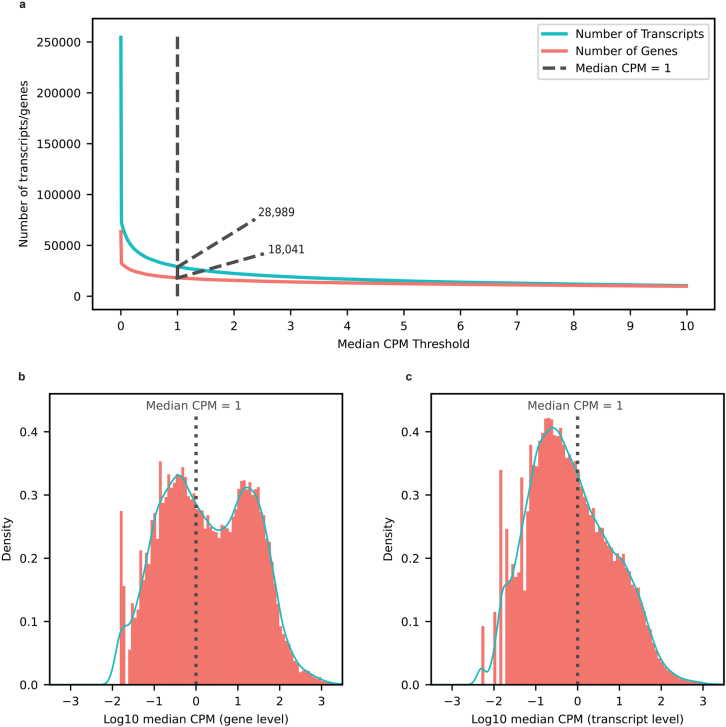
Expression distribution and diversity for genes and transcripts. **a**, Number of genes and transcripts represented across median CPM threshold. Cutoff shown as the dotted line set at median CPM = 1. **b**, Distribution of log_10_ median CPM values for gene bodies, dotted line shows cutoff point of median CPM = 1. **c**, Distribution of log_10_ median CPM values for gene bodies, dotted line shows cutoff point of median CPM = 1.

**Extended Data Fig. 3:**
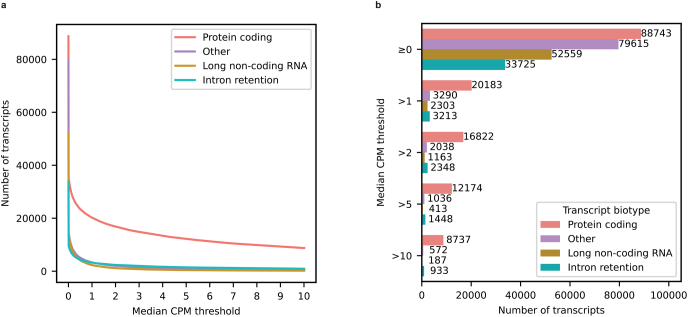
Expression of different transcript biotypes on aged human frontal cortex tissue using long-read RNAseq data. **a**, Lineplot showing the number of transcripts from different biotypes expressed above different median CPM threshold in long-read RNAseq data from aged human dorsolateral prefrontal cortex postmortem tissue. **b**, Barplot showing the number of transcripts from different biotypes expressed at or above different median CPM threshold in long-read RNAseq data from aged human dorsolateral prefrontal cortex postmortem tissue.

For our 12 samples, bambu reported an average of 42.4% reads uniquely assigned to an RNA isoform and 17.5% reads spanning a full-length RNA isoform (Extended Data Fig. 1c). We considered an isoform to be expressed above noise levels only if its median counts per million (CPM) was >1 (that is, at least half of the samples had a CPM > 1); this threshold is dependent on overall depth, because lower depths will require a higher, more stringent CPM threshold. Using this threshold, we observed 28,989 expressed RNA isoforms from 18,041 gene bodies in our samples (Extended Data Fig. 2a–c). Of the RNA isoforms expressed with median CPM > 1, exactly 20,183 were classified as protein coding, 2,303 as long noncoding RNAs, 3,213 as having a retained intron and the remaining 3,290 were scattered across other biotypes—including new transcripts (Extended Data Fig. 3).

We used publicly available mass spectrometry (MS) data from aged, human dorsolateral prefrontal cortex tissue (Brodmann area 9)^22,23^ and human cell lines^24^ to validate new RNA isoforms at the protein level, resulting in a small number of successful validations. We also leveraged existing short-read RNA-seq data from the Religious Orders Study Memory and Aging Project (ROSMAP)^25,26^ and long-read RNA-seq data from Glinos et al.^19^ to validate our newly discovered RNA isoforms and gene bodies.

### Discovery of new RNA isoforms from known gene bodies

Our first goal was to identify and quantify new RNA isoforms expressed in human frontal cortex. In total, bambu discovered 1,534 new transcripts from known (that is, annotated) nuclear gene bodies. Of these 1,534 new RNA isoforms, exactly 1,106 had a median CPM ≤ 1. Although we expect that many of these new RNA isoforms with a median CPM ≤ 1 are legitimate, we consider them low-confidence discoveries and exclude them throughout the remainder of our analyses, except where explicitly noted.

**Fig. 2:**
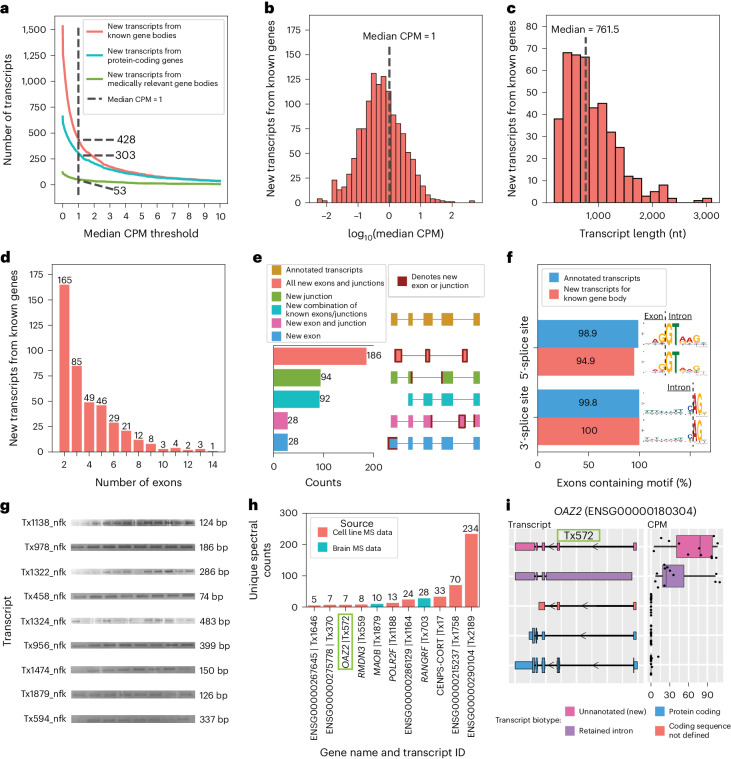
New high-confidence RNA isoforms from known gene bodies expressed in human frontal cortex tissue. **a**–**f**, New transcripts from annotated gene bodies. **a**, Number of newly discovered transcripts across the median CPM threshold. The cutoff is shown as the dashed line set at median CPM = 1. **b**, Distribution of log_10_(median CPM values) for newly discovered transcripts. The dashed line shows the cutoff point of median CPM = 1. **c**–**f**, Data only from transcripts above this expression cutoff. **c**, Histogram showing distribution of transcript length for new transcripts from annotated gene bodies. **d**, Bar plot showing the distribution of the number of exons for newly discovered transcript. **e**, Bar plot showing the kinds of events that gave rise to new transcripts (in part created with BioRender.com). **f**, Bar plot showing the prevalence of canonical splice site motifs for annotated exons from transcripts with median CPM > 1 versus new exons from new transcripts. **g**, Gel electrophoresis validation using PCR amplification for a subset of new RNA isoforms from known genes. This is an aggregate figure showing bands for several different gels. Each gel electrophoresis PCR experiment was independently performed once with similar results. Individual gel figures are available in Supplementary Figs. 5–26. **h**, Protein level validation using publicly available MS proteomics data. The *y* axis shows the number of spectral counts from uniquely matching peptides (unique spectral counts). New transcripts from known gene bodies were considered validated at the protein level when reaching more than five unique spectral counts. **i**, RNA isoform structure and expression for *OAZ2* transcripts (cellular growth/proliferation). The new isoform Tx572 was most expressed and validated at the protein level (highlighted with the green box). Boxplot format: median (center line), quartiles (box limits), 1.5 × interquartile range (IQR) (whiskers) (*n* = 12 biologically independent samples).

After excluding all isoforms with a median CPM ≤ 1,428, isoforms remained that we consider high confidence (Fig. 2a,b), where 303 were from protein-coding genes (Fig. 2a). We report substantially fewer new isoforms compared with Glinos et al.^19^ (~70,000) and Leung et al.^20^ (~12,000) because of: (1) differences in the reference database; (2) the discovery tool employed^13,27^ (that is, bambu^14^ versus FLAIR^28^ versus Cupcake^29^); and (3) sequencing depth and stringency in what constitutes a new isoform. Specifically, Glinos et al.^19^ used gene annotations from 2016 when determining new isoforms. This is likely because they were trying to maintain consistency with previous Genotyope-Tisse Expression (GTEx) releases, but approximately 50,000 new isoforms have already been annotated since then^2^. We also set a stricter threshold for high-confidence isoforms, using a median CPM > 1. Given the depth of our data, a CPM = 1 corresponds to an average of 24 observed copies (that is, counts) per sample. Exactly 297 (69.4%) of our newly discovered isoforms are unique to our data, when compared with Ensembl v.107, Glinos et al.^19^ and Leung et al.^20^ (Supplementary Tables 2 and 3).

**Extended Data Fig. 4:**
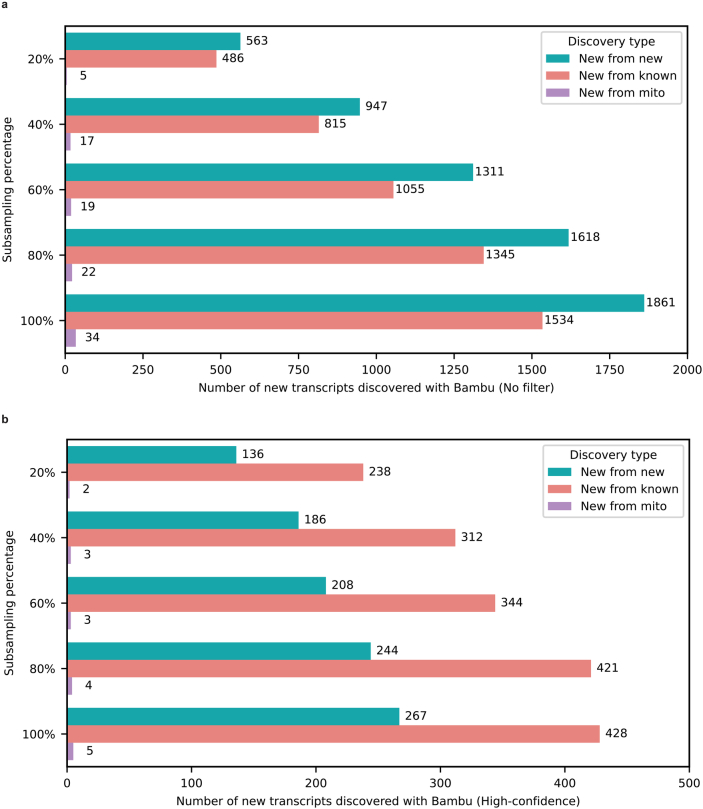
Number of newly discovered transcripts across subsampling range. **a**, Barplot showing the subsampling percentage on the Y-axis and number of new transcripts discovered with Bambu without filtering by expression estimates (no filter) on the X-axis. **b**, Barplot showing the subsampling percentage on the Y-axis and number of new transcripts discovered with Bambu when filtering by expression estimates X-axis (high-confidence; median CPM > 1). Nuclear encoded transcripts were filtered by median CPM > 1 and mitochondrially encoded transcripts were filtered by median full-length counts > 40. We used a different filter for mitochondrial transcripts due to issues in read assignment due to the polycistronic nature of mitochondrial transcription. The decline in identified new transcripts at lower sequencing depths was mostly due to Bambu’s filtering criteria, which demands enough evidence of unique and full-length reads to call a new transcript.

**Extended Data Fig. 5:**
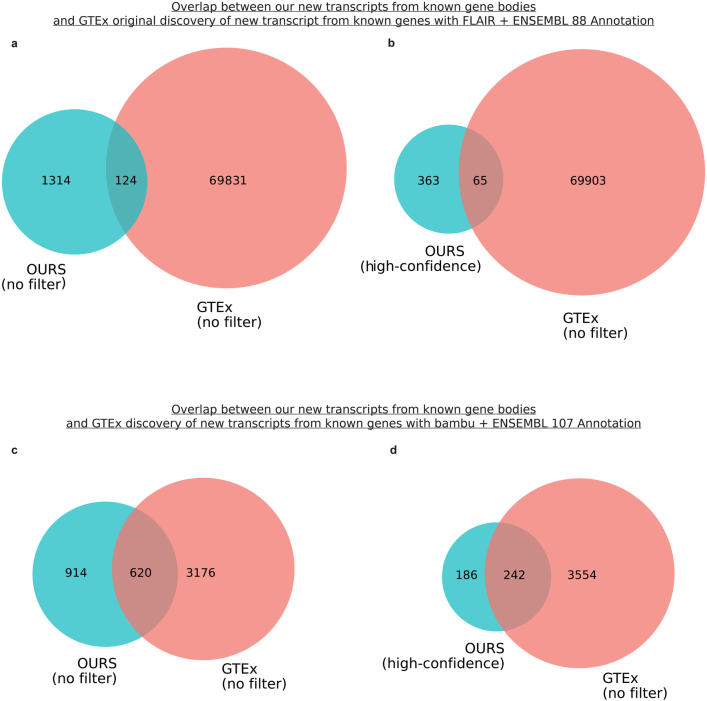
Difference in transcript discovery overlap based on annotation and computational tool used. **a**, Venn diagram showing the overlap between all our new transcripts from known gene bodies and new transcripts from known gene bodies in original GTEx long-read RNAseq article published by Glinos et al.^20^ using FLAIR for transcript discovery and ENSEMBL 88 annotation. **b**, Same as **a** but showing comparison only for new high-confidence transcripts from known gene bodies in our data. We used 70,000 as the number of new transcripts from known gene bodies in GTEx since they report just over 70,000 novel transcripts for annotated genes in their abstract. **c**, Venn diagram showing the overlap between all our new transcripts from known gene bodies and new transcripts from known gene bodies found when running GTEx long-read RNAseq data from article published by Glinos et al.^20^ using bambu for transcript discovery and ENSEMBL 107 annotation. **d**, Same as **a** but showing comparison only for new high-confidence transcripts from known gene bodies in our data. We analyzed data from all tissue types from the original Glinos et al. article to ensure consistency between our approaches. The discovery of new isoforms unique to GTEx when using the identical pipeline and annotations from our study likely results from tissue-specific isoforms that do not occur in the brain. Venn diagrams are not to scale to improve readability.

We performed a down-sampling analysis to assess the importance of depth on our discoveries. Including all discoveries (even those with median CPM ≤ 1), we discovered only 490 new isoforms from known genes with 20% of our aligned reads compared with 1,534 using 100% of our aligned reads (difference of 1,044; Extended Data Fig. 4a). Looking only at high-confidence discoveries in known genes, we discovered 238 and 428 at 20% and 100% of reads, respectively (Extended Data Fig. 4b), showing the importance of depth in our data. Although both annotations and read depth were important factors impacting new RNA isoform discovery, these do not explain the dramatic difference in reported discoveries between our work and that of Glinos et al.^19^. Thus, we conclude that the primary driver of these differences is the discovery tool employed. We observed a 33.8% increase in transcript discovery overlap between our dataset and GTEx when using the same tools and annotation, supporting the idea that these are large drivers of differences between our findings (Extended Data Fig. 5). We analyzed data from all tissue types from Glinos et al.^19^ to ensure consistency between our approaches. The discovery of new isoforms unique to GTEx when using the identical pipeline and annotations from our study probably results from tissue-specific isoforms that do not occur in the brain.

**Extended Data Fig. 6:**
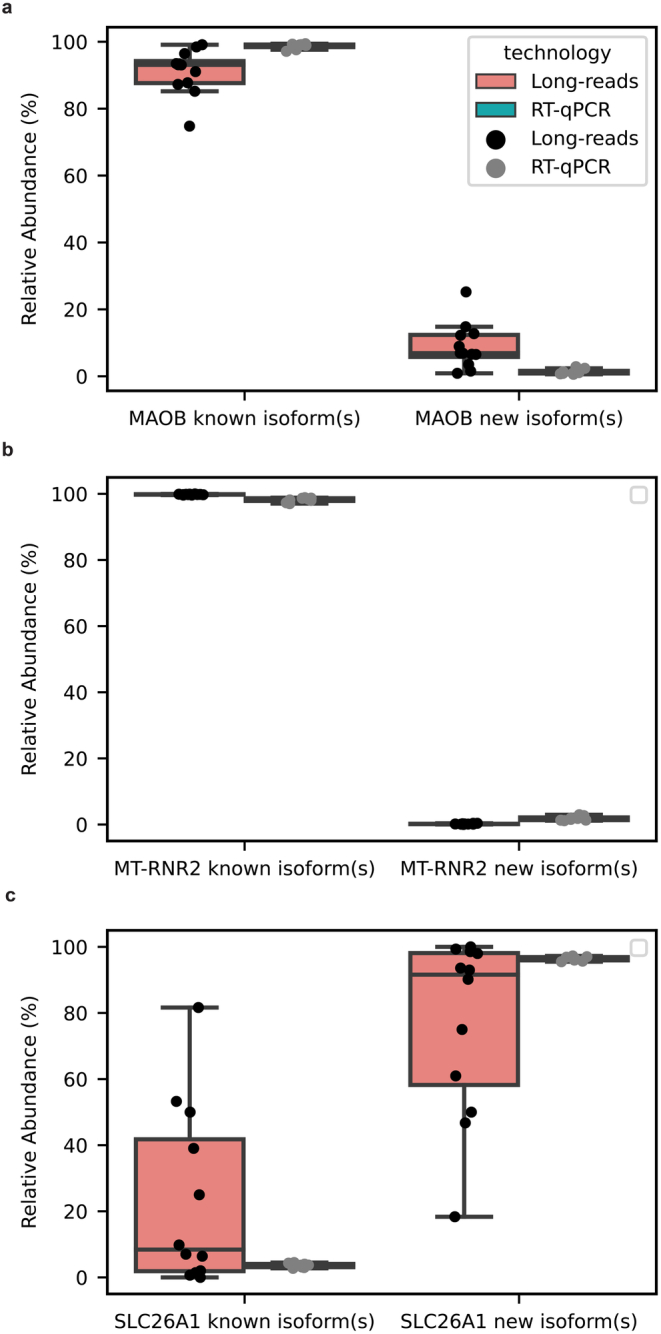
RT-qPCR validations for new RNA isoforms from MAOB, SLC26A1, *MT-RNR2* RNA isoforms match long-read sequencing data. **a**, Comparison of relative abundance between long-read sequencing and RT-qPCR for RNA isoforms in *MAOB*. **b**, Same as **a**, but for *MT-RNR2* **c**, Same as **a**, but for SLC26A1. Relative abundance was calculated as: ${Relative\; Abundance}=\frac{{Expression\; estimate\; for\; a\; given\; RNA\; isoform}}{\sum ({Expression\; estimates\; for\; RNA\; isoforms\; from\; the\; given\; gene})}* 100$ We used CPM (Counts Per Million) as the expression estimate for long-read sequencing and 2^(-∆Ct) for RT-qPCR. We used 2^-ΔCt^ as the expression estimate instead of the more common 2^-ΔΔCt^. This is because the 2^-ΔΔCt^ is optimized for comparisons between samples within the same gene/isoform, but does not work well for comparison between genes/isoforms. On the other hand, the 2^-ΔCt^ expression estimate allows for comparison between different genes/isoforms. The housekeeping gene for RT-qPCR was CYC1. For all figures in this panel the data labeled as technology long-reads comes from n = 12 biologically independent samples while the data labeled as technology RT-qPCR comes from n = 8 biologically independent samples. The eight samples from RT-qPCR are a subset of the 12 samples contained in long-reads. We only used eight samples for RT-qPCR because we ran out of brain tissue for the four of our samples. All boxplots in this panel follow this format: center line, median; box limits, upper and lower quartiles; whiskers, 1.5x interquartile range.

New high-confidence isoforms had a median of 761.5 nucleotides in length, ranging from 179 nt to 3,089 nt (Fig. 2c) and the number of exons ranged between 2 and 14, with most isoforms falling on the lower end of the distribution (Fig. 2d). Our data were enriched for new RNA isoforms containing all new exons and exon–exon boundaries (that is, exon junctions; Fig. 2e). The 428 new high-confidence isoforms contained 737 new exon–intron boundaries, where 94.9% (356/370) and 100% (367/367) of the 5′- and 3′-splice sites matched canonical splice site motifs, respectively, supporting their biological feasibility (Fig. 2f). We successfully validated 9 of 17 attempts for new high-confidence isoforms through PCR and gel electrophoresis (Fig. 2g, Supplementary Figs. 5–26 and Supplementary Table 4). Of the eight RNA isoforms that failed via standard PCR (no visible band on gel), six were validated through real-time quantitative PCR (RT–qPCR) using a conservative cutoff of *C*_t_ < 35 (ref. ^30^) (Supplementary Table 5). Of the 15 transcripts successfully validated through PCR and gel electrophoresis or RT–qPCR, 11 were unique to the present study. For additional validation, we compared relative abundance for known and new RNA isoforms between long-read sequencing and RT–qPCR for *MAOB*, *SLC26A1* and *MT-RNR2*. The expression patterns were concordant for all three genes tested (Extended Data Fig. 6 and Supplementary Tables 6 and 7).

**Extended Data Fig. 7:**
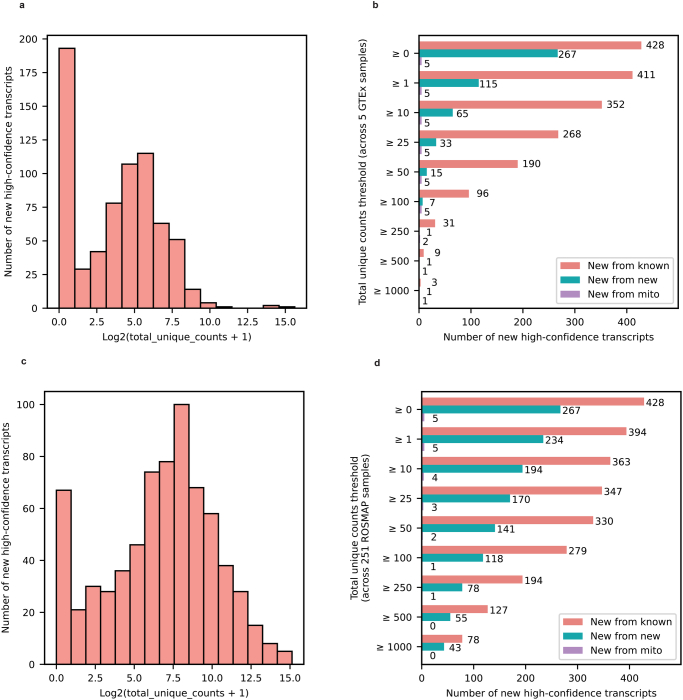
External validation of new high-confidence transcripts using publicly availabla data from 5 GTEx brain samples (Brodmann area 9) sequenced with long-read RNAseq and 251 ROSMAP brain samples (Brodmann area 9/46) sequenced with Illumina 150 bp paired-end RNAseq reads. **a**, Histogram showing total unique counts for new high-confidence transcripts across five GTEx long-read RNAseq data from brain samples. Total unique counts are shown in a log2(total unique counts + 1) scale to avoid streching generated by outliers. **b**, Barplot showing the number of new high-confidence transcripts that meet different total unique counts thresholds in cross-validation using five GTEx long-read RNAseq data from brain samples. The ‘≥ 0’ Y-axis label shows the total number of high-confidence transcripts before any filtering. Legend colors: New from known denotes new transcripts from known gene bodies, New from new denotes new transcripts from newly discovered gene bodies, and new from mito denotes new mitochondrially encoded spliced transcripts. **c**, Same as **a** but for 251 ROSMAP brain samples sequenced with 150 bp paired-end Illumina RNAseq. **d**, Same as **b** but for 251 ROSMAP brain samples sequenced with 150 bp paired-end Illumina RNAseq. We observed that 98.8% of the new high-confidence transcripts from known gene bodies had at least one uniquely mapped read in either GTEx or ROSMAP data and 69.6% had at least 100 uniquely mapped reads in either dataset. Over 94.4% of the new high-confidence transcripts from new gene bodies had at least one uniquely mapped read in either GTEx or ROSMAP data and over 44.2% had at least 100 uniquely mapped reads in either dataset.

We further attempted to validate our new high-confidence transcripts from known genes using long-read RNA-seq data from five GTEx^19^ brain samples (Brodmann area 9) and short-read RNA-seq data from 251 ROSMAP^25^ brain samples (Brodmann area 9/46). Approximately 98.8% of the new high-confidence transcripts from known gene bodies had at least one uniquely mapped read in either GTEx or ROSMAP data and 69.6% had at least 100 uniquely mapped reads in either dataset (Extended Data Fig. 7 and Supplementary Table 8).

**Extended Data Fig. 8:**
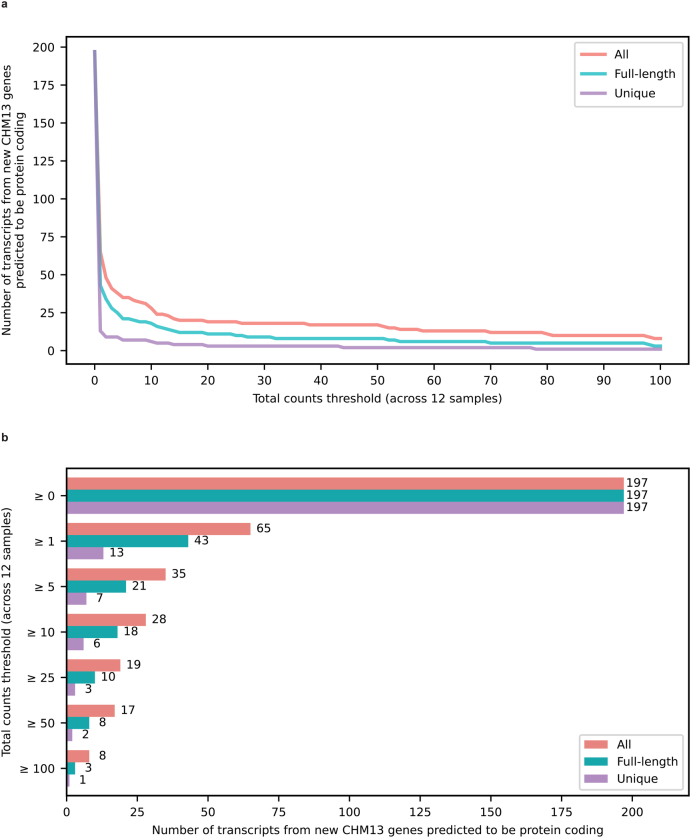
Expression of 197 transcripts from extra 99 predicted protein coding genes in CHM13 reported by Nurk et al. **a**, Lineplot with number of transcripts from extra 99 protein coding genes that are expressed across the total counts threshold for our 12 brain samples. The red line indicates all counts (including partial assignments), mint green line indicates full-length reads and purple line indicates unique reads. **b**, Barplot showing the number of transcripts from extra 99 protein coding genes expressed at or above different counts thresholds. The top y-axis label shows all the 197 annotated RNA isoforms from the extra 99 predicted protein coding genes in CHM13 reported by Nurk et al.

Out of interest, we also validated 6 RNA isoforms from the 99 newly predicted protein-coding genes reported in Nurk et al.^31^ using the new telomere-to-telomere (T2T) CHM13 reference genome (Extended Data Fig. 8). Our validation threshold for the CHM13 analysis was at least 10 uniquely mapped reads in total across our 12 frontal cortex samples.

Using MS data from the same brain region and human cell lines, we validated 11 of the new high-confidence isoforms from known genes at the protein level (Fig. 2h,i). Three of the eleven that we validated were unique to our study (BambuTx1879, BambuTx1758 and BambuTx2189).

#### Medically relevant genes

Identification and quantification of all isoforms are especially important for known medically relevant genes because, for example, when clinicians interpret the consequence of a genetic mutation, it is interpreted in the context of a single isoform of the parent gene body. That isoform may not even be expressed in the relevant tissue or cell type, however. Thus, knowledge about which tissues and cell types express each isoform will allow clinicians and researchers to better interpret the consequences of genetic mutations in human health and disease. To assess RNA isoform expression for medically relevant genes in the frontal cortex, we used the list of medically relevant genes defined in ref. ^32^, also adding genes relevant to brain-related diseases^33–42^.

**Fig. 3:**
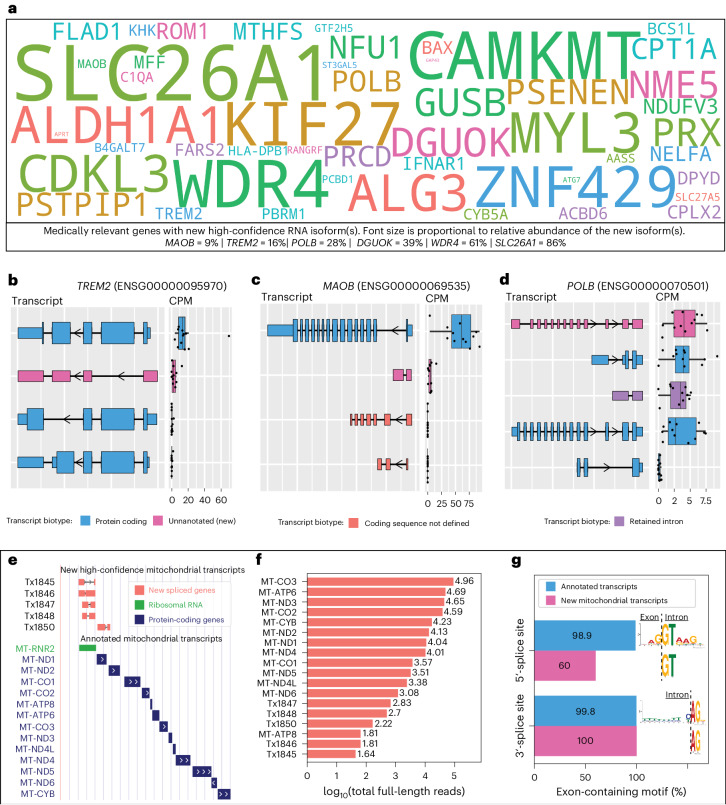
Medically relevant genes with new high-confidence RNA isoforms and new spliced, mitochondrially encoded RNA isoforms expressed in human frontal cortex. **a**, Gene names for medically relevant genes where we discovered a new RNA isoform that was not annotated in Ensembl v.107. It included only new RNA isoforms with a median CPM > 1. The size of the gene name is proportional to the relative abundance of the new RNA isoform. Relative abundance values relevant to this figure can be found in Supplementary Fig. 27. **b**–**d**, RNA isoform structure and CPM expression for isoforms from *TREM2* (**b**), *MAOB* (**c**) and *POLB* (**d**). For *TREM2* and *MAOB* all isoforms are shown (four each). For *POLB* only the top five most highly expressed isoforms in human frontal cortex are shown. **e**–**g**, New spliced, mitochondrially encoded transcripts. We included only new mitochondrial transcripts with median full-length counts >40. **e**, Structure for new spliced mitochondrial transcripts in red/coral denoted by ‘Tx’. MT-RNR2 ribosomal RNA is represented in green (overlapping four out of five spliced mitochondrial isoforms) and known protein-coding transcripts in blue. **f**, Bar plot showing number of full-length counts (log_10_) for new spliced mitochondrial transcripts and known protein-coding transcripts. **g**, Bar plot showing the prevalence of canonical splice site motifs for annotated exons from nuclear transcripts with median CPM > 1 versus new exon from spliced mitochondrial transcripts. All boxplots in this panel follow the following format: median (center line), quartiles (box limits), 1.5 × IQR (whiskers) (*n* = 12 biologically independent samples).

Of the 428 new high-confidence isoforms, 53 originated from 49 medically relevant genes and we quantified the proportion of total expression for the gene that came from the new isoform(s) (Fig. 3a and Supplementary Fig. 27). The genes with the largest percentage of reads from a newly discovered isoform include *SLC26A1* (86%; kidney stones^43^ and musculoskeletal health^44^), *CAMKMT* (61%; hypotonia–cystinuria syndrome, neonatal seizures, severe developmental delay and so on^45^) and *WDR4* (61%; microcephaly^46^ and Galloway–Mowat syndrome-6 (ref. ^47^)). Other notable genes with new high-confidence isoforms include *MTHFS* (25%; major depression, schizophrenia and bipolar disorder^48^), *CPLX2* (10%; schizophrenia, epilepsy and synaptic vesicle pathways^49^) and *MAOB* (9%; currently targeted for Parkinson’s disease treatment^50^; Fig. 3c). We also found an unannotated RNA isoform for *TREM2* (16%; Fig. 3b), one of the top AD risk genes^51^, which skips exon 2. This isoform was reported as new in our data because it remains unannotated by Ensembl as of June 2023 (ref. ^2^), but has previously been reported by two groups^52,53^. The articles identifying this new *TREM2* isoform reported a relative abundance of around 10%, corroborating our long-read sequencing results^52,53^. The new isoform for *POLB*—a gene implicated in base-excision repair for nuclear and mitochondrial genomes^54,55^—accounted for 28% of the gene’s expression (Fig. 3d). We discovered an additional 66 new transcripts from medically relevant genes with median CPM ≤ 1, including new RNA isoforms for *SMN1* and *SMN2* (spinal muscular atrophy^56^; Supplementary Figs. 28 and 29). Medically relevant genes with new RNA isoforms that did not meet our high confidence are shown in Supplementary Fig. 30.

#### Spliced, mitochondrially encoded isoforms

We identified a new set of spliced, mitochondrially encoded isoforms containing two exons (Fig. 3e), a highly unexpected result given that annotated mitochondrial transcripts contain only one exon. New mitochondrial isoforms were filtered using a count threshold based on full-length reads rather than a median CPM threshold owing to technical difficulties in quantification arising from the polycistronic nature of mitochondrial transcription. Bambu identified a total of 34 new spliced mitochondrial isoforms, but, after filtering using a strict median full-length count threshold of 40, only 5 high-confidence isoforms remained. Four of the new high-confidence isoforms span the *MT-RNR2* transcript. Not only does *MT-RNR2* encode the mitochondrial 16S rRNA, but it is also partially translated into a purported anti-apoptotic, 24-amino acid peptide (humanin) by inhibiting the Bax protein^57^. The fifth new high-confidence isoform spans the *MT-ND1* and *MT-ND2* genes, but on the opposite strand. Our results support previous important work by Herai et al. demonstrating splicing events in mitochondrial RNA^58^.

For context, although expression for the new mitochondrial isoforms was low compared with known mitochondrial genes (Fig. 3f), their expression was relatively high when compared with all nuclear isoforms. All five exons from new high-confidence mitochondrial isoforms contained the main nucleotides from the canonical 3′-splice site motif (AG), whereas three out of five (60%) contained the main nucleotides from the canonical 5′-splice site motif (GT) (Fig. 3g).

We attempted to validate three new high-confidence mitochondrially encoded isoforms through PCR and successfully validated two of them (Supplementary Figs. 25 and 26). It was not possible to design specific primers for the other two new high-confidence mitochondrial isoforms because of low sequence complexity or overlap with other lowly expressed (low-confidence) mtRNA isoforms found in our data. However, we were able to validate all five high-confidence spliced mitochondrial transcripts in the data from Glinos et al.^19^ because each had at least 100 uniquely aligned counts across each of the 5 GTEx brain samples (Extended Data Fig. 7). Mitochondria are essential to human cell life (and most eukaryotes) and have been implicated in a range of human diseases, including seizure disorders^59^, ataxias^60^, neurodegeneration^61^ and other age-related diseases^62^. Thus, although function for the new isoforms is not clear, determination of their function is important because they could have important biological roles or serve as biomarkers for mitochondrial function.

### Discovery of transcripts from new gene bodies

**Fig. 4:**
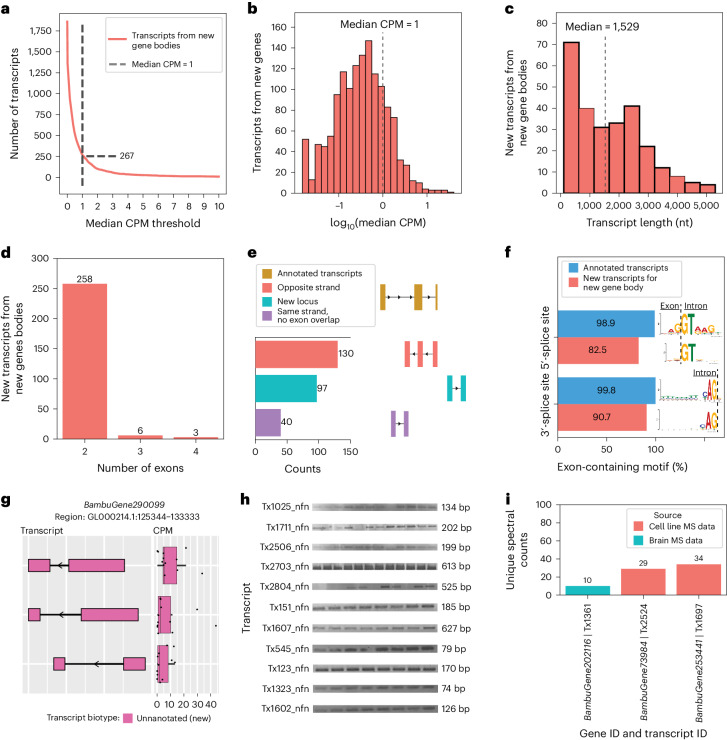
New high-confidence gene bodies in human frontal cortex tissue. **a**, Number of newly discovered transcripts from new gene bodies represented across the median CPM threshold. The cutoff is shown as the dashed line set at the median CPM = 1. **b**, Distribution of log_10_(median CPM values) for new transcripts from new gene bodies. The dashed line shows the cutoff point of the median CPM = 1. **c**–**g**, Data from transcripts above this expression cutoff. **c**, Histogram showing length distribution for new transcripts from new gene bodies. **d**, Bar plot showing the distribution of the number of exons for new transcripts from new gene bodies. Given the large proportion of transcripts containing only two exons, it is possible that we sequenced only a fragment of larger RNA molecules. **e**, Bar plot showing the kinds of events that gave rise to new transcripts from new gene bodies (in part created with BioRender.com). **f**, Bar plot showing the prevalence of canonical splice site motifs for annotated exons from transcripts with a median CPM > 1 versus new exons from new gene bodies. **g**, RNA isoform structure and CPM expression for isoforms from new gene body (*BambuGene290099*). Boxplot format: median (center line), quartiles (box limits), 1.5 × IQR (whiskers) (*n* = 12 biologically independent samples). **h**, Gel electrophoresis validation using PCR amplification for a subset of new isoforms from new genes. This is an aggregate figure showing bands for several different gels. Each gel electrophoresis PCR experiment was independently performed once with similar results. Individual gel figures are available in Supplementary Figs. 5–26. **i**, Protein level validation using publicly available MS proteomics data. The *y* axis shows the number of spectral counts from uniquely matching peptides (unique spectral counts); new transcripts from new genes were considered to be validated at the protein level if they had more than five unique spectral counts.

RNA isoforms from new gene bodies refer to poly(adenylated) RNA species coming from regions of the genome where transcription was unexpected (that is, unannotated). Bambu identified a total of 1,860 isoforms from 1,676 new gene bodies. We observed a total of 1,593 potential new gene body isoforms with a CPM ≤ 1. We considered these potential discoveries as low confidence and excluded them from the remainder of our analyses, leaving 267 high-confidence isoforms from 245 gene bodies (Fig. 4a,b). Glinos et al.^19^ did not specifically report on new gene bodies, but Leung et al.^20^ reported 54 new gene bodies in human cortex where 5 overlapped with our high-confidence isoforms from new genes. The new isoforms from new gene bodies had a median length of 1,529 nt, ranging between 109 nt and 5,291 nt (Fig. 4c). The number of exons ranged between 2 and 4, with 96.6% of isoforms having only 2 exons (Fig. 4d). Given the large proportion of transcripts containing only two exons, it is possible that we sequenced only a fragment of larger RNA molecules.

Of the 267 new high-confidence isoforms from new gene bodies, 130 overlapped a known gene body on the opposite strand, 97 came from a completely new locus and 40 came from within a known gene body, but did not overlap a known exon (Fig. 4e). These 170 new transcripts from new gene bodies located in intragenic regions could be a result of leaky transcription and splicing. A recent article^63^ suggests that spurious intragenic transcription may result from aging in mammalian tissues. In new isoforms from new gene bodies, 82.5% (222 of 269) of exons contained the primary ‘GT’ nucleotides from the canonical 5′-splice site motif, whereas 90.7% (244 of 269) contained the primary ‘AG’ nucleotides from the canonical 3′-splice site motif (Fig. 4f). It is interesting that one new gene body (*BambuGene290099*) had three high-confidence RNA isoforms (Fig. 4g). We successfully validated 11 of 12 attempts for new high-confidence RNA isoforms from new gene bodies through PCR and gel electrophoresis (Fig. 4h, Supplementary Figs. 5–26 and Supplementary Table 4), where the 12th was successfully validated through RT–qPCR (mean *C*_t_ = 23.2; Supplementary Table 5). All 12 new RNA isoforms from new gene bodies validated through PCR were unique to the present study.

Over 94.4% of the new high-confidence transcripts from new gene bodies had at least one uniquely mapped read in either GTEx or ROSMAP data and >44.2% had at least 100 uniquely mapped reads in either dataset (Extended Data Fig. 7 and Supplementary Table 8). The validation rate for new transcripts from known gene bodies was higher than new transcripts from new gene bodies, indicating that some of our newly discovered genes could be aging related. Whether these newly discovered gene bodies are biologically meaningful or ‘biological noise’ is unclear. We validated three RNA isoforms from new gene bodies at the protein level using MS data from the same brain region and human cell lines (Fig. 4i); all three were unique to the present study.

During isoform discovery, we identified a new low-abundance RNA isoform (median CPM < 1) with two exons for the External RNA Controls Consortium (ERCC) RNA spike-ins (Supplementary Figs. 31 and 32). We were skeptical about this discovery because ERCCs contain only one exon, but we validated these results by PCR across two different batches of ERCC (Supplementary Figs. 33 and 34).

### Medically relevant genes expressing multiple RNA isoforms

**Fig. 5:**
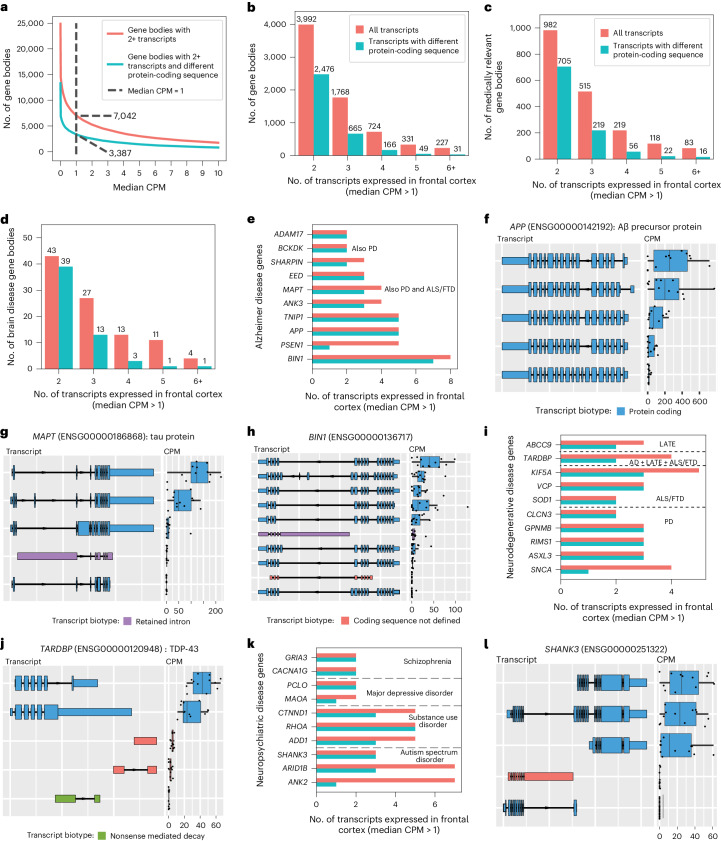
Gene bodies expressing multiple transcripts in the frontal cortex. **a**, Gene bodies with multiple transcripts across the median CPM threshold. **b**–**i**, Gene bodies with multiple transcripts at median CPM > 1. **b**, Gene bodies expressing multiple transcripts. **c**, Medically relevant gene bodies expressing multiple transcripts. **d**, Brain disease-relevant gene bodies expressing multiple transcripts. **e**, Transcripts expressed in the frontal cortex for a subset of genes implicated in AD. **f**, *APP* transcript expression. **g**, *MAPT* transcript expression. **h**, *BIN1* transcript expression. **i**, Same as **e** but for genes implicated in other neurodegenerative diseases. LATE, limbic-predominant, age-related *TDP-43* encephalopathy. **j**, *TARDBP* transcript expression. **k**, Same as **e** but for genes implicated in neuropsychiatric disorders. In **i** and **k**, the dashed lines are delimiters, separating the genes that are associated with different brain-related disorders. **l**, *SHANK3* transcript expression. Boxplot format for entire panel: median (center line), quartiles (box limits), 1.5 × IQR (whiskers) (*n* = 12 biologically independent samples).

We found 7,042 genes expressing two or more RNA isoforms with a median CPM > 1, where 3,387 genes expressed ≥2 isoforms with distinct protein sequences (Fig. 5a,b). Of the 5,035 medically relevant genes included in the present study^32^, 1,917 expressed multiple isoforms and 1,018 expressed isoforms with different protein-coding sequences (Fig. 5c), demonstrating the isoform diversity of medically relevant genes in a single tissue and the importance of interpreting genetic variants in the proper context of tissue-specific isoforms. Of the 7,418 transcripts from medically relevant genes expressed with median CPM > 1, 5,695 are longer than 2,000 nt (Supplementary Fig. 35). Given the length of these 5,695 RNA isoforms, it is likely that their quantification is less accurate, despite the advantages that long-read sequencing offers.

It is interesting that 98 genes implicated in brain-related diseases expressed multiple RNA isoforms in human frontal cortex, including AD genes such as *APP* (Aβ-precursor protein) with 5, *MAPT* (tau protein) with 4 and *BIN1* with 8 (Fig. 5d–h). Notably, we observed only four *MAPT* isoforms with a median CPM > 1, where two were expressed at levels many times greater than the others, whereas substantial previous research suggests that there are six tau proteins expressed in the central nervous system^64–66^. Similarly, several genes implicated in other neurogenerative diseases and neuropsychiatric disorders expressed multiple isoforms in human frontal cortex, including *SOD1* (amyotrophic lateral sclerosis (ALS) and frontotemporal dementia (FTD); Fig. 5i) with two isoforms expressed with a median CPM > 1, *SNCA* (Parkinson’s disease (PD); Fig. 5i) with four, *TARDBP* (TDP-43 protein; involved in several neurodegenerative diseases; Fig. 5i,j) with four and *SHANK3* (autism spectrum disorder; Fig. 5k,l) with three.

### RNA isoform expression reveals patterns hidden at gene level

Perhaps the most compelling value in long-read RNA-seq is the ability to perform differential isoform expression analyses. Through these analyses, we can begin to distinguish which isoforms are expressed in specific cell types and tissue types and ultimately determine their associations with human health and disease. Thus, as proof of principle, we performed differential gene and isoform expression analyses comparing six pathologically confirmed cases of AD and six cognitively unimpaired controls. The dataset is not large enough to draw firm disease-specific conclusions, but it does demonstrate the need for larger studies.

**Fig. 6:**
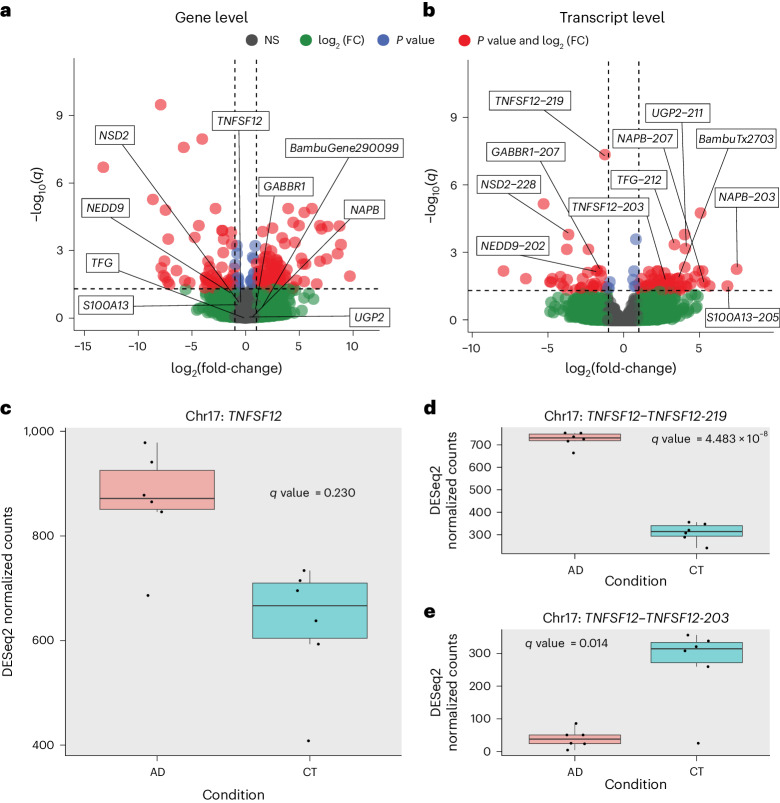
RNA isoform analysis can reveal disease expression patterns unavailable at the gene level. **a**, Differential gene expression between cases with AD and cognitively unimpaired controls. The horizontal line is at the FDR-corrected *P* value (*q* value) = 0.05. Vertical lines are at log_2_(fold-change) = −1 and +1. The threshold for differential gene expression was set at *q* value < 0.05 and log_2_(fold-change) > 1. The names displayed represent a subset of genes that are not differentially expressed but have at least one RNA isoform that is differentially expressed. FC, fold-change; NS, not significant. **b**, Same as **a** but for differential RNA isoform expression analysis. We used the DESeq2 R package with two-sided Wald’s test for statistical comparisons and the Benjamini–Hochberg correction for multiple comparisons in the differential expression analyses presented in **a** and **b**. **c**, Expression for *TNFSF12* between cases with AD and controls (CT). The *TNFSF12* gene does not meet the differential expression threshold. **d**, *TNFSF12-219* transcript expression between AD and CT. *TNFSF12-219* is upregulated in AD. **e**, Expression for the *TNFSF12-203* transcript between AD and CT. *TNFSF12-203* is upregulated in CT. All boxplots in this panel follow the following format: center line, median; box limits, upper and lower quartiles; whiskers, 1.5 × IQR. All figures come from *n* = 12 biologically independent samples (AD, *n* = 6; CT, *n* = 6).

We found 176 differentially expressed genes and 105 differentially expressed RNA isoforms (Fig. 6a,b and Supplementary Tables 9 and 10). Of these 105 isoforms, 99 came from genes that were not differentially expressed when collapsing all isoforms into a single gene measurement (Fig. 6a,b), demonstrating the utility of differential isoform expression analyses. It is interesting that there were two differentially expressed isoforms from the same gene (*TNFSF12*), with opposite trends. The TNFSF12-219 isoform was upregulated in cases with AD whereas TNFSF12-203 was upregulated in controls (Fig. 6c–e), even though the *TNFSF12* gene was not differentially expressed when collapsing all transcripts into a single gene measurement (Fig. 6c).

**Extended Data Fig. 9:**
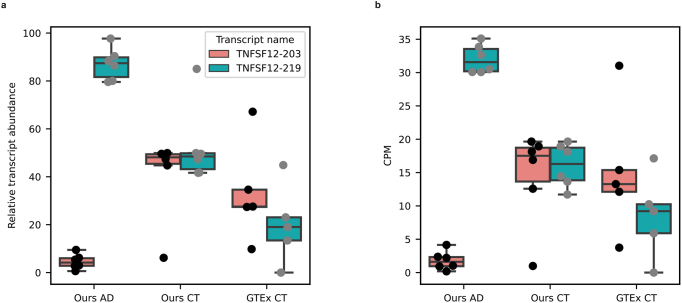
Attempt at validation of TNFSF12 RNA isoform expression pattern in healthy controls. **a**, Boxplot showing the relative transcript abudance (percentage) for TNFSF12 RNA isoforms that are differentially expressed between Alzheimer’s disease cases and controls in this study. On the X-axis, the ‘OURS AD’ label represents data from six (n = 6) biologically independent Alzheimer’s disease brain samples sequenced in this study. The ‘OURS CT’ label represents data from six (n = 6) biologically independent cognitively unimpaired aged control brain samples sequenced in this study. The ‘GTEx CT’ label label represents data from five (n = 5) biologically independent GTEx brain samples (Brodmann area 9) sequenced with PCR amplified long-read nanopore RNAseq by Glinos et. al. **b**, Boxplot showing the CPM for TNFSF12 RNA isoforms that are differentially expressed between Alzheimer’s disease cases and controls in this study. X-axis labels follow the same pattern as **a** and labels represent the same groups as in **a**. All boxplots in this panel follow this format: center line, median; box limits, upper and lower quartiles; whiskers, 1.5x interquartile range.

Out of interest, we measured the expression patterns for the *TNFSF12-203* and *TNFSF12-219* isoforms in the five GTEx long-read RNA-seq samples from Brodmann area 9 to assess whether the expression pattern matched what we observed in our cognitively unimpaired controls (Extended Data Fig. 9). We found that the expression for both *TNFSF12* isoforms shows greater variability than either of our groups, but arguably more closely resembles the pattern in our controls.

Out of interest, we also provided plots from a principal component analysis at both the gene and the isoform level where we observed a potential separation between cases and controls (Supplementary Fig. 36). We encourage caution to avoid overinterpreting this potential separation between cases and controls given the small sample size.

## Discussion

By applying deep long-read RNA-seq, we identified new gene bodies and RNA isoforms expressed in human frontal cortex, demonstrating that substantial gaps remain in our understanding of RNA isoform diversity (Figs. 2a, 3e and 4a). We quantified the individual RNA isoform expression levels in human frontal cortex as a step toward functional analysis of these isoforms. We found 7,042 genes expressing multiple RNA isoforms, with 1,917 being medically relevant genes (that is, implicated in human disease; Fig. 5a–c). Some of these medically relevant genes expressing multiple RNA isoforms in human frontal cortex are implicated in brain-related diseases, including AD, PD, autism spectrum disorder, substance use disorder and others (Fig. 5d). Together, these findings highlight the importance of measuring individual RNA isoform expression accurately to discern the possible roles of each isoform within human health and disease, and to interpret the effects of a given genetic variant.

We performed differential RNA isoform expression analysis to reveal expression patterns associated with disease that were hidden when performing gene-level analysis (Fig. 6a,b). Given the 99 isoforms that were differentially expressed where the gene as a whole was not, we demonstrated that performing differential gene-level expression is important, but may be insufficient in many cases if we want to truly understand the biological complexities afforded by alternative splicing. We further suggest that deep long-read RNA-seq is necessary to understand the full complexity of transcriptional changes during disease. The gene *TNFSF12* is a key example because, although the gene itself is not differentially expressed in our data, the *TNFSF12-219* isoform is significantly upregulated in cases with AD whereas the *TNFSF12-203* isoform is significantly upregulated in controls (Fig. 6c–e).

We also identified five new high-confidence, spliced mitochondrially encoded RNA isoforms with two exons each. This is a surprising finding given that all annotated human mitochondrial transcripts have only one exon (Fig. 2e,f). Previous work in human cell cultures corroborates our findings^58^. To our knowledge, no previous study has identified spliced mtRNA isoform expression directly in human tissue. Given the involvement of mitochondria in many age-related diseases^62^, it would be of interest to determine the function, if any, of these spliced mtRNA isoforms.

**Extended Data Fig. 10:**
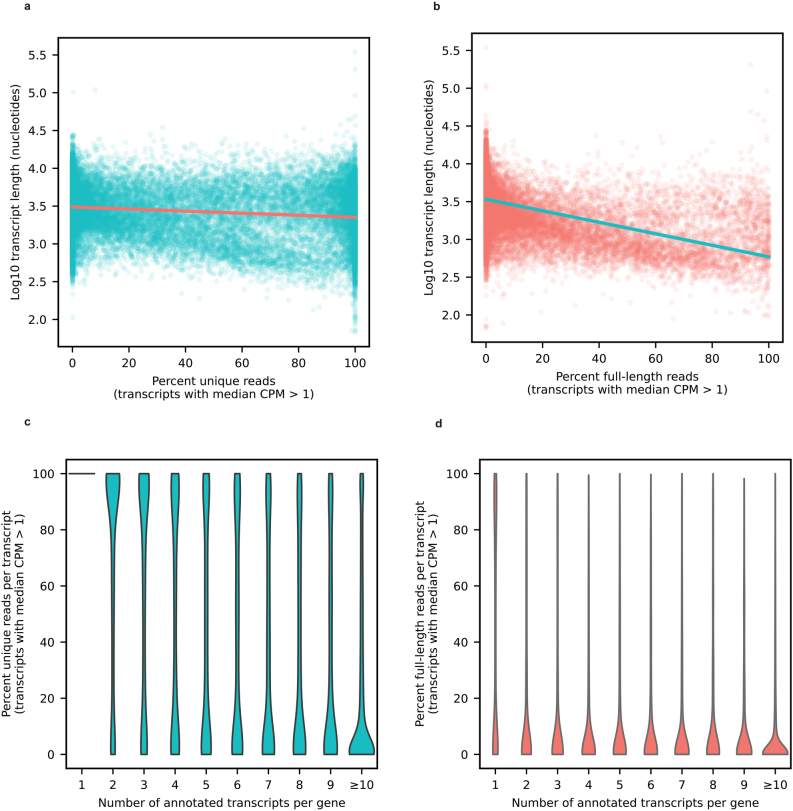
Percentage of unique and full-length reads per transcript. **a**, Scatterplot showing the percentage of uniquely aligned reads for each transcript with a median CPM > 1 on the X-axis and the Log10 transcript length on the Y axis. **b**, Scatterplot showing the percentage of full-length reads for each transcript with a median CPM > 1 on the X-axis and the Log10 transcript length on the Y axis. **c**, Violin plot showing the percentage of uniquely aligned reads for each transcript with median CPM > 1 on the Y-axis and the number of annotated transcript per gene on the X-axis. **d**, Violin plot showing the percentage of full-length reads for each transcript with median CPM > 1 on the Y-axis and the number of annotated transcript per gene on the X-axis. The percentage of full-length reads is more affected by increases in transcript length whereas the percentage of unique reads is more affected by increases in the number of annotated transcripts for a given gene.

Long reads present an improvement over short-read RNA-seq, but it remains challenging to accurately quantify RNA isoforms in genes with many large and similar isoforms (Extended Data Fig. 10). Thus, although this work is a substantial improvement over short-read sequencing, the data are not perfect and future improvements in sequencing, transcriptome annotation and bioinformatic quantification will continue to improve the accuracy of long-read RNA-seq. Our data showed a pronounced 3′ bias that can hinder RNA isoform quantification, especially for genes where the exon diversity is closer to the 5′-end (Supplementary Fig. 37).

The small sample size limits the generalizability of the differential RNA isoform expression results, serving primarily as a proof of concept for the value of measuring individual RNA isoform expression in disease tissue. We refrained from performing differential isoform usage analysis and pathway analysis to avoid overinterpreting results from only 12 samples; however, these analyses could provide valuable insights in larger studies. In addition, the present study is based on ‘bulk’ RNA-seq, rather than single-cell sequencing; bulk sequencing is likely to obscure critical cell type-specific expression patterns that single-cell sequencing can elucidate, although the cost of single-cell sequencing combined with long-read sequencing is still a major hurdle in making a large study of this kind feasible.

In conclusion, we demonstrate that a large proportion of medically relevant genes express multiple RNA isoforms in human frontal cortex, with many encoding different protein-coding sequences that could potentially perform different functions. We also demonstrate that differential RNA isoform analysis can reveal transcriptomic signatures in AD that are not available at the gene level. Our study highlights the advantage of long-read RNA-seq in assessing RNA expression patterns in complex human diseases to identify new molecular targets for treatment and diagnosis.

## Methods

### Sample collection, RNA extraction and quality control

Frozen postmortem, human frontal cortex brain samples were collected at the University of Kentucky Alzheimer’s Disease Research Center autopsy cohort^67^, snap-frozen in liquid nitrogen at autopsy and stored at −80 °C. Postmortem interval (from death to autopsy) was <5 h in all samples. All samples came from white individuals. Approximately 25 mg of gray matter from the frontal cortex was chipped on dry ice into prechilled, 1.5-ml low-bind tubes (Eppendorf, cat. no. 022431021), kept frozen throughout the process and stored at −80 °C. RNA was extracted using the Lexogen SPLIT RNA extraction kit (cat. no. 008.48) using protocol v.008UG005V0320 (Supplementary Information, pp. 51–75).

Briefly, ~25 mg of tissue was removed from −80 °C storage and kept on dry ice until processing began. Then, 400 μl of chilled isolation buffer (4 °C; Lexogen SPLIT RNA kit) was added to each tube and the tissue homogenized using a plastic pestle (Kontes Pellet Pestle, VWR, cat. no. KT749521-1500). Samples remained on ice to maintain RNA integrity while other samples were homogenized. Samples were then decanted into room-temperature, phase-lock gel tubes, 400 μl of chilled phenol (4 °C) was added and the tube inverted 5× by hand. Acidic buffer (AB, Lexogen), 150 μl, was added to each sample, the tube inverted 5× by hand before 200 μl of chloroform was added and inverted for 15 s. After a 2-m incubation at room temperature, samples were centrifuged for 2 min at 12,000*g* and 18–20 °C and the upper phase (approximately 600 μl) was decanted in a new 2-ml tube. Total RNA was precipitated by the addition of 1.75× the volume of isopropanol to the sample and then loaded on to a silica column by centrifugation (12,000*g*, 18 °C for 20 s; flow-through discarded). The column was then washed twice with 500 μl of isopropanol and 3× with 500 μl of wash buffer (Lexogen), while the column was centrifuged (12,000*g*, 18 °C for 20 s; flow-through discarded each time). The column was transferred to a new low-bind tube and the RNA eluted by the addition of 30 μl of elution buffer (incubated for 1 min and then centrifuged at 12,000*g*, 18 °C for 60 s) and the eluted RNA immediately placed on ice to prevent degradation.

RNA quality was determined initially by nanodrop (*A*_260_:*A*_280_ and *A*_260_:*A*_230_ absorbance ratios) and then via Agilent Fragment Analyzer 5200 using the RNA (15 nt) DNF-471 kit (Agilent). All samples achieved nanodrop ratios >1.8 and fragment analyzer RIN > 9.0 before sequencing (Supplementary Figs. 38–49 and Supplementary Table 1).

### RNA spike-ins

ERCC RNA spike-in controls (Thermo Fisher Scientific, cat. no. 4456740) were added to the RNA at the point of starting cDNA sample preparation at a final dilution of 1:1,000.

### Library preparation, sequencing and base calling

Isolated RNA was kept on ice until quality control testing was completed as described above. Long-read cDNA library preparation commenced, utilizing the Oxford Nanopore Technologies PCR-amplified cDNA kit (cat. no. SQK-PCS111). The protocol was performed according to the manufacturer’s specifications, with two notable modifications being that the cDNA PCR amplification expansion time was 6 min and we performed 14 PCR amplification cycles. Poly(A) enrichment is inherent to this protocol and happens at the start of the cDNA synthesis. The cDNA quality was determined using an Agilent Fragment Analyzer 5200 and Genomic DNA (50 kb) kit (Agilent DNF-467) (see Supplementary Figs. 50–61 for cDNA traces). The cDNA libraries were sequenced continuously for 60 h on the PromethION P24 platform with flow cell R9.4.1 (one sample per flow cell). Data were collected using MinKNOW v.23.04.5. The.fast5 files obtained were base called using the Guppy graphics processing unit (GPU) base-caller v.3.9 with configuration dna_r9.4.1_450bps_hac_prom.cfg.

### Read preprocessing, genomic alignment and quality control

Nanopore long-read sequencing reads were preprocessed using pychopper^68^ v.2.7.2 with the PCS111 sequencing kit setting. Pychopper filters out any reads not containing primers on both ends and rescues fused reads containing primers in the middle. Pychopper then orients the reads to their genomic strand and trims the adapters and primers off the reads.

The preprocessed reads were then aligned to the GRCh38 human reference genome (without alternative contigs and with added ERCC sequences) using minimap2 (ref. ^69^) v.2.22-r1101 with parameters ‘-ax splice -uf’. Full details and scripts are available on our GitHub (‘Code availability’). Aligned reads with a mapping quality (MAPQ) score <10 were excluded using SAMtools^70^ v.1.6. Secondary and supplementary alignments were also excluded using SAMtools v.1.6. The resulting bam alignment files were sorted by genomic coordinate and indexed before downstream analysis. Quality control reports and statistics were generated using PycoQC^71^ v.2.5.2. Information about mapping rate and read length and other sequencing statistics can be found in Supplementary Table 1 and Supplementary Figs. 1–4.

### Transcript discovery and quantification

Filtered BAM files were utilized for transcript quantification and discovery using bambu^14^ v.3.0.5. We ran bambu using Ensembl^2^ v.07, a gene transfer format (GTF) annotation file, with added annotations for the ERCC spike-in RNAs and the GRCh38 human reference genome sequence with added ERCC sequences. The BAM file for each sample was individually preprocessed with bambu and the resulting 12 RDS (R data serialization) files were provided as input all at once to perform transcript discovery and quantification using bambu. The new discovery rate (NDR) was determined based on the recommendation by the bambu machine learning model (NDR = 0.288). Bambu outputs three transcript-level count matrices, including total counts (all counts including reads that were partially assigned to multiple transcripts), unique counts (only counts from reads that were assigned to a single transcript) and full-length reads (only counts from reads containing all exon–exon boundaries from its respective transcript). Except where specified otherwise, expression values reported in this article come from the total count matrix.

We used full-length reads for quantification in the mitochondria because the newly discovered spliced mitochondrial transcripts caused issues in quantification. Briefly, owing to polycistronic mitochondrial transcription, many nonspliced reads were partially assigned to spliced mitochondrial transcripts, resulting in a gross overestimation of spliced mitochondrial transcript expression values. We bypassed this issue by using only full-length counts (that is, counting only reads that match the exon–exon boundaries of newly discovered spliced mitochondrial transcripts).

We included only newly discovered (that is, unannotated) transcripts with a median CPM > 1 in downstream analysis (that is, high-confidence new transcripts) unless explicitly stated otherwise. New transcripts from mitochondrial genes were the exception, being filtered using a median full-length reads >40 threshold.

Data from transcriptomic analysis can be visualized in the web application we created using R v.4.2.1 and Rshiny v.1.7.4: https://ebbertlab.com/brain_rna_isoform_seq.html.

### Analysis using CHM13 reference

We processed the RNA-seq data from the 12 dorsolateral, prefrontal cortex samples (Brodman area 9/46) from the present study using the same computational pipeline described above and below, except for two changes: (1) we used the CHM13 reference genome rather than GRCh38 and (2) we set bambu to quantification-only mode rather than quantification and discovery. The reference fasta and gff3 files were retrieved from the T2T-CHM13 GitHub (https://github.com/marbl/CHM13). The following are the links to the reference genome sequence (https://s3-us-west-2.amazonaws.com/human-pangenomics/T2T/CHM13/assemblies/analysis_set/chm13v2.0.fa.gz) and the GFF3 annotation (https://s3-us-west-2.amazonaws.com/human-pangenomics/T2T/CHM13/assemblies/annotation/chm13.draft_v2.0.gene_annotation.gff3). We then quantified expression for the extra 99 predicted protein-coding genes from CHM13 reported in Nurk et al.^31^.

### Subsampling discovery analysis

Nanopore long-read sequencing data were randomly subsampled at 20% increments, generating the following subsamples for each sample: 20%, 40%, 60% and 80%. The 12 subsampled samples for each increment were run through our long-read RNA-seq discovery and quantification pipeline described above and below. We compared the number of discovered transcripts between the subsamples and the full samples to assess the effect of read depth on the number of transcripts discovered using bambu. The CPM values were re-calculated based on the new sequencing depth for each subsampling increment, so the absolute count threshold to reach median CPM > 1 became lower as the sequencing depth decreased.

### Transcript discovery GTEx data with bambu

We obtained the long-read RNA-seq data from 90 GTEx samples across 15 human tissues and cell lines sequenced with the Oxford Nanopore Technologies, PCR-amplified cDNA protocol (PCS109) generated by Glinos et al.^19^. We then processed these data through our long-read RNA-seq discovery and quantification pipeline described above and below. We used the same Ensembl v.88 annotations originally used in Glinos et al.^19^ and compared the results between the original Glinos et al.^19^ results and the results from our data to assess the effect of the isoform discovery tool (that is, bambu^14^ versus FLAIR^28^) on the number of newly discovered transcripts. We also compared the number of newly discovered transcripts when running GTEx data through our computational pipeline with the Ensembl v.88 annotation and the Ensembl v.107 annotation to assess the effect of different annotations in the number of transcripts discovered. Last, we compared the overlap between new transcripts from known genes discovered in our study using 12 brain samples with the original results^19^ and the results we obtained from running the GTEx data through our computational pipeline using the Ensembl v.107 annotations.

### Validation of new transcripts using GTEx data

We obtained publicly available GTEx, nanopore, long-read RNA-seq data from six brain samples (Brodmann area 9). One of the samples was excluded because it had <50,000 total reads, so 5 samples were used for all downstream analysis. These data had been previously analyzed in Glinos et al.^19^. Fastq files were preprocessed using pychopper^68^ v.2.7.2 with the PCS109 sequencing kit setting. Downstream from that the files were processed as described above and below, except for two changes: (1) we set bambu to quantification-only mode and (2) we used a GTF annotation file containing all transcripts from Ensembl v.107, the ERCC spike-in RNAs and all the new transcripts discovered in the present study. The transcript-level unique count matrix outputted by bambu was utilized for validating the newly discovered transcripts in the present study.

### Validation of new transcripts using ROSMAP data

We obtained publicly available ROSMAP (Illumina), 150-bp paired-end RNA-seq data from 251 brain samples (Brodmann area 9/46). These data had been previously analyzed in ref. ^25^ and described in ref. ^26^. Fastq files were preprocessed and quality controlled using trim galore v.0.6.6. We generated the reference transcriptome using the GTF annotation file containing all transcripts from Ensembl v.107, the ERCC spike-in RNAs and all the new transcripts discovered in the present study. We used this annotation in combination with the GRCh38 reference genome and gffread v.0.12.7 to generate our reference transcriptome for alignment. The preprocessed reads were then aligned to this reference transcriptome using STAR^72^ v.2.7.10b. Full details and scripts are available on our GitHub (‘Code availability’). Aligned reads with a MAPQ score <255 were excluded using SAMtools^70^ v.1.6, keeping only reads that uniquely aligned to a single transcript. We quantified the number of uniquely aligned reads using salmon^73^ v.0.13.1. The count matrix containing uniquely aligned read counts outputted by salmon was utilized for validating the newly discovered transcripts in the present study.

### Splice site motif analysis

We utilized the online meme suite tool^74^ v.5.5.3 (https://meme-suite.org/meme/tools/meme) to create canonical 5′- and 3′-splice site motifs and estimated the percentage of exons containing these motifs. For known genes, we included only exons from multi-exonic transcripts that were expressed with a median CPM > 1 in our samples. If two exons shared a start or an end site, one of them was excluded from the analysis. For new high-confidence transcripts, we filtered out any exon start or end sites contained in the Ensembl annotation. If two or more exons shared a start or an end site, we used only one of those sites for downstream analyses. For the 5′-splice site analysis, we included the last 3 nt from the exon and the first 6 nt from the intron. For the 3′-splice site analysis, we included the last 10 nt from the intron and the first 3 nt from the exon. The coordinates for 5′- and 3′-splice site motifs were chosen based on previous studies^75,76^. The percentage of exons containing the canonical 5′-splice site motif was calculated using the proportion of 5′-splice site sequences containing GT as the two last nucleotides in the intron. The percentage of exons containing the canonical 3′-splice site motif was calculated by taking the proportion of 3′-splice site sequences containing AG as the first 2 nt in the intron. Fasta files containing 5′-splice site sequences from each category of transcript ((1) known transcript from known gene body, (2) new transcript from known gene, (3) new transcript from new gene body and (4) transcript from mitochondrial gene body) were individually submitted to the online meme suite tool to generate splice site motifs. The same process was repeated for 3′-splice site sequences. Owing to the small number of transcripts, it was not possible to generate reliable splice site motif memes for new transcripts from mitochondrial transcripts; instead we just used the 5′-GT sequence and 3′-AG sequence to represent them in Fig. 2g.

### Comparison between annotations

Annotations from new high-confidence transcripts discovered in the present study were compared with annotations from previous studies using gffcompare^77^ v.0.11.2. Transcripts were considered to overlap when gffcompare found a complete match of the exon–exon boundaries (that is, intron chain) between two transcripts. The annotation from Glinos et al.^19^ was retrieved from https://storage.googleapis.com/gtex_analysis_v9/long_read_data/flair_filter_transcripts.gtf.gz. The annotation from Leung et al.^20^ was retrieved from https://zenodo.org/record/7611814/preview/Cupcake_collapse.zip#tree_item12/HumanCTX.collapsed.gff.

### Differential gene expression analysis

Although bambu outputs a gene-level count matrix, this matrix includes intronic reads. To create a gene-level count matrix without intronic reads, we summed the transcript counts for each gene using a customized Python script (v.3.10.8). This gene-level count matrix without intronic reads was used for all gene-level analysis in the present study. We performed differential gene expression analysis only on genes with a median CPM > 1 (20,448 genes included in the analysis). The count matrix for genes with CPM > 1 was loaded into R v.4.2.2. We performed differential gene expression analysis with DESeq2 (ref. ^78^) v.1.38.3 using default parameters. Differential gene expression analysis was performed between samples from patients with AD and cognitively unimpaired controls. We set the threshold for differential expression at log_2_(fold-change) > 1 and false discovery rate (FDR)-corrected *P* value (*q* value) <0.05. Detailed descriptions of statistical analysis results can be found in Supplementary Table 9. DESeq2 utilizes Wald’s test for statistical comparisons.

### Differential isoform expression analysis

For differential isoform expression analysis, we used the transcript count matrix output by bambu. We performed differential isoform expression analysis only on transcripts with a median CPM > 1 coming from genes expressing two or more transcripts with median CPM > 1 (19,423 transcripts from 7,042 genes included in the analysis). This filtered count matrix was loaded into R v.4.2.2. We performed differential isoform expression analysis with DESeq2 v.1.38.3 using default parameters. Differential isoform expression analysis was performed using the same methods as the gene-level analysis, comparing samples from patients with AD and cognitively unimpaired controls, including the same significance thresholds (log_2_(fold-change) > 1) and FDR-corrected *P* < 0.05. Detailed descriptions of statistical analysis results can be found in Supplementary Table 10. DESeq2 utilizes Wald’s test for statistical comparisons.

### Figures and tables

Figures and tables were generated using customized R (v.4.2.2) scripts and customized Python (v.3.10.8) scripts. We used the following R libraries: tidyverse (v.1.3.2), EnhancedVolcano (v.1.18.0), DESeq2 (v.1.38.3) and ggtranscript^79^ (v.0.99.3). We used the following Python libraries: numpy (v.1.24.1), pandas (v.1.5.2), regex (v.2022.10.31), matplotlib (v.3.6.2), seaborn (v.0.12.2), matplotlib_venn (v.0.11.7), wordcloud (v.1.8.2.2), plotly (v.5.11.0) and notebook (v.6.5.2). See ‘Code availability’ for access to the customized scripts used to generate figures and tables.

### PCR primer design

We used the extended annotation output by bambu to create a reference transcriptome for primer design. This extended annotation contained information for all transcripts contained in Ensembl v.107 with the addition of all newly discovered transcripts by bambu (without applying a median CPM filter) and the ERCC spike-in transcripts. This annotation was converted into a transcriptome sequence fasta file using gffread (v.0.12.7) and the GRCh38 human reference genome. We used the online National Center for Biotechnology Information (NCBI) primer design tool (https://www.ncbi.nlm.nih.gov/tools/primer-blast) to design primers. We utilized default settings for the tool; however, we provided the transcriptome described above as the customized database to check for primer pair specificity. We moved forward with validation only when we could generate a primer pair specific to a single new high-confidence transcript. Detailed information about the primers—including primer sequence—used for gel electrophoresis PCR and RT–qPCR validations can be found in Supplementary Tables 4 and 5.

### PCR and gel electrophoresis validations

New isoform and gene validations were conducted using PCR and gel electrophoresis. For this purpose, 2 μg of RNA was transcribed into cDNA using the High-Capacity cDNA Reverse Transcription kit (AB Applied Biosystems, cat. no. 4368814) following the published protocol. The resulting cDNA was quantified using a nanodrop and its quality was assessed using the Agilent Fragment analyzer 5200 with the DNA (50 kb) kit (Agilent, DNF-467). Next, 500 ng of the cDNA was combined with primers specific to the newly identified isoforms and genes (Supplementary Table 4). The amplification was performed using Invitrogen Platinum II Taq Hot start DNA Polymerase (Invitrogen, cat. no. 14966-005) in the Applied Biosystem ProFlex PCR system. The specific primer sequences, annealing temperatures and number of PCR cycles are detailed in Supplementary Table 4. After the PCR amplification, the resulting products were analyzed on a 1% agarose Tris-acetate-EDTA gel containing 0.5 μg ml^−1^ of ethidium bromide. The gel was run for 30 min at 125 V and the amplified cDNA was visualized using an ultraviolet light source. Gels from PCR validation for each transcript can be found in Supplementary Figs. 5–26, 33 and 34. Some gels contain data from all 12 samples whereas others contain data only from 8 out of the 12 samples because we ran out of brain tissue for 4 of the samples.

### RT–qPCR validations

The RT–qPCR assays were performed using the QuantStudieo 5 Real-Time PCR System (Applied Biosystems). Amplifications were carried out in 25 μl of reaction solutions containing 12.5 μl of 2× PerfeCTa SYBR green SuperMix (Quantabio, cat. no. 95054-500), 1.0 μl of first-stranded cDNA, 1 μl of each specific primer (10 mM; Supplementary Table 5) and 9.0 μl of ultra-pure, nuclease-free water. RT–qPCR conditions involved an initial hold stage: 50 °C for 2 min followed by 95 °C for 3 min with a ramp of 1.6 °C s^−1^ followed by PCR stage of 95 °C for 15 s and 60 °C for 60 s for a total of 50 cycles. MIQE guidelines from ref. ^30^ suggest *C*_*t*_ < 40 as a cutoff for RT–qPCR validation, but we used a more stringent cutoff of *C*_*t*_ < 35 to be conservative. This means that we considered a new RNA isoform to be validated by RT–qPCR only if the mean *C*_t_ value for our samples was <35. We attempted to validate new RNA isoforms only through RT–qPCR if they first failed to be validated through standard PCR and gel electrophoresis. We did this because RT–qPCR is a more sensitive method, allowing us to validate RNA isoforms that are less abundant or that are harder to amplify through PCR. We performed RT–qPCR only using 8 of the 12 samples included in the present study because we ran out of brain tissue for 4 of the samples.

In addition, we performed quantification of new and known RNA isoforms from the following genes: *SLC26A1*, *MT-RNR2* and *MAOB* (Supplementary Tables 6 and 7). We followed recommendations in ref. ^80^ and used the *CYC1* as the gene for *C*_*t*_ value normalization in our human postmortem brain samples. To allow for comparison between different isoforms from the same gene, we used 2^−Δ*Ct*^ as the expression estimate instead of the more common 2^−ΔΔ*Ct*^ expression estimate. This is because the 2^−ΔΔ*Ct*^ expression estimate is optimized for comparisons between samples within the same gene/isoform, but does not work well for comparison between different genes/isoforms. On the other hand, the 2^−Δ*Ct*^ expression estimate allows for comparison between different genes/isoforms. RNA isoform relative abundance for RT–qPCR and long-read RNA-seq was calculated as follows:

$$\begin{array}{l}{{\mathrm{Relative}}}\,{{\mathrm{abundance}}}=\frac{{{\mathrm{Expression}}}\,{{\mathrm{estimate}}}\,{{\mathrm{for}}}\,{\mathrm{a}}\,{{\mathrm{given}}}\,{{\mathrm{RNA}}}\,{{\mathrm{isoform}}}}{\sum ({{\mathrm{Expression}}}\,{{\mathrm{estimates}}}\,{{\mathrm{for}}}\,{{\mathrm{RNA}}}\,{{\mathrm{isoforms}}}\,{{\mathrm{from}}}\,{{\mathrm{the}}}\,{{\mathrm{given}}}\,{{\mathrm{gene}}})}\times 100.\end{array}$$

### Proteomics analysis

We utilized publicly available tandem MS data from round 2 of the ROSMAP brain proteomics study, previously analyzed in refs. ^22^ and ^23^. We also utilized publicly available deep tandem MS data from six human cell lines, processed with six different proteases and three tandem MS fragmentation methods, previously analyzed in ref. ^24^. This cell-line dataset represents one of the largest human proteomes with the highest sequence coverage ever reported as of 2023. We started the analysis by creating a protein database containing the predicted protein sequence from all three reading frames for the 700 new high-confidence RNA isoforms that we discovered, totaling 2,100 protein sequences. We translated each high-confidence RNA isoform in three reading frames using pypGATK^81^ v.0.0.23. We also included the protein sequences for known protein-coding transcripts that came from genes represented in the 700 new high-confidence RNA isoforms and had a median CPM > 1 in our RNA-seq data. We used this reference protein fasta file to process the brain and cell-line proteomics data separately using FragPipe^82–88^ v.20.0—a Java-based graphic user interface that facilitates the analysis of MS-based proteomics data by providing a suite of computational tools. Detailed parameters used for running FragPipe can be found on GitHub and Zenodo (‘Code availability’ and ‘Data availability’).

MS suffers from a similar issue as short-read RNA-seq, being able to detect only relatively short peptides that do not cover the entire length of most proteins. This makes it challenging to accurately detect RNA isoforms from the same gene. To avoid false discoveries, we took measures to ensure that we would consider an RNA isoform to be validated at the protein level only if it had peptide hits that are unique to it (that is, not contained in other known human proteins). We started by taking the FragPipe output and keeping only peptide hits that mapped to only one of the proteins in the database. We then ran the sequence from those peptides against the database we provided to FragPipe to confirm that they were truly unique. Surprisingly, a small percentage of peptide hits that FragPipe reported as unique were contained in two or more proteins in our database; these hits were excluded from downstream analysis. We then summed the number of unique peptide spectral counts for every protein coming from a new high-confidence RNA isoform. We filtered out any proteins with fewer than six spectral counts. We took the peptide hits for proteins that had more than five spectral counts and used the online protein–protein NCB blast tool (blastp: https://blast.ncbi.nlm.nih.gov/Blast.cgi?PAGE=Proteins)^89^ to search it against the human RefSeq protein database. We used loose thresholds for our blast search to ensure that even short peptide matches would be reported. A detailed description of the blast search parameters can be found on Zenodo. Spectral counts coming from peptides that had a blast match with 100% query coverage and 100% identity to a known human protein were removed from downstream analysis. We took the remaining spectral counts after the blast search filter and summed them by protein ID. Proteins from high-confidence RNA isoforms that had more than five spectral counts after a blast search filter were considered to be validated at the protein level. This process was repeated to separately analyze the brain MS data and the cell-line MS data.

### Rigor and reproducibility

The present study was done under the ethics oversight of the University of Kentucky Institutional Review Board. Read preprocessing, alignment, filtering, transcriptome quantification and discovery, and quality control steps for Nanopore and Illumina data were implemented using customized NextFlow pipelines. NextFlow enables scalable and reproducible scientific workflows using software containers^90^. We used NextFlow v.23.04.1.5866. Singularity containers were used for most of the analysis in the present study, except for website creation and proteomics analysis owing to feasibility issues. Singularity containers enable the creation and employment of containers that package up pieces of software in a way that is portable and reproducible^91^. We used Singularity v.3.8.0-1.el8. Instructions on how to access the singularity containers that can be found in the GitHub repository for this project. Any changes to standard manufacturer protocols have been detailed in Methods. All code used for analysis in this article is publicly available on GitHub. All raw data, output from long-read RNA-seq and proteomics pipelines, references and annotations are publicly available. Long-read RNA-seq results from this article can be easily visualized through this web application: https://ebbertlab.com/brain_rna_isoform_seq.html.

### Reporting summary

Further information on research design is available in the Nature Portfolio Reporting Summary linked to this article.

## Online content

Any methods, additional references, Nature Portfolio reporting summaries, source data, extended data, supplementary information, acknowledgements, peer review information; details of author contributions and competing interests; and statements of data and code availability are available at 10.1038/s41587-024-02245-9.

## Supplementary information

Supplementary Information Supplementary Figs. 1–61 and Lexogen SPLIT RNA extraction kit user guide.

Reporting Summary

Supplementary Tables 1–10 This Excel workbook contains 10 Excel sheets, Supplementary Tables 1–10. Supplementary Table 1: Sample characteristics and sequencing information. Supplementary Table 2: Overlap between high-confidence transcripts discovered in our study with transcripts discovered in refs. ^19^ and ^20^. Supplementary Table 3: Summary statistics for overlap between high-confidence transcripts discovered in our study with transcripts discovered in refs. ^19^ and ^20^. Supplementary Table 4: Information for gel electrophoresis PCR experiments. Supplementary Table 5: Information for three batches of RT–qPCR experiments. Supplementary Table 6: Quantification for new and known RNA isoforms from *MAOB*, *SLC26A1* and *MT-RNR2* using RT–qPCR. Supplementary Table 7: Summary statistics from quantification for new and known RNA isoforms from *MAOB*, *SLC26A1* and *MT-RNR2* using RT–qPCR. Supplementary Table 8: Number of counts from ROSMAP and GTEx data that were uniquely aligned to new transcripts discovered in our study. Supplementary Table 9: Gene-level differential expression results between cases of AD (*n* = 6) and cognitively unimpaired controls (*n* = 6). We used the DESeq2 R package with two-sided Wald’s test for statistical comparisons and Benjamini–Hochberg correction for multiple comparisons in this differential expression analysis. Raw *P* values are in the ‘p value’ column and Benjamini–Hochberg FDR-adjusted *P* values are in the ‘p_adj_’ column. Supplementary Table 10: Transcript-level differential expression results between cases with AD (*n* = 6) and cognitively unimpaired controls (*n* = 6). We used the DESeq2 R package with two-sided Wald’s test for statistical comparisons and Benjamini–Hochberg correction for multiple comparisons in this differential expression analysis. Raw *P* values are in the ‘pvalue’ column and Benjamini–Hochberg FDR-adjusted *P* values are in the ‘p_adj_’ column.

## Data Availability

Raw long-read RNA-seq data generated and utilized in the present study are publicly available in Synapse^92^: https://www.synapse.org/#!Synapse:syn52047893. Raw long-read RNA-seq data generated and utilized in the present study are also publicly available in NIH Sequence Read Archive (SRA) (accession no. SRP456327)^93^ https://trace.ncbi.nlm.nih.gov/Traces/?view=study&acc=SRP456327. Output from long-read RNA-seq and proteomics pipelines, reference files and annotations are publicly available at^94^ 10.5281/zenodo.8180677. Long-read RNA-seq results from this article can be easily visualized through this web application: https://ebbertlab.com/brain_rna_isoform_seq.html. Raw cell-line deep proteomics data utilized in this article are publicly available at https://proteomecentral.proteomexchange.org/cgi/GetDataset?ID=PXD024364. Raw brain proteomics data from round 2 of the ROSMAP TMT study are publicly available at https://www.synapse.org/#!Synapse:syn17015098. GTEx long-read RNA-seq data used for validation of our study results are available at https://anvil.terra.bio/#workspaces/anvil-datastorage/AnVIL_GTEx_V9_hg38. ROSMAP short-read RNA-seq data used for validation of our study results are available at https://www.synapse.org/#!Synapse:syn21589959. CHM13 reference genome sequence can be found at https://s3-us-west-2.amazonaws.com/human-pangenomics/T2T/CHM13/assemblies/analysis_set/chm13v2.0.fa.gz. CHM13 reference GFF3 annotation can be found at https://s3-us-west-2.amazonaws.com/human-pangenomics/T2T/CHM13/assemblies/annotation/chm13.draft_v2.0.gene_annotation.gff3. The transcript annotation from Glinos et al.^19^ was retrieved from https://storage.googleapis.com/gtex_analysis_v9/long_read_data/flair_filter_transcripts.gtf.gz. The transcript annotation from Leung et al.^20^ was retrieved from https://zenodo.org/record/7611814/preview/Cupcake_collapse.zip#tree_item12/HumanCTX.collapsed.gff.

## Code availability

All code used in the manuscript is publicly available at https://github.com/UK-SBCoA-EbbertLab/brain_cDNA_discovery (ref. ^95^).
