# Supplementary material for: Mapping medically relevant RNA isoform diversity in the aged human frontal cortex with deep long-read RNA-seq
Source: Nat Biotechnol. 2024 May 22;43(4):635–46. doi: 10.1038/s41587-024-02245-9 (PMC11863200; doi:10.1038/s41587-024-02245-9)
Supplement: Supplementary file 1 — Supplementary Figs. 1–61 and Lexogen SPLIT RNA extraction kit user guide. [file 41587_2024_2245_MOESM1_ESM.pdf]

nature biotechnology

Supplementary information

https://doi.org/10.1038/s41587-024-02245-9

Mapping medically relevant RNA isoform diversity in the aged human frontal cortex with deep long-read RNA-seq

In the format provided by the authors and unedited

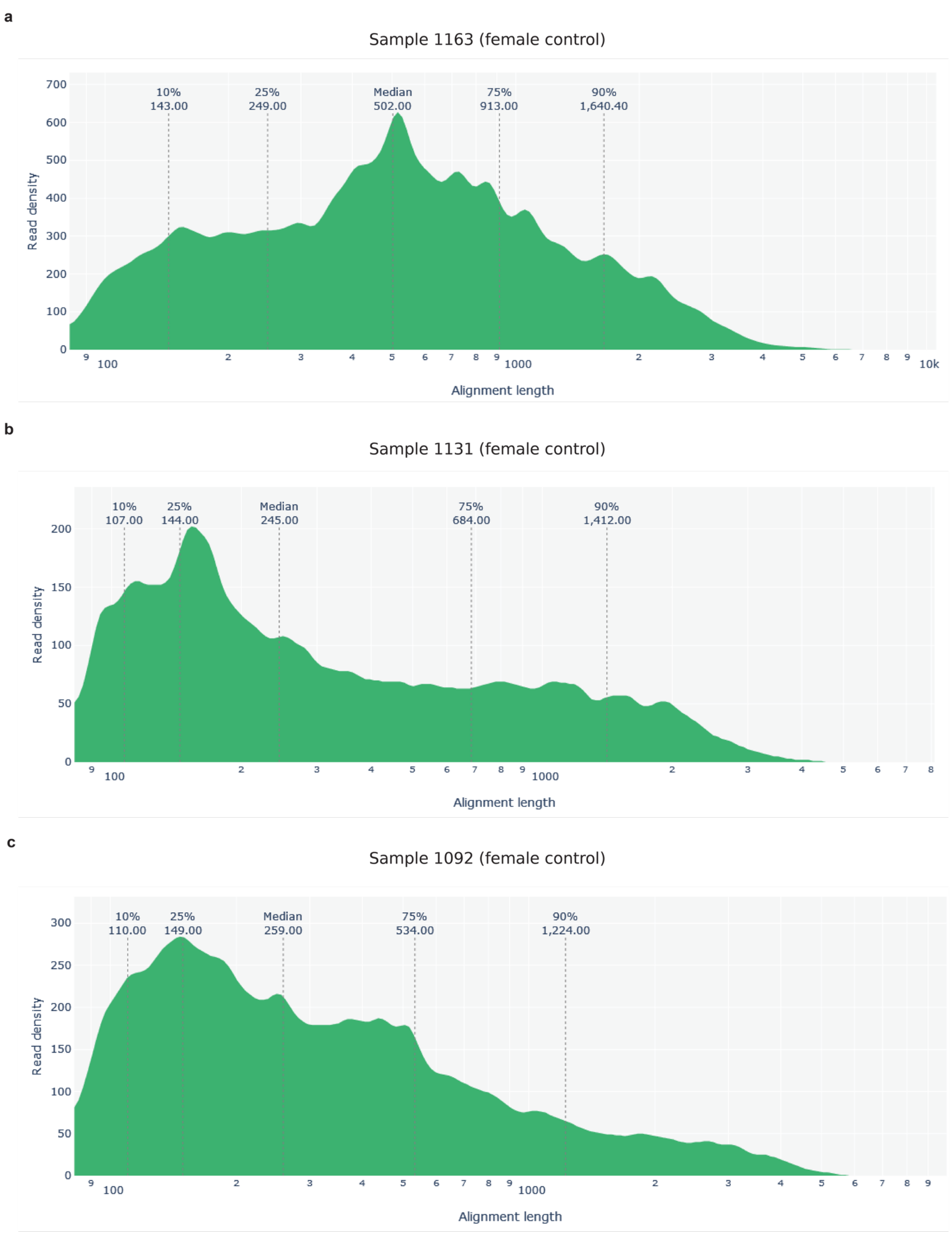

Supplementary Figure 1: Aligned read length distribution for the three female control brain samples.

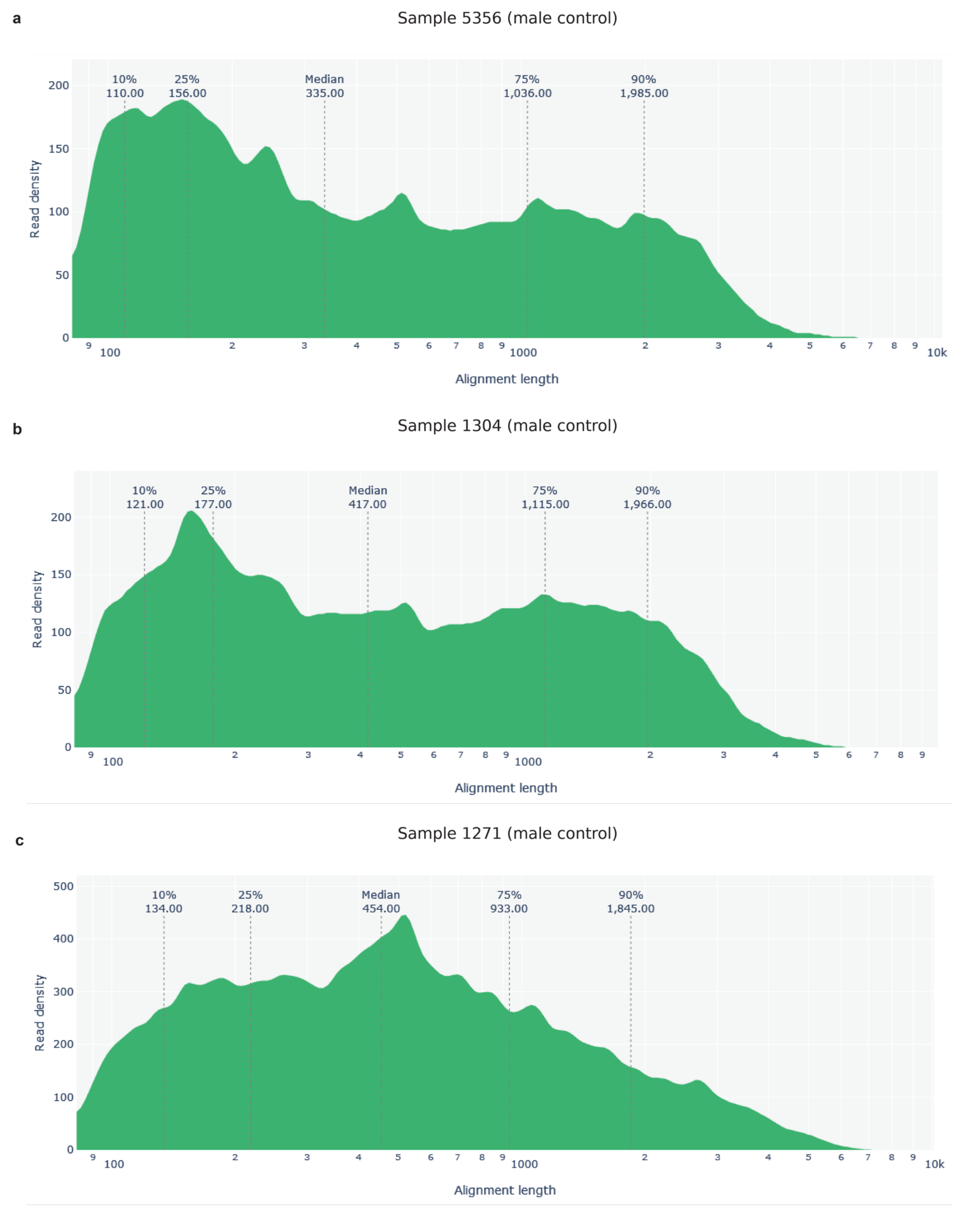

Supplementary Figure 2: Aligned read length distribution for the three male control brain samples.

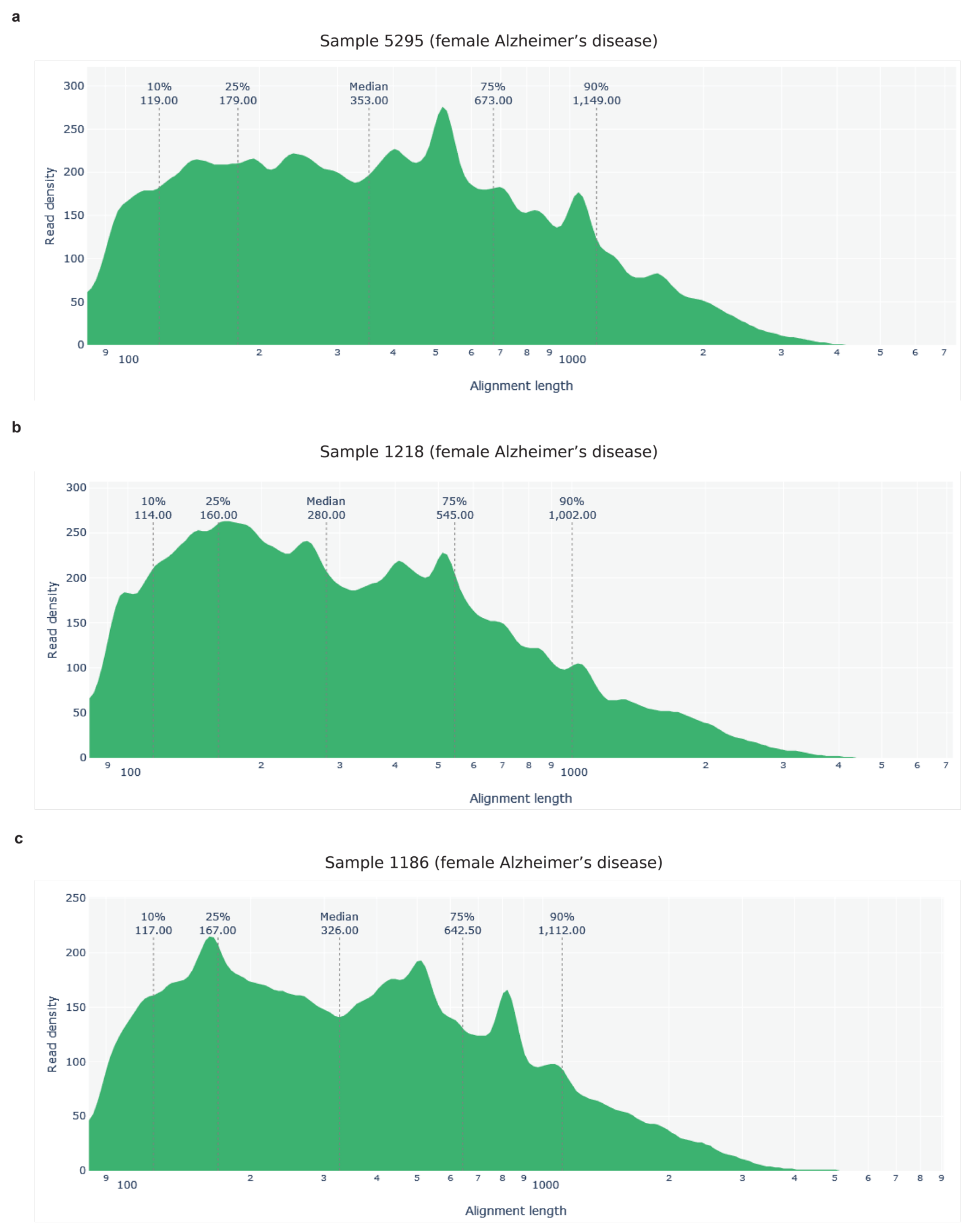

Supplementary Figure 3: Aligned read length distribution for the three female Alzheimer’s disease brain samples.

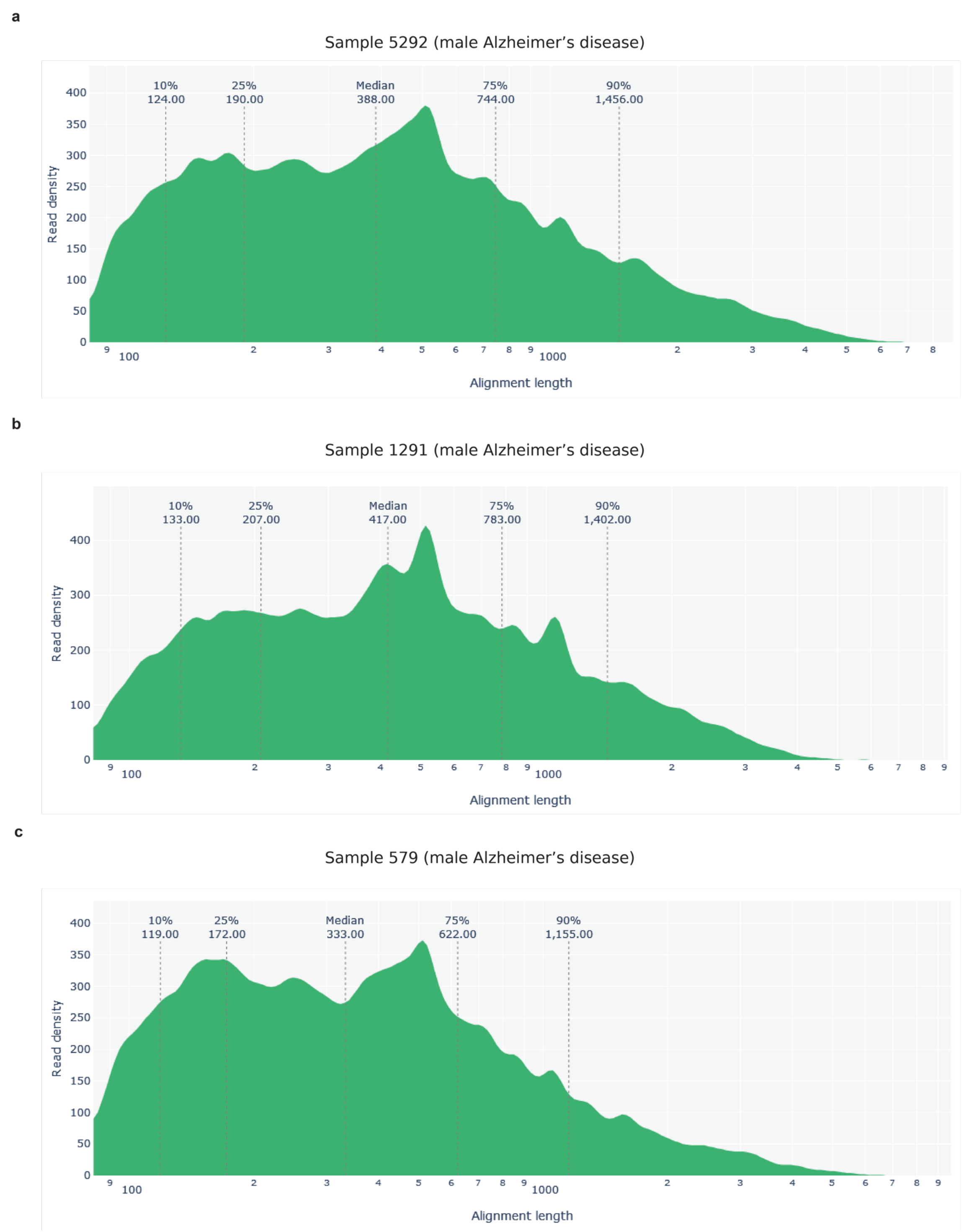

Supplementary Figure 4: Aligned read length distribution for the three male Alzheimer’s disease brain samples.

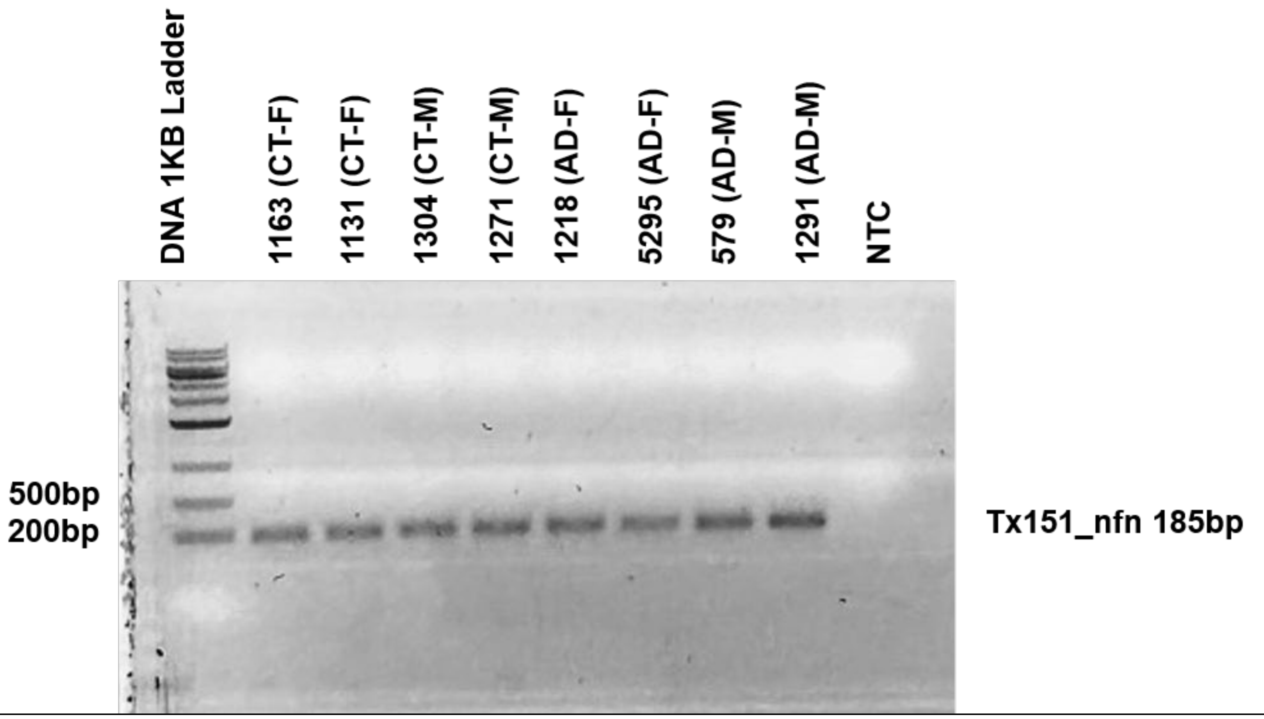

Supplementary Figure 5: PCR validation for new high confidence transcript Tx151 (New gene body*).*

Number above the lanes is the sample id for 8 out of the 12 samples used in this study. The other 4 samples were not included because we ran out of tissue left for generating new cDNA. Labels on the right side of the figure indicate transcript id for the new high-confidence RNA isoform being validated and the expected product length from the PCR primers used to amplify the RNA isoform. Tx151 is a new RNA isoform from a newly discovered gene body and was succesfully validated in this gel. This gel electrophoresis PCR experiment was independently performed one time with similar results.

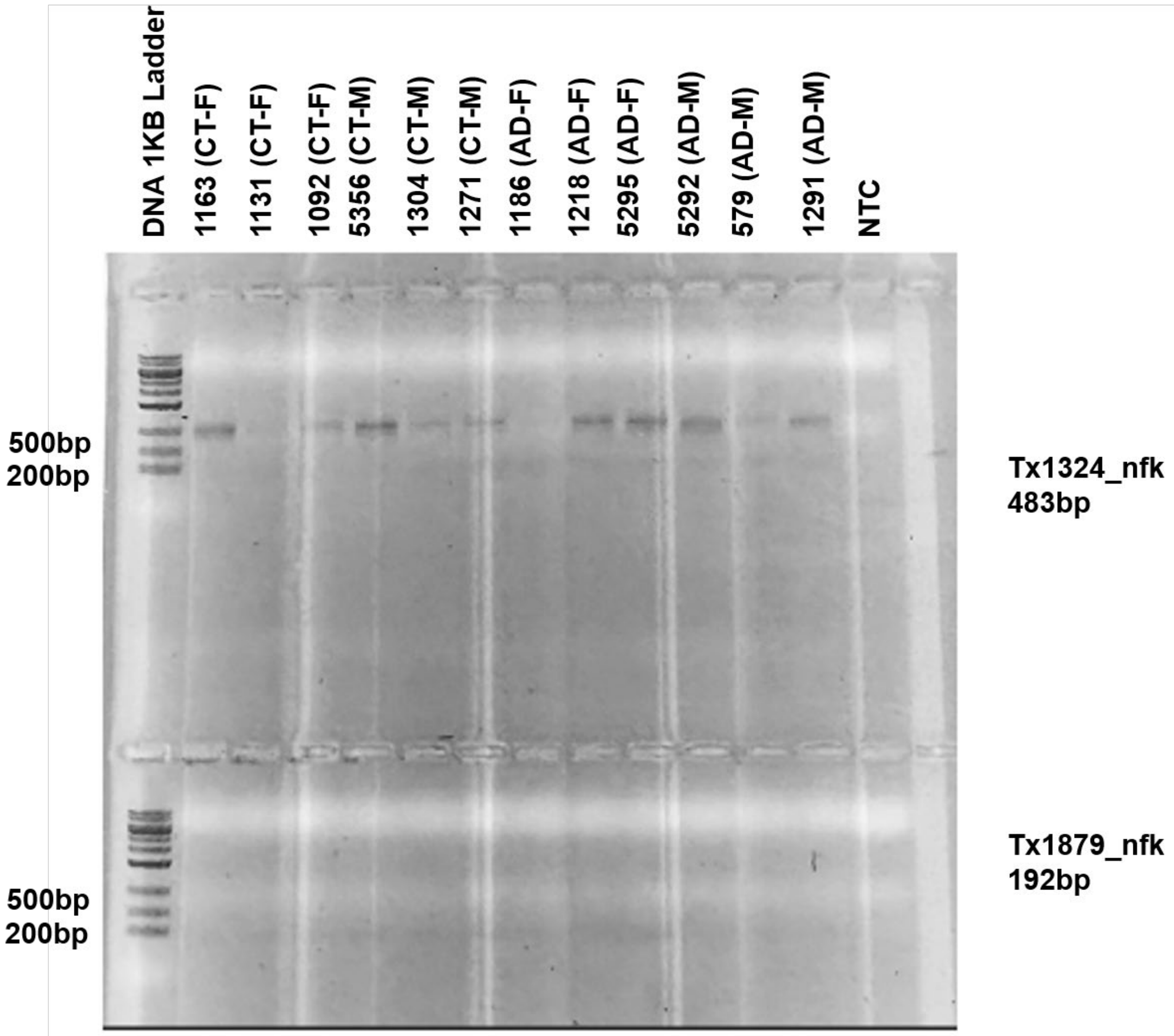

Supplementary Figure 6: PCR validation for new high confidence transcripts Tx1324 (*NELFA)* and Tx1879 (*MAOB*).
Number above the lanes is the sample id for 8 out of the 12 samples used in this study. The other 4 samples were not included because we ran out of tissue left for generating new cDNA. Labels on the right side of the figure indicate transcript id for the new high-confidence RNA isoform being validated and the expected product length from the PCR primers used to amplify the RNA isoform. Tx1324 is a new RNA isoform from the *NELFA* gene and was succesfully validated in this gel. Tx1879 is a new RNA isoform for the *MAOB* gene and was not validated in this gel, but was validated with a different primer pair in **Supplementary Figure 16**. This gel electrophoresis PCR experiment was independently performed one time with similar results.

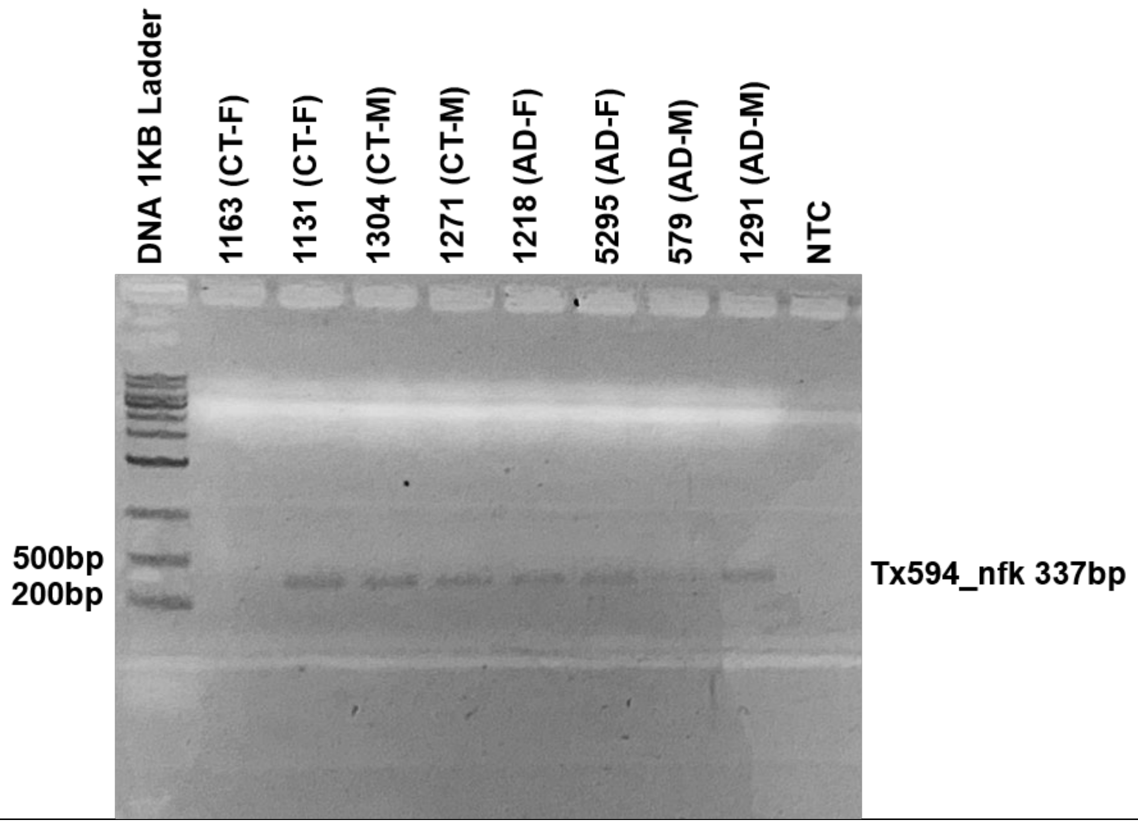

Supplementary Figure 7: PCR validation for new high confidence transcript Tx594 (*MTHFS*).
Number above the lanes is the sample id for 8 out of the 12 samples used in this study. The other 4 samples were not included because we ran out of tissue left for generating new cDNA. Labels on the right side of the figure indicate transcript id for the new high-confidence RNA isoform being validated and the expected product length from the PCR primers used to amplify the RNA isoform. Tx594 is a new RNA isoform from the MTHFS gene and was succesfully validated in this gel. This gel electrophoresis PCR experiment was independently performed one time with similar results.

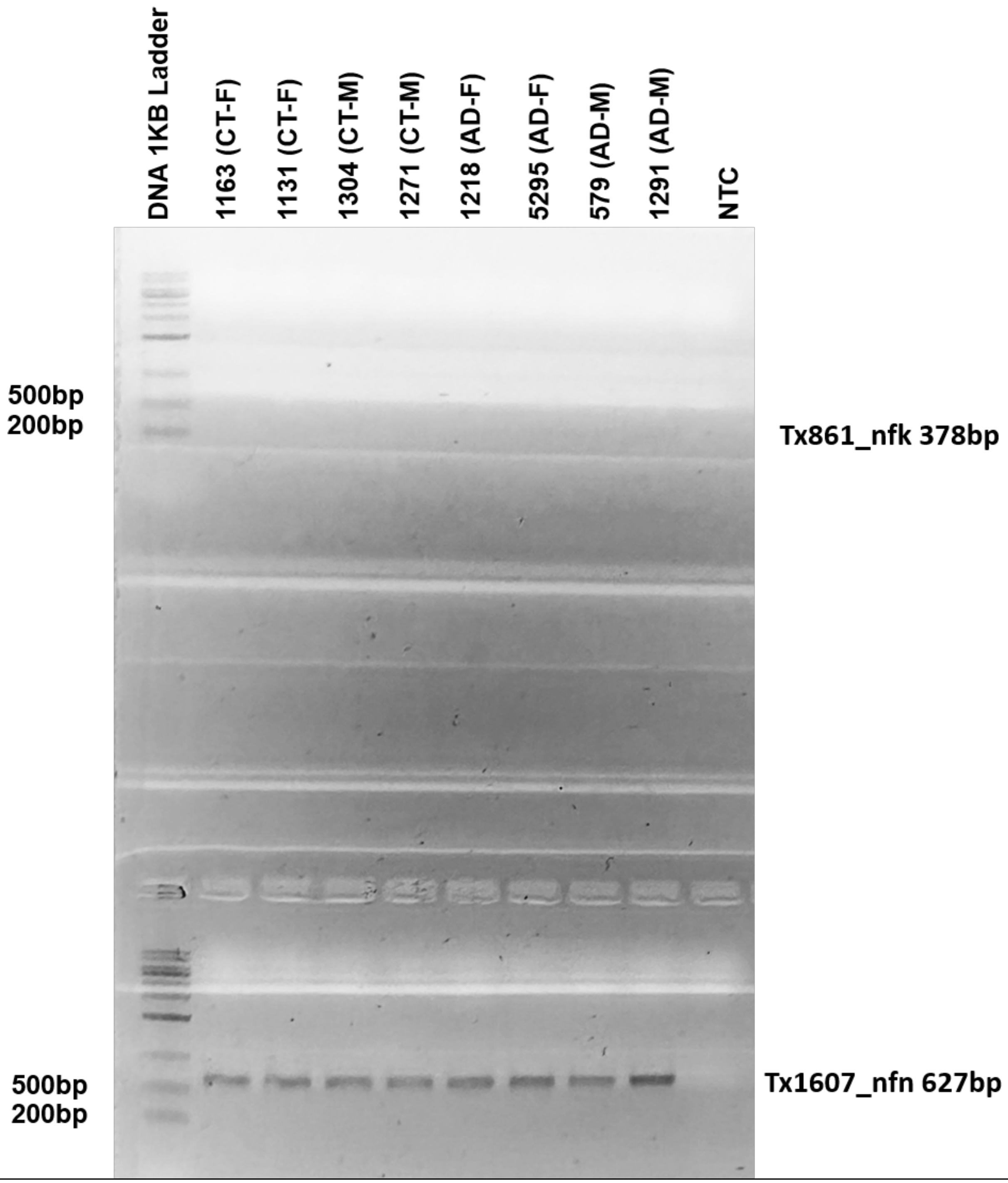

Supplementary Figure 8: PCR validation for new high confidence transcripts Tx861 (*PSENEN)* and Tx1607 (New gene body).
Number above the lanes is the sample id for 8 out of the 12 samples used in this study. The other 4 samples were not included because we ran out of tissue left for generating new cDNA. Labels on the right side of the figure indicate transcript id for the new high-confidence RNA isoform being validated and the expected product length from the PCR primers used to amplify the RNA isoform. Tx861 is a new RNA isoform from the *PSENEN* gene and was not validated in this gel, however it was validated through RT-qPCR (**Supplementary Table 5**). Tx2603 is a new RNA isoform ffrom a newly discovered gene body and was succesfully validated in this gel. This gel electrophoresis PCR experiment was independently performed one time with similar results.

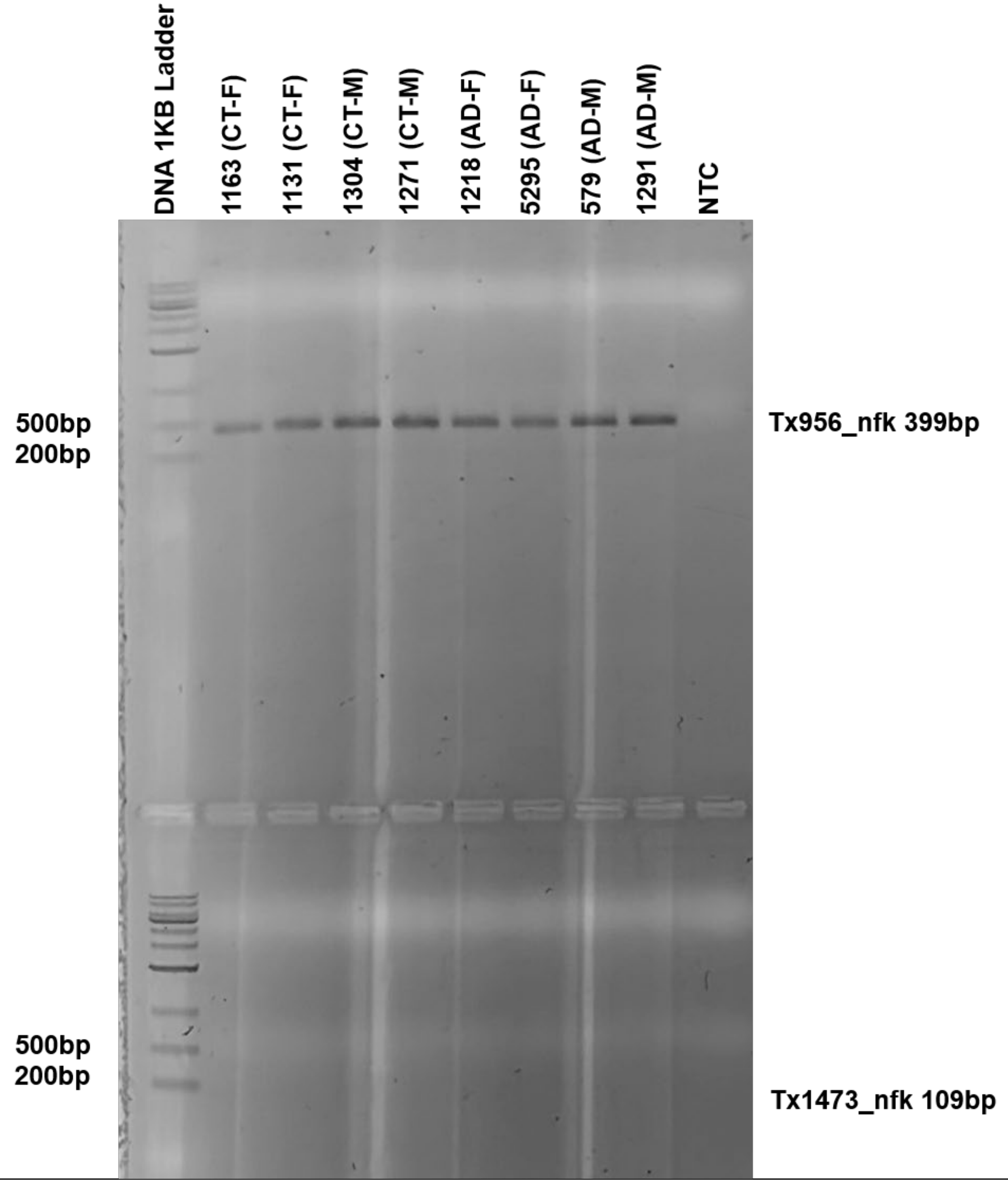

Supplementary Figure 9: PCR validation for new high confidence transcripts Tx956 (*CAMKMT)* and Tx1473 (*CPLX2*).
Number above the lanes is the sample id for 8 out of the 12 samples used in this study. The other 4 samples were not included because we ran out of tissue left for generating new cDNA. Labels on the right side of the figure indicate transcript id for the new high-confidence RNA isoform being validated and the expected product length from the PCR primers used to amplify the RNA isoform. Tx956 is a new RNA isoform from the *CAMKMT* gene and was succesfully validated in this gel. Tx1473 is a new RNA isoform from the *CPLX2* gene and was not validated in this gel and was also not validated through RT-qPCR (**Supplementary Table 5**). This gel electrophoresis PCR experiment was independently performed one time with similar results.

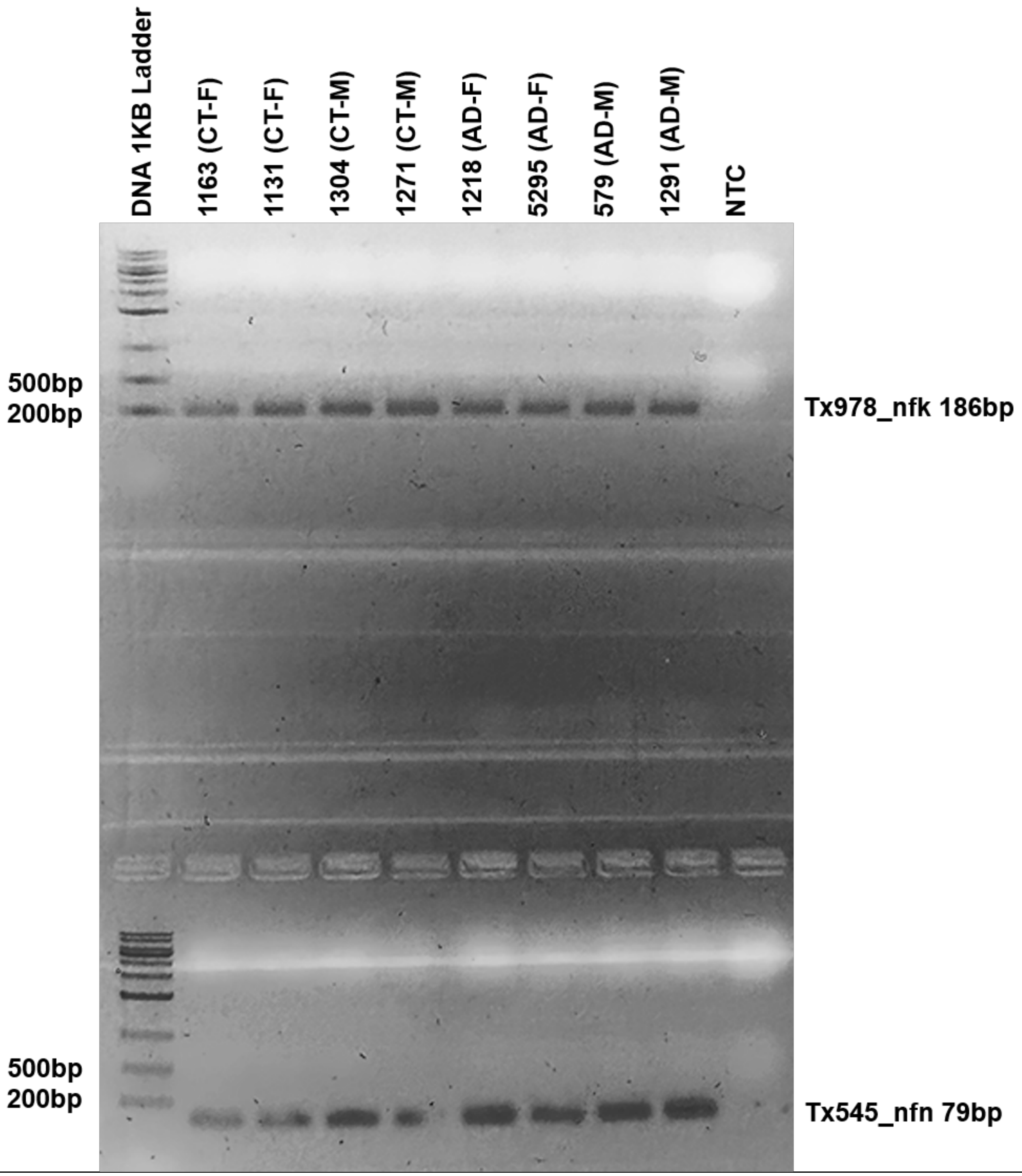

Supplementary Figure 10: PCR validation for new high confidence transcripts Tx978 (*DGUOK)* and Tx545 (New gene body).

Number above the lanes is the sample id for 8 out of the 12 samples used in this study. The other 4 samples were not included because we ran out of tissue left for generating new cDNA. Labels on the right side of the figure indicate transcript id for the new high-confidence RNA isoform being validated and the expected product length from the PCR primers used to amplify the RNA isoform. Tx978 is a new RNA isoform from the *DGUOK* gene and was succesfully validated in this gel. Tx545 is a new RNA isoform from a newly discovered gene body and was succesfully validated in this gel. This gel electrophoresis PCR experiment was independently performed one time with similar results.

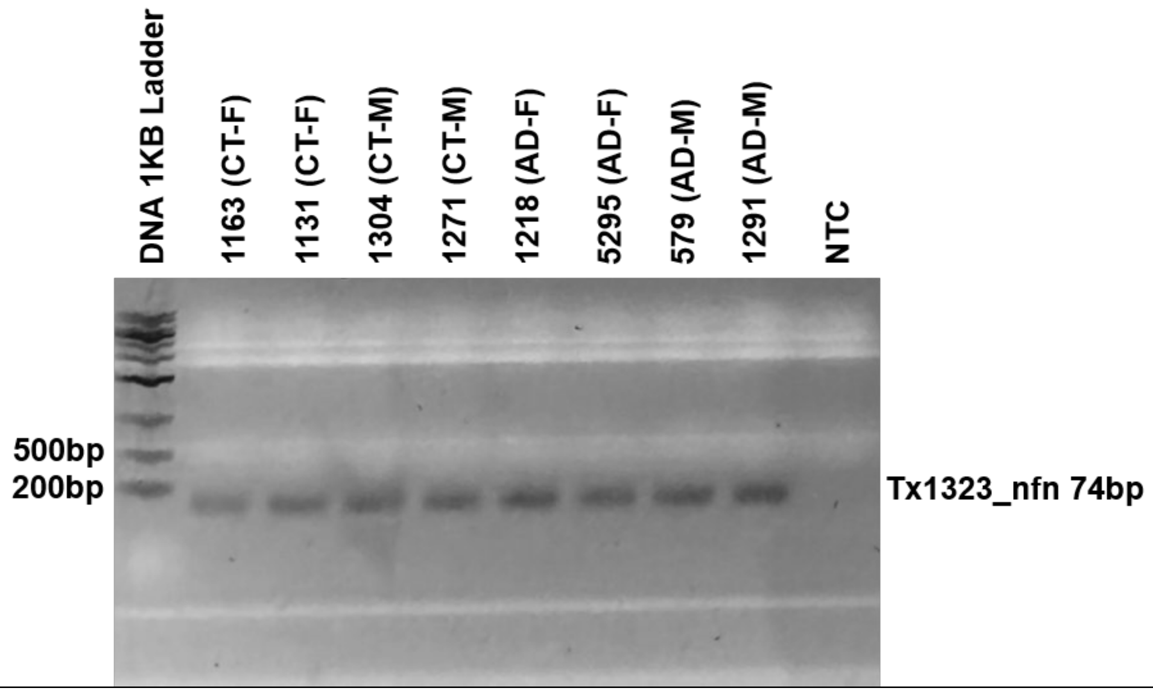

Supplementary Figure 11: PCR validation for new high confidence transcript Tx1323 (New gene body*).*

Number above the lanes is the sample id for 8 out of the 12 samples used in this study. The other 4 samples were not included because we ran out of tissue left for generating new cDNA. Labels on the right side of the figure indicate transcript id for the new high-confidence RNA isoform being validated and the expected product length from the PCR primers used to amplify the RNA isoform. Tx1323 is a new RNA isoform from a newly discovered gene body and was succesfully validated in this gel. This gel electrophoresis PCR experiment was independently performed one time with similar results.

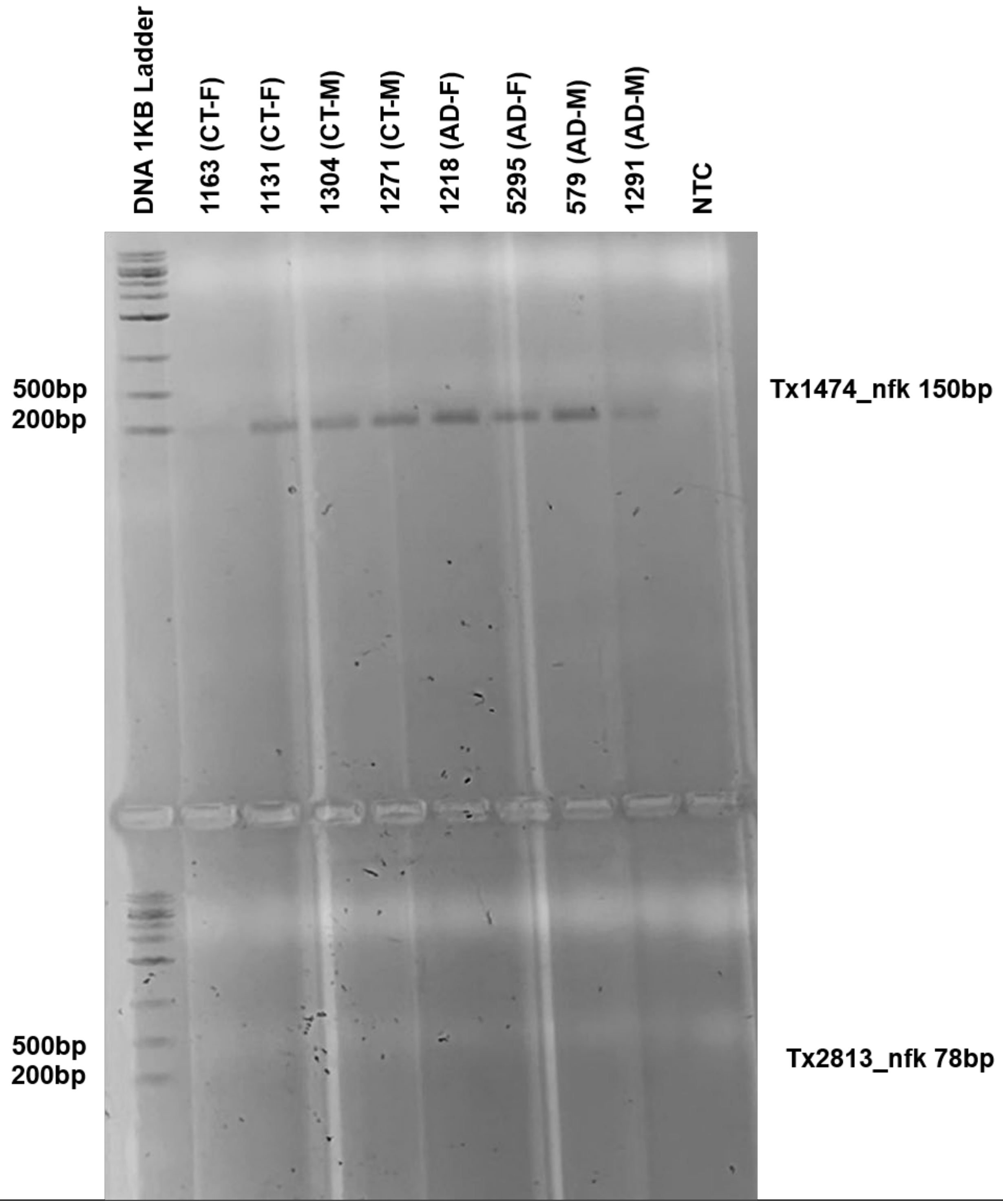

Supplementary Figure 12: PCR validation for new high confidence transcripts Tx1474 (*CPLX2)* and Tx2813 (*PSENEN*).
Number above the lanes is the sample id for 8 out of the 12 samples used in this study. The other 4 samples were not included because we ran out of tissue left for generating new cDNA. Labels on the right side of the figure indicate transcript id for the new high-confidence RNA isoform being validated and the expected product length from the PCR primers used to amplify the RNA isoform. Tx1474 is a new RNA isoform from the *CPLX2* gene and was succesfully validated in this gel. Tx2813 is a new RNA isoform from the *PSENEN* gene and was succesfully validated in this gel, however it was validated through RT-qPCR (**Supplementary Table 5**). This gel electrophoresis PCR experiment was independently performed one time with similar results.

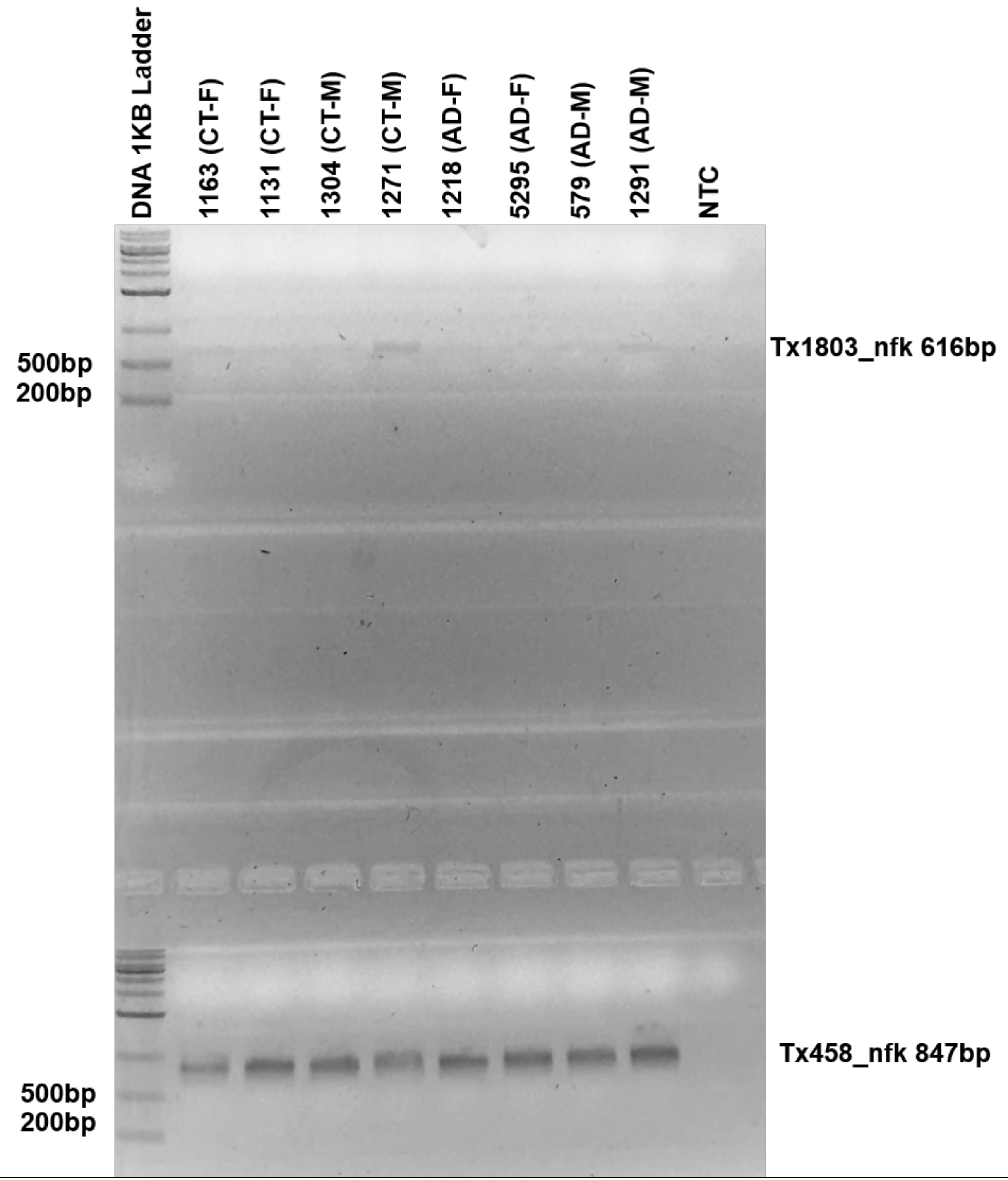

Supplementary Figure 13: PCR validation for new high confidence transcripts Tx1803 (*TMOD1*) and Tx458 (*NAA16*).
Number above the lanes is the sample id for 8 out of the 12 samples used in this study. The other 4 samples were not included because we ran out of tissue left for generating new cDNA. Labels on the right side of the figure indicate transcript id for the new high-confidence RNA isoform being validated and the expected product length from the PCR primers used to amplify the RNA isoform. Tx1803 is a new RNA isoform from the *TMOD1* gene and was not validated in this gel, however it was validated through RT-qPCR (**Supplementary Table 5**). Tx458 is a new RNA isoform from the *NAA16* gene and was succesfully validated in this gel. This gel electrophoresis PCR experiment was independently performed one time with similar results.

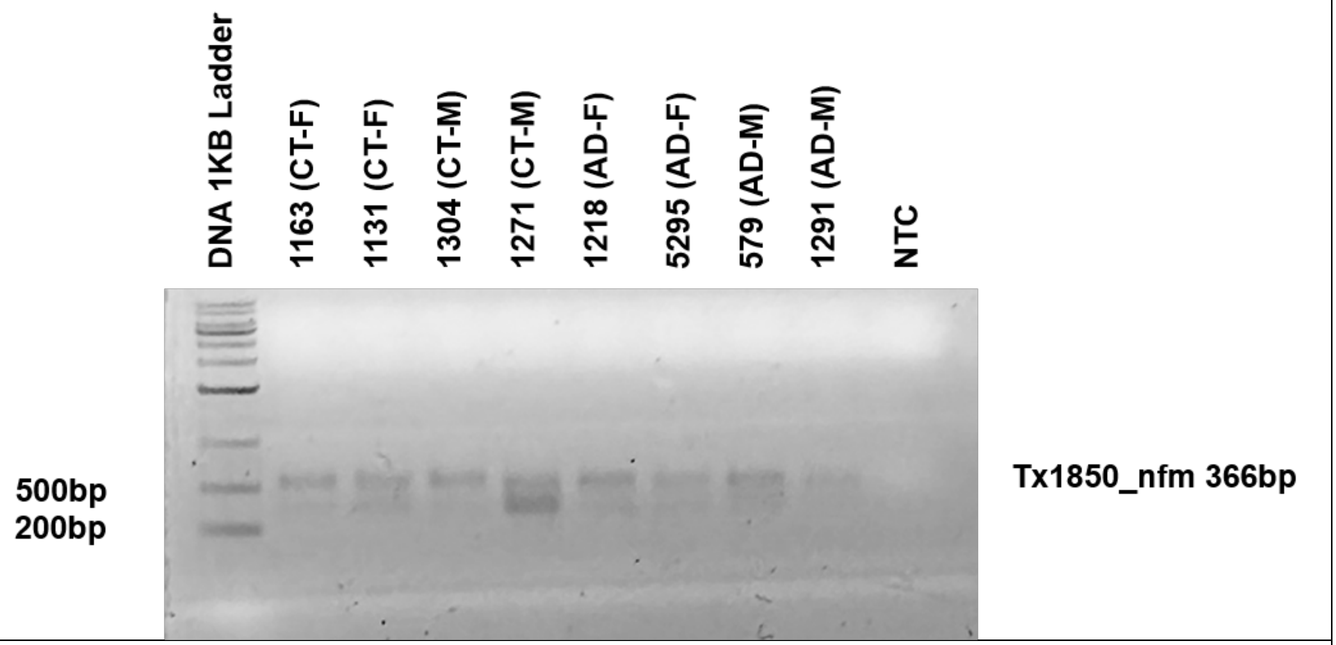

Supplementary Figure 14: PCR validation for new high confidence transcript Tx1850 (New spliced mitochondrial isoform).
Number above the lanes is the sample id for 8 out of the 12 samples used in this study. The other 4 samples were not included because we ran out of tissue left for generating new cDNA. Labels on the right side of the figure indicate transcript id for the new high-confidence RNA isoform being validated and the expected product length from the PCR primers used to amplify the RNA isoform. Tx1850 is a new spliced RNA isoform from mitochondria and was not succesfully validated due to double bands shown in gel that indicate promiscous primer binding. Because of promiscous primer binding we did not attempt to validate it through RT-qPCR. This gel electrophoresis PCR experiment was independently performed two timed with similar results.

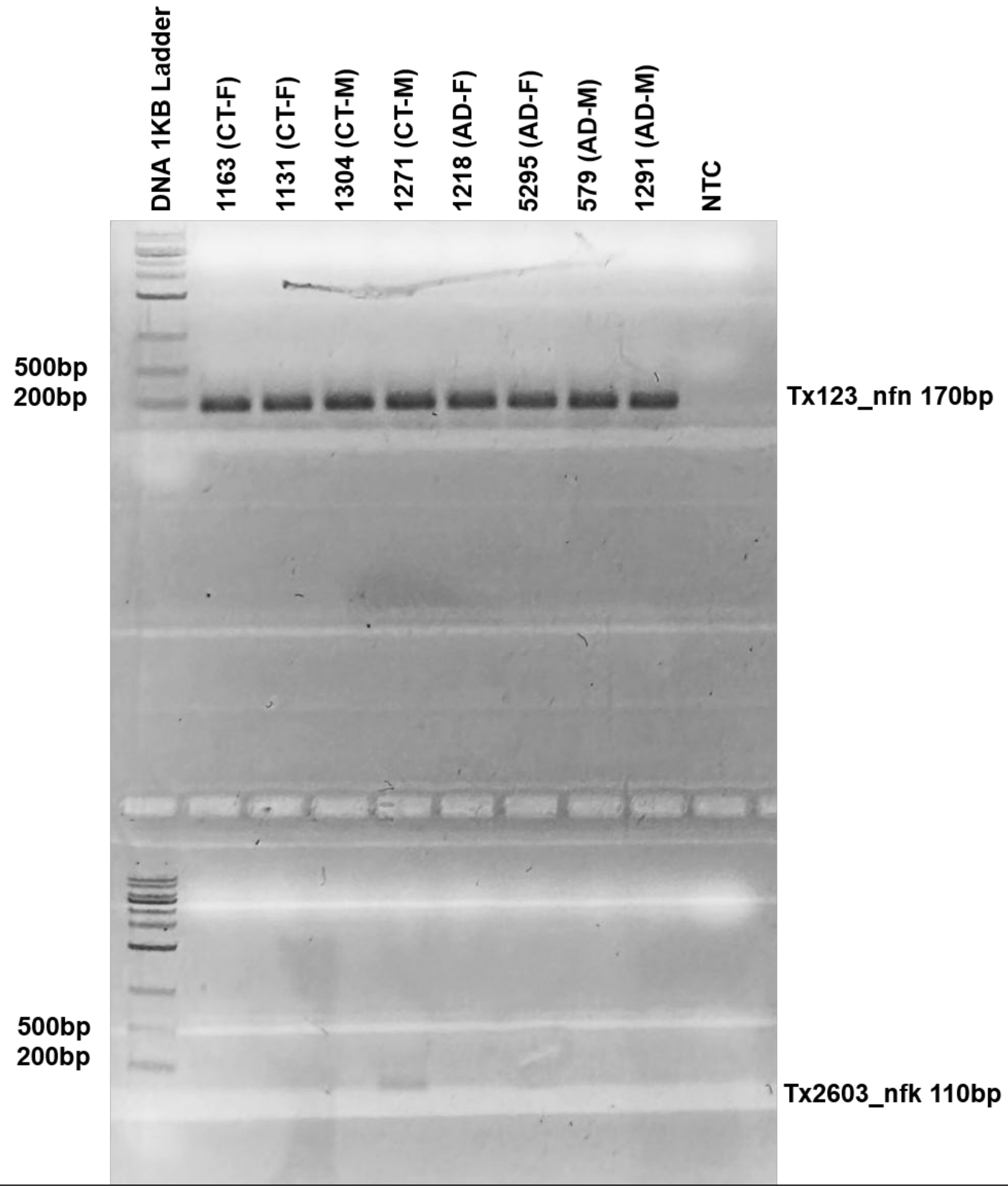

Supplementary Figure 15: PCR validation for new high confidence transcripts Tx123 (New gene body*)* and Tx2603 (*WDR4*).

Number above the lanes is the sample id for 8 out of the 12 samples used in this study. The other 4 samples were not included because we ran out of tissue left for generating new cDNA. Labels on the right side of the figure indicate transcript id for the new high-confidence RNA isoform being validated and the expected product length from the PCR primers used to amplify the RNA isoform. Tx123 is a new RNA isoform from a newly discovered gene body and was succesfully validated in this gel. Tx2603 is a new RNA isoform for the *WRD4* gene and was not validated in this gel, however it was validated through RT-qPCR (**Supplementary Table 5**). This gel electrophoresis PCR experiment was independently performed one time with similar results.

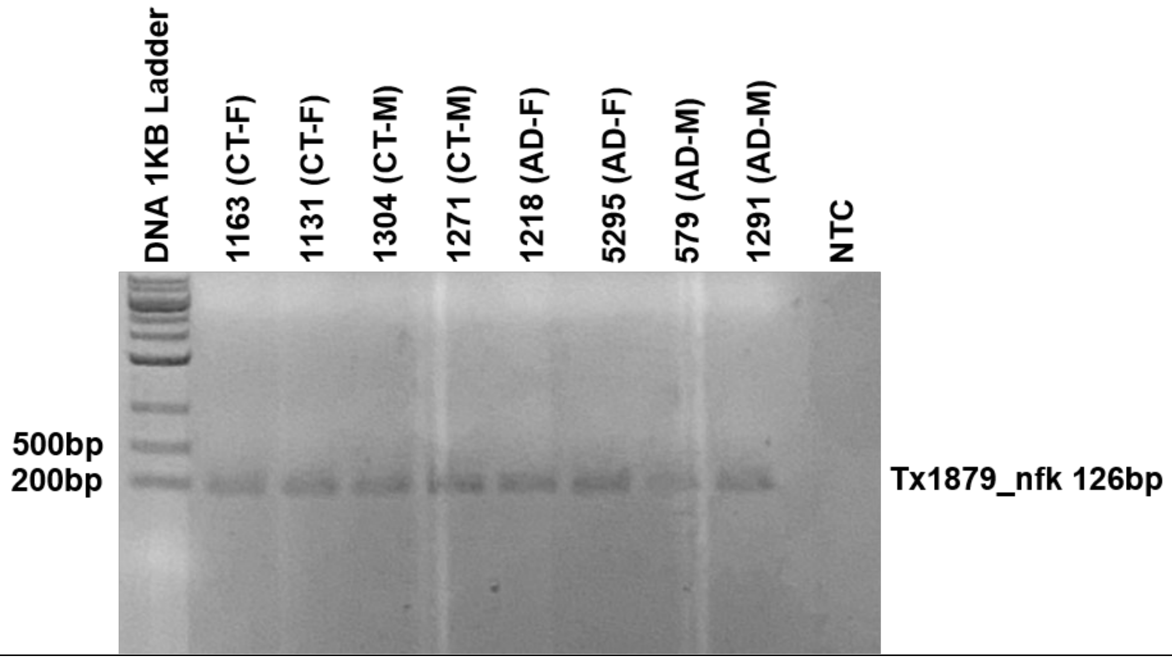

Supplementary Figure 16: PCR validation for new high confidence transcript Tx1879 (*MAOB*).

Number above the lanes is the sample id for 8 out of the 12 samples used in this study. The other 4 samples were not included because we ran out of tissue left for generating new cDNA. Labels on the right side of the figure indicate transcript id for the new high-confidence RNA isoform being validated and the expected product length from the PCR primers used to amplify the RNA isoform. Tx1879 is a new RNA isoform from the *MAOB* gene and was succesfully validated in this gel. This gel electrophoresis PCR experiment was independently performed one time with similar results.

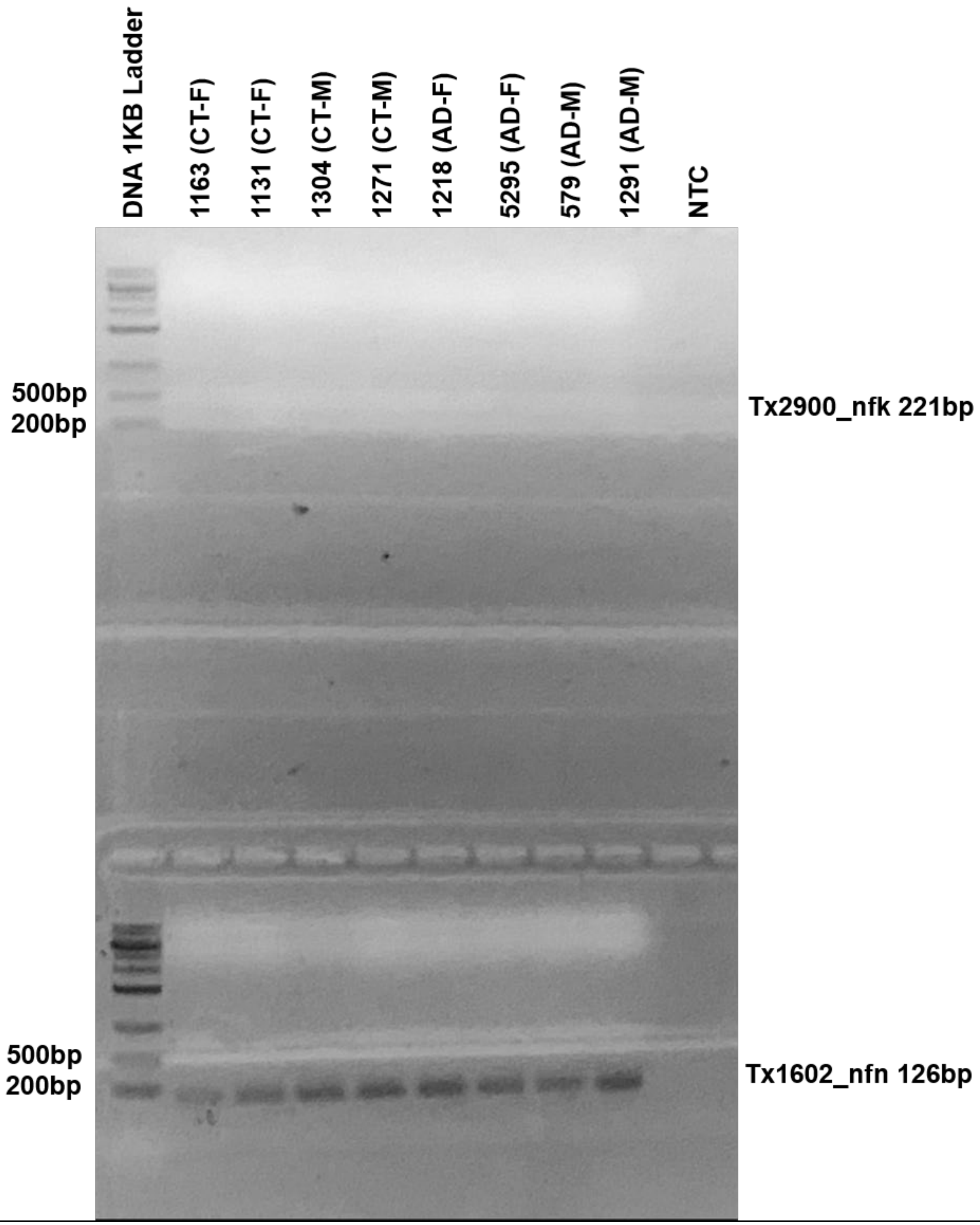

Supplementary Figure 17: PCR validation for new high confidence transcripts Tx2900 (*ENSG00000255122)* and Tx1602 (New gene body).

Number above the lanes is the sample id for 8 out of the 12 samples used in this study. The other 4 samples were not included because we ran out of tissue left for generating new cDNA. Labels on the right side of the figure indicate transcript id for the new high-confidence RNA isoform being validated and the expected product length from the PCR primers used to amplify the RNA isoform. Tx2900 is a new RNA isoform from the *ENSG00000255122* gene and was not validated in this gel, however it was validated through RT-qPCR (**Supplementary Table 5**). Tx1602 is a new RNA isoform from a newly discovered gene body and was succesfully validated in this gel. This gel electrophoresis PCR experiment was independently performed one time with similar results.

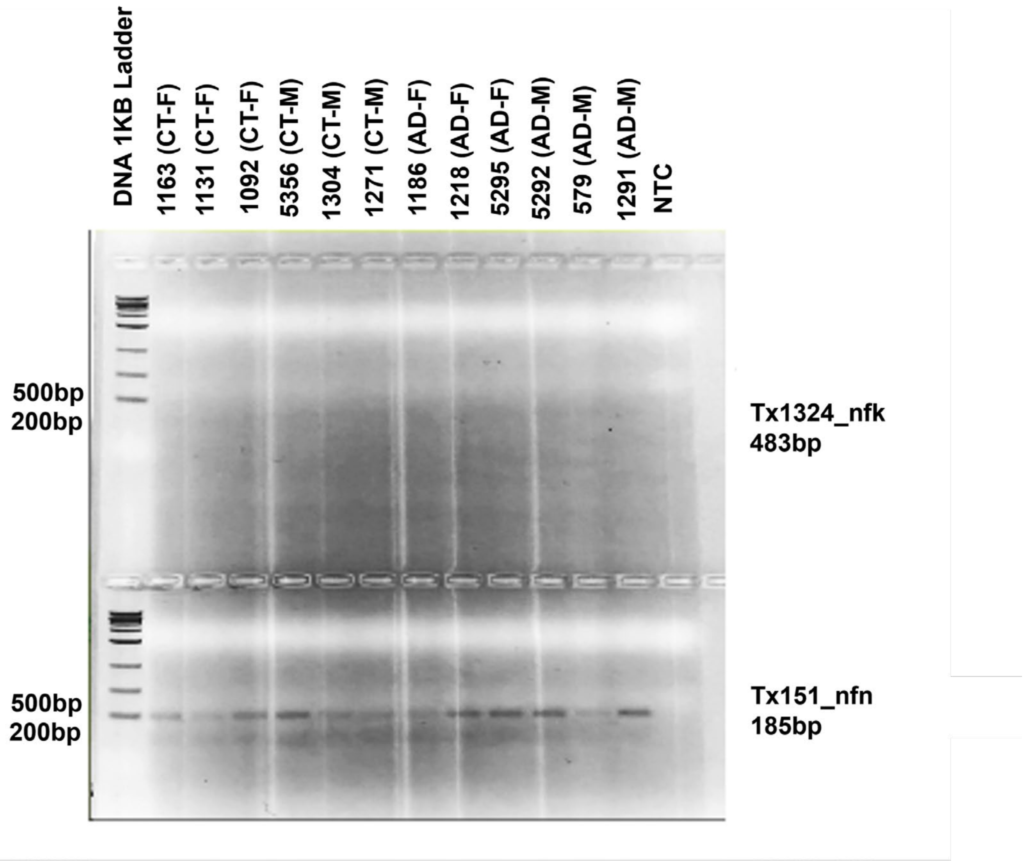

Supplementary Figure 18: PCR validation for new high confidence transcripts Tx1324 (*NELFA)* and Tx151 (*New gene body*).

Number above the lanes is the sample id for the 12 samples used in this study. Labels on the right side of the figure indicate transcript id for the new high-confidence RNA isoform being validated and the expected product length from the PCR primers used to amplify the RNA isoform. Tx1324 is a new RNA isoform from the *NELFA* gene and was not validated in this gel, but was validated in **Supplementary Figure 6**. Tx151 is a new RNA isoform from a newly discovered gene body and was validated in this gel (double bands), but was succesfully validated in **Supplementary Figure 5**. This gel electrophoresis PCR experiment was independently performed one time with similar results.

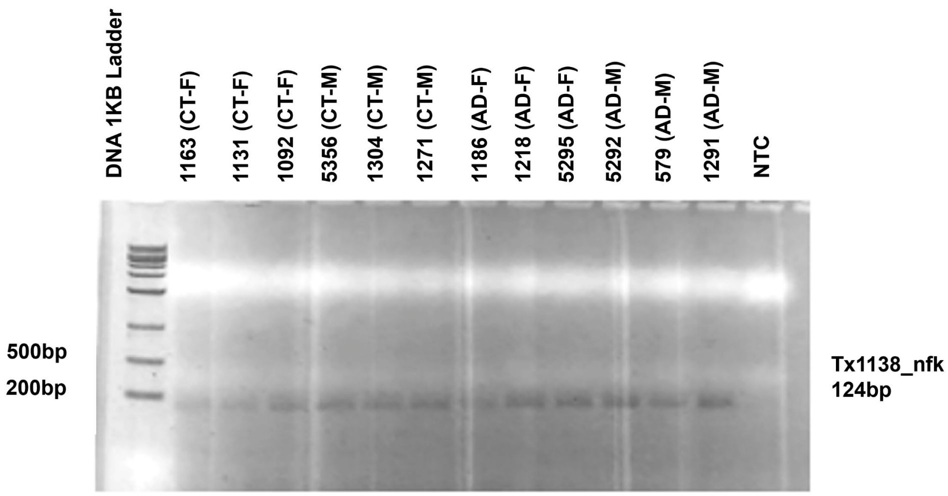

Supplementary Figure 19: PCR validation for new high confidence transcript Tx1138 (*IFNAR1)*

Number above the lanes is the sample id for the 12 samples used in this study. Labels on the right side of the figure indicate transcript id for the new high-confidence RNA isoform being validated and the expected product length from the PCR primers used to amplify the RNA isoform. Tx1138 is a new RNA isoform from the *IFNAR1* gene and was succesfully validated. This gel electrophoresis PCR experiment was independently performed one time with similar results.

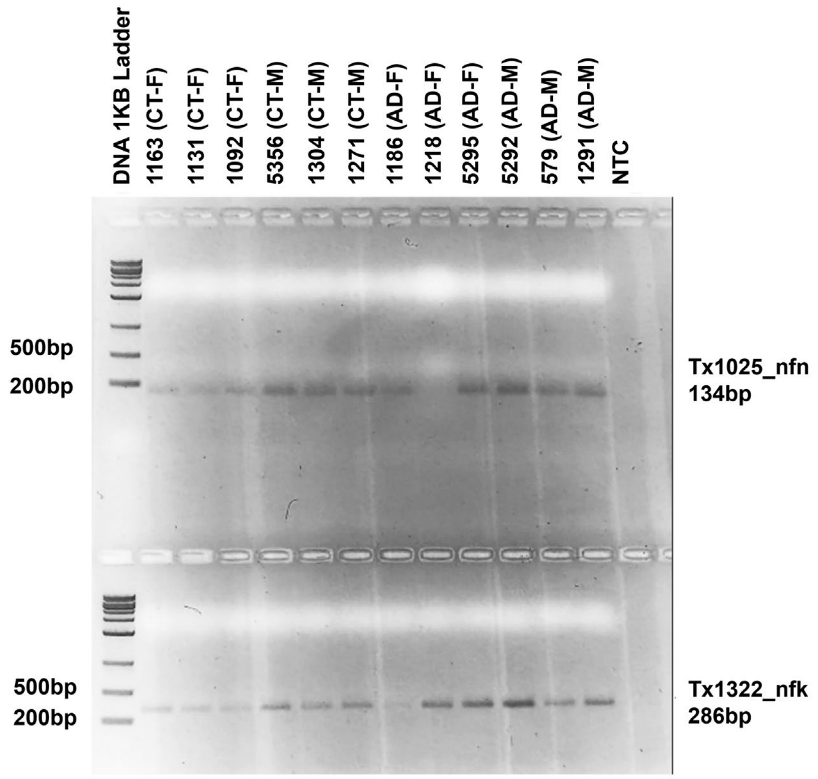

Supplementary Figure 20: PCR validation for new high confidence transcripts Tx1025 (New gene body) and Tx1322 (*SLC26A1)*.
Number above the lanes is the sample id for the 12 samples used in this study. Labels on the right side of the figure indicate transcript id for the new high-confidence RNA isoform being validated and the expected product length from the PCR primers used to amplify the RNA isoform. Tx1025 is a new RNA isoform from a newly discovered gene body and was succesfully validated. Tx1322 is a new RNA isoform for the *SLC26A1* gene and was succesfully validated. This gel electrophoresis PCR experiment was independently performed one time with similar results.

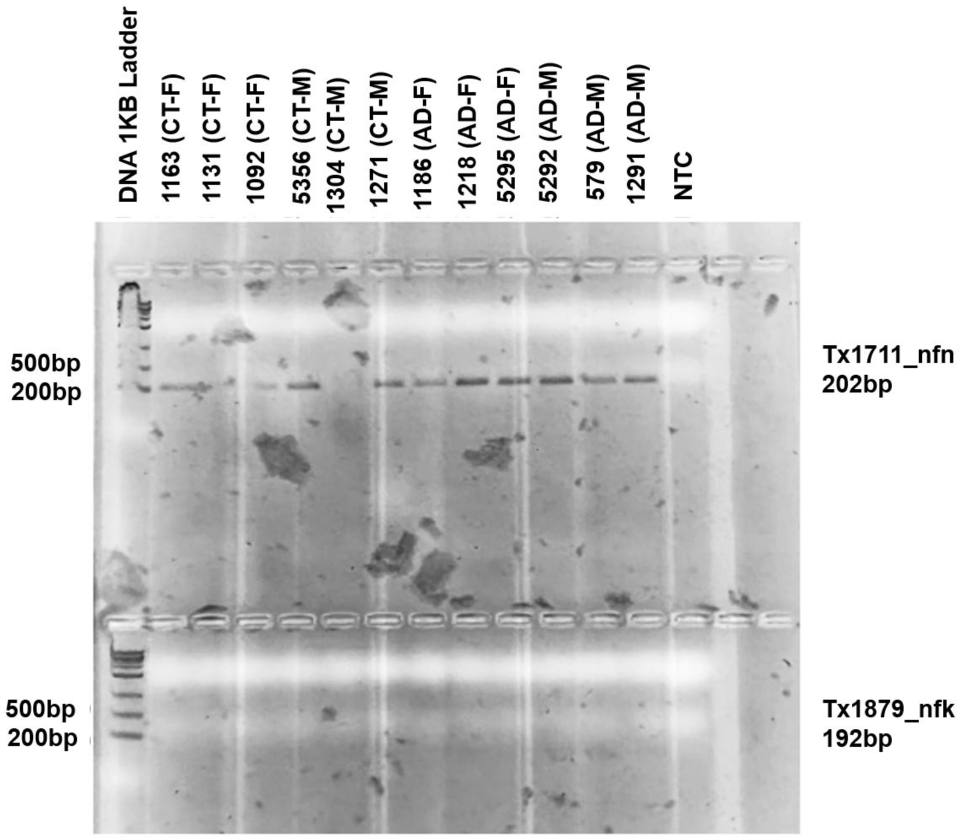

Supplementary Figure 21: PCR validation for new high confidence transcripts Tx1711 (New gene body) and Tx1879 (*MAOB*).
Number above the lanes is the sample id for the 12 samples used in this study. Labels on the right side of the figure indicate transcript id for the new high-confidence RNA isoform being validated and the expected product length from the PCR primers used to amplify the RNA isoform. Tx1711 is a new RNA isoform from a newly discovered gene body and was succesfully validated. Tx1879 is a new RNA isoform for the *MAOB* gene and was not validated in this gel, but was validated with a different primer pair in **Supplementary Figure 16**. This gel electrophoresis PCR experiment was independently performed one time with similar results.

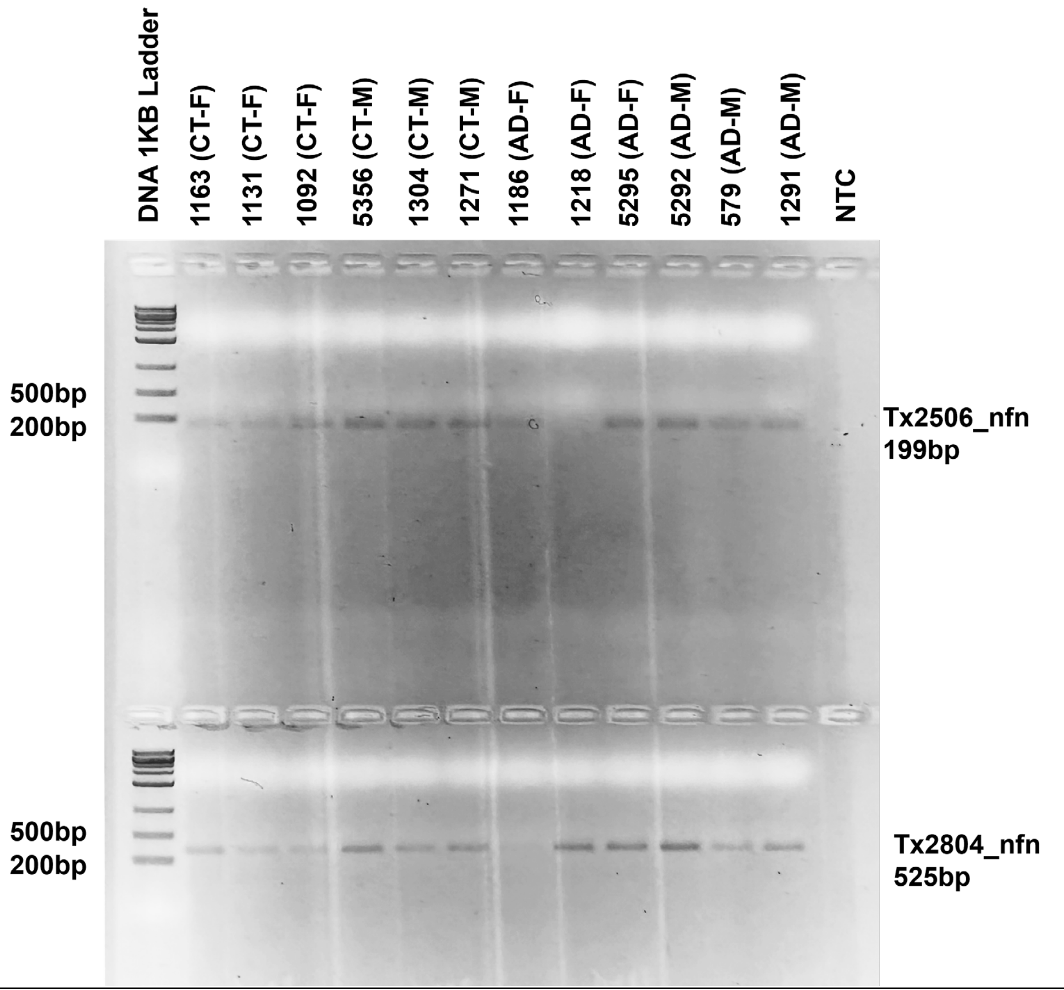

Supplementary Figure 22: PCR validation for new high confidence transcripts Tx2506 (New gene body) and Tx2804 (New gene body). Number above the lanes is the sample id for the 12 samples used in this study. Labels on the right side of the figure indicate transcript id for the new high-confidence RNA isoform being validated and the expected product length from the PCR primers used to amplify the RNA isoform. Tx2506 is a new RNA isoform from a new gene body and was succesfully validated. Tx2804 is a new RNA isoform from a new gene body and was not validated in this figure. We did not consider the Tx2804 validation succesful on this gel because the band is considerably smaller than the predicted 525 nucleotide PCR product. However, we ran another gel and succesfully validated it in **Supplementary Figure 23**. That PCR reaction was ran with a higher annealing temperature to prevent formation of primer dimers. This gel electrophoresis PCR experiment was independently performed one time with similar results.

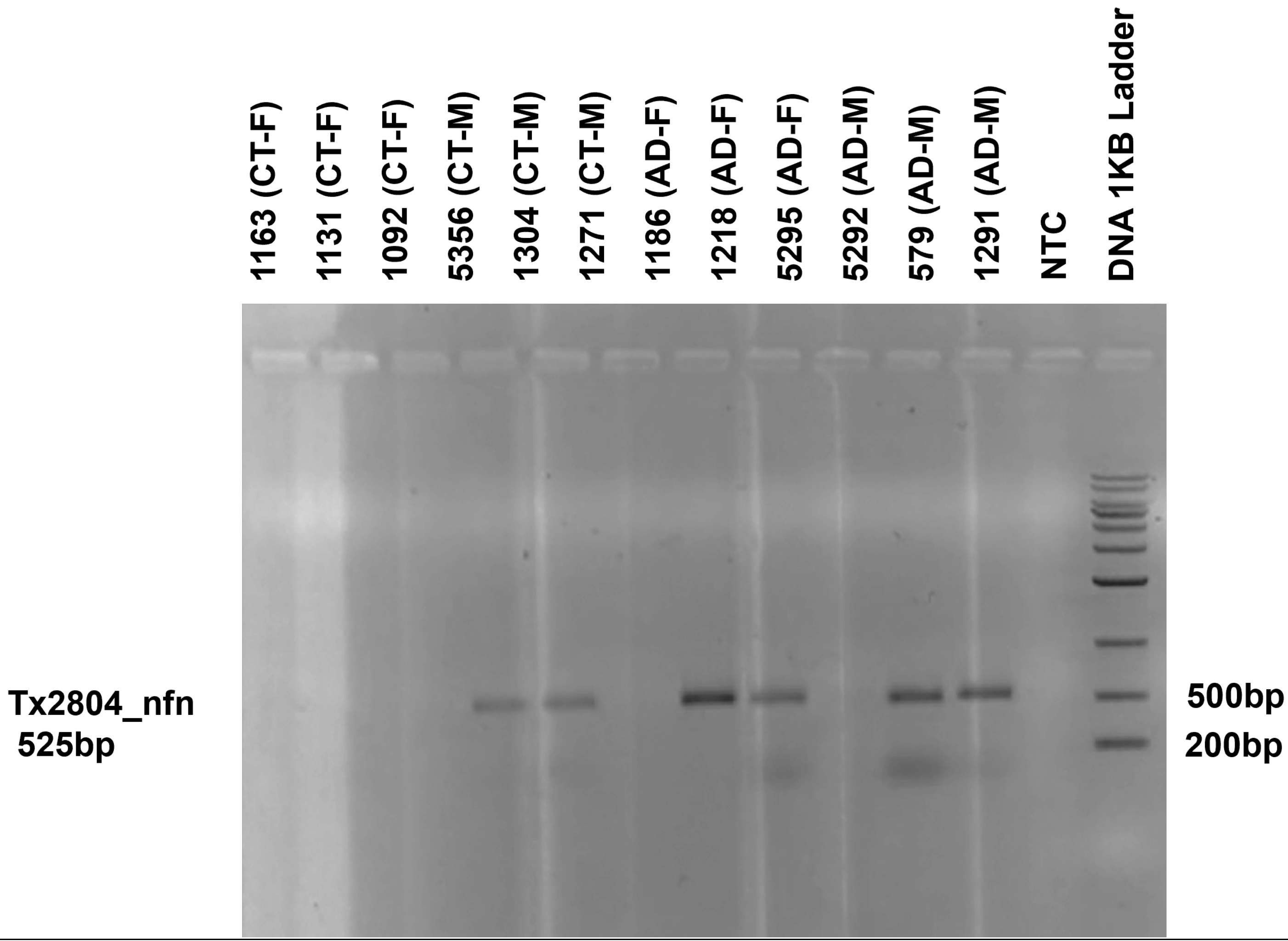

Supplementary Figure 23: Re-run of PCR validation for new high confidence transcript Tx2804 (New gene body).

Number above the lanes is the sample id for the 12 samples used in this study. Label on the left side of the figure indicate transcript id for the new high-confidence RNA isoform being validated and the expected product length from the PCR primers used to amplify the RNA isoform. Tx2804 is a new RNA isoform from a new gene body and was succesfully validated. This gel electrophoresis PCR experiment was independently performed one time with similar results.

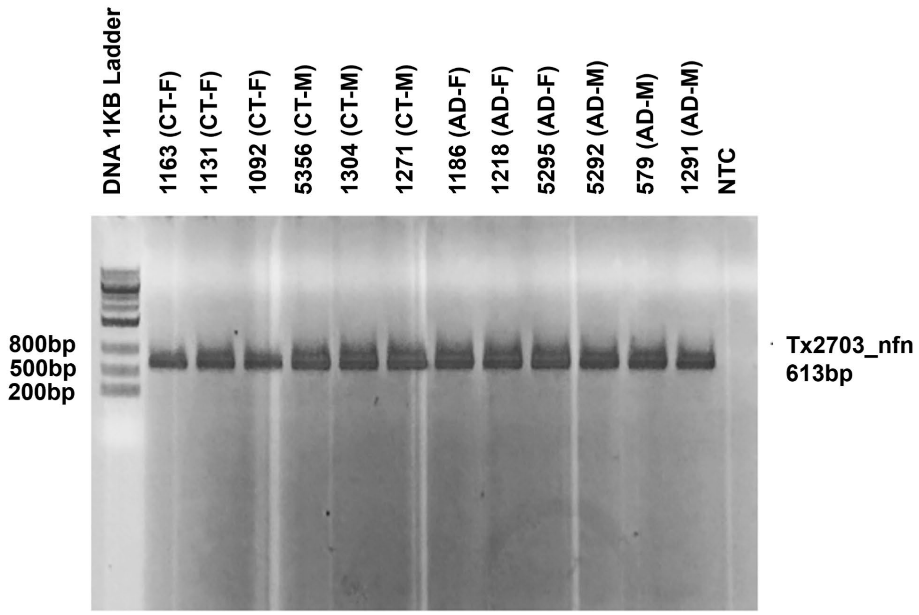

Supplementary Figure 24: PCR validation for new high confidence transcripts Tx2703 (New gene body).

Number above the lanes is the sample id for the 12 samples used in this study. Labels on the right side of the figure indicate transcript id for the new high-confidence RNA isoform being validated and the expected product length from the PCR primers used to amplify the RNA isoform. Tx2703 is a new RNA isoform from a newly discovered gene body and was succesfully validated. This gel electrophoresis PCR experiment was independently performed one time with similar results.

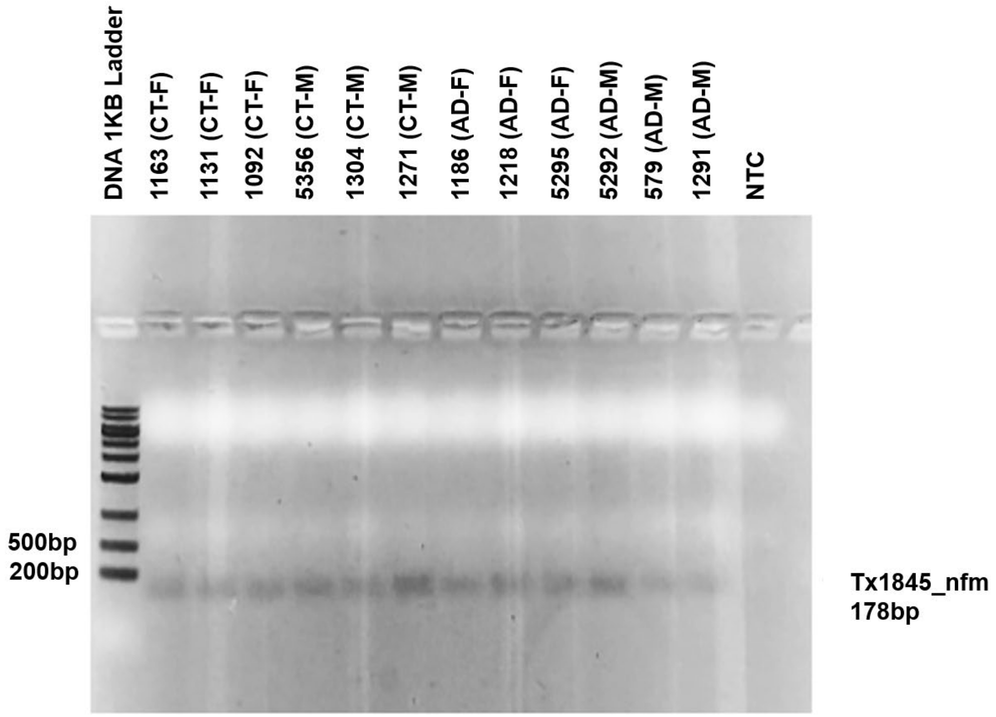

Supplementary Figure 25: PCR validation for new high confidence transcripts Tx1845 (New spliced mitochondrial isoform).
Number above the lanes is the sample id for the 12 samples used in this study. Labels on the right side of the figure indicate transcript id for the new high-confidence RNA isoform being validated and the expected product length from the PCR primers used to amplify the RNA isoform. Tx1845 is a new spliced RNA isoform from mitochondria and was succesfully validated. This gel electrophoresis PCR experiment was independently performed one time with similar results.

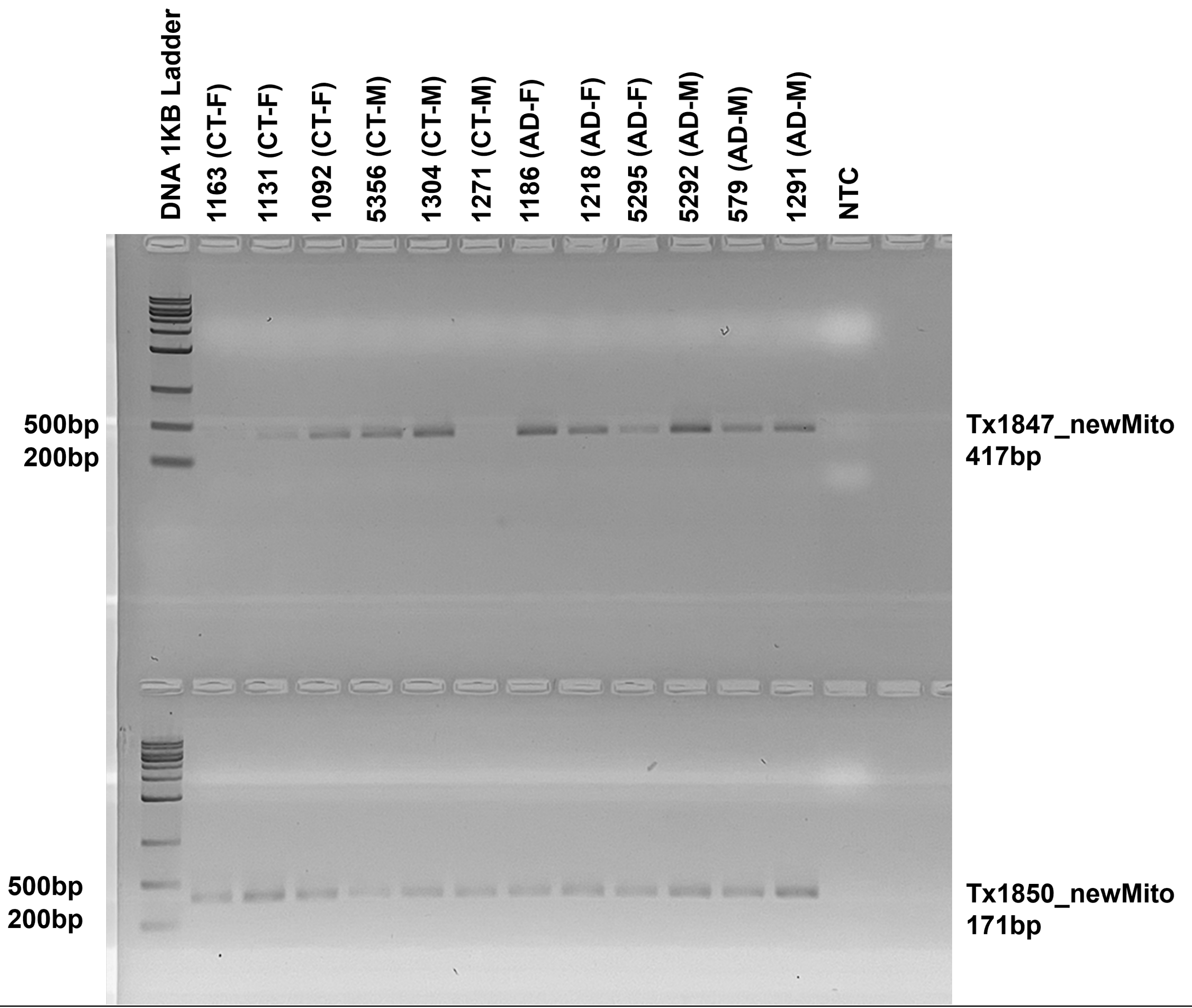

Supplementary Figure 26: PCR validation for new high confidence transcripts Tx1847 and Tx1850 (New spliced mitochondrial isoforms).

Number above the lanes is the sample id for the 12 samples used in this study. Labels on the right side of the figure indicate transcript id for the new high-confidence RNA isoform being validated and the expected product length from the PCR primers used to amplify the RNA isoform. Tx1847 is a new spliced RNA isoform from mitochondria and was succesfully validated. Tx1850 is a new spliced RNA isoform from the mitochondria and was not validated. We did not consider the Tx1850 validation succesful on this gel because the band is considerably larger than the predicted 171 nucleotide PCR product. This gel electrophoresis PCR experiment was independently performed one time with similar results.

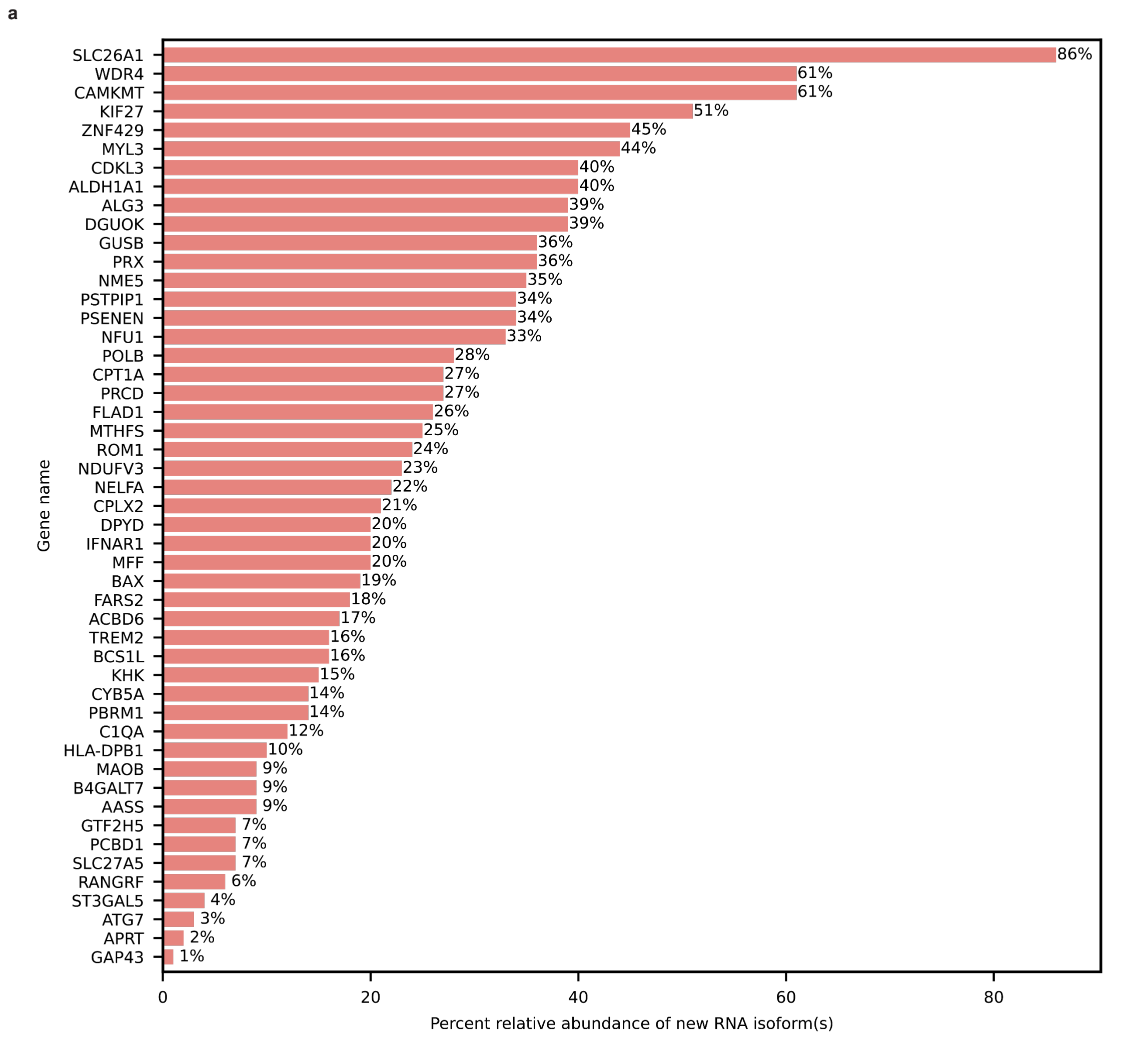

Supplementary Figure 27: Percent relative abundance of new RNA isoforms in medically relevant genes.

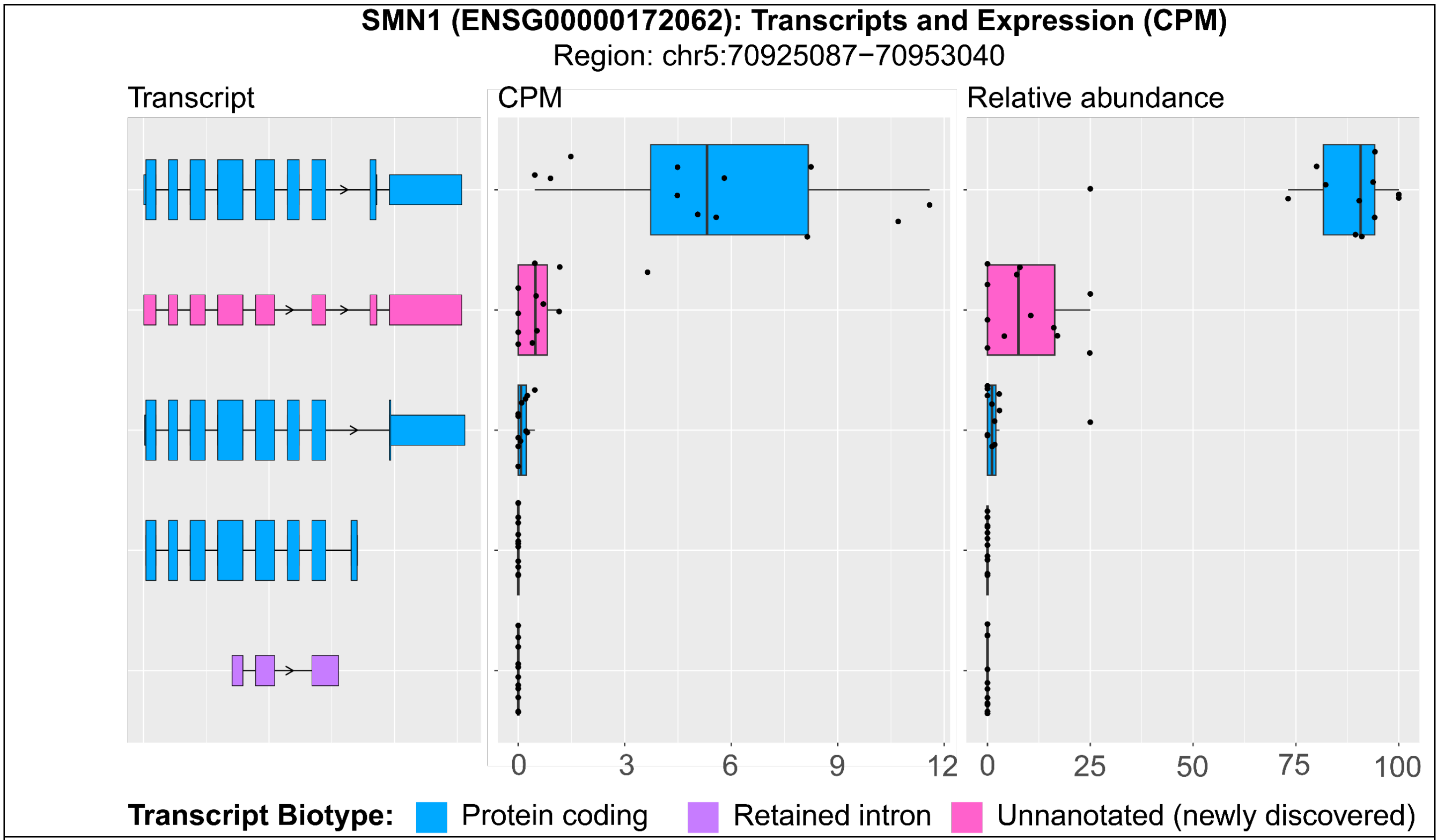

Supplementary Figure 28: RNA isoform expression and structure for SMN1.
RNA isoform structure, CPM normalized expression, and relative abundance for SMN1 RNA isoforms. We only show the top 5 most highly expressed RNA isoforms for this gene. The new RNA isoform is shown in pink. This new isoform did not meet our high-confidence threshold (median CPM > 1), but we report it for interest because of the gene’s direct role in neurodegenerative disease. All boxplots in this panel come from n=12 biologically independent samples. All boxplots in this panel follow this format: center line, median; box limits, upper and lower quartiles; whiskers, 1.5x interquartile range.

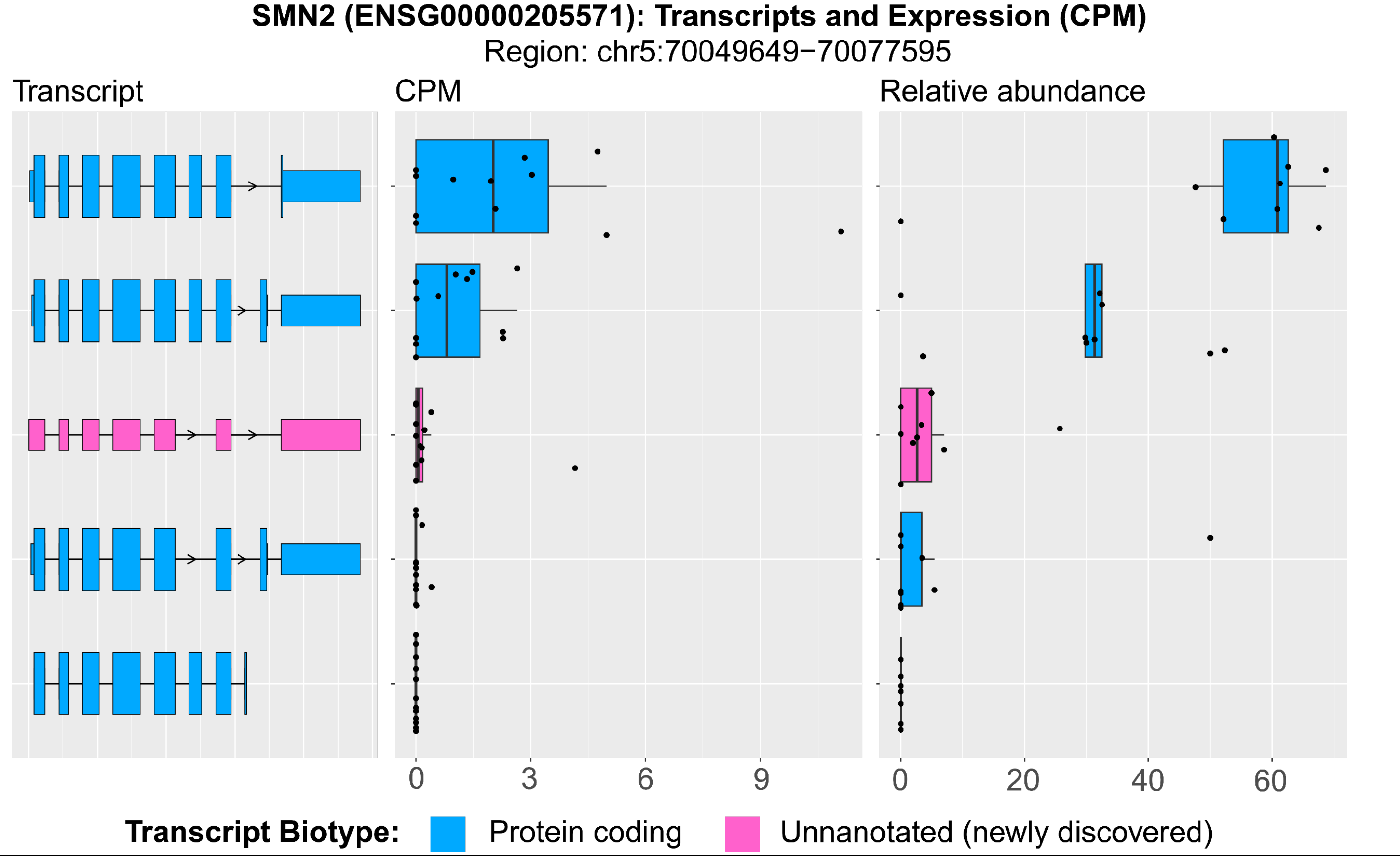

Supplementary Figure 29: RNA isoform expression and structure for SMN2.
RNA isoform structure, CPM normalized expression, and relative abundance for SMN2 RNA isoforms. We only show the top 5 most highly expressed RNA isoforms for this gene. The new RNA isoform is shown in pink. This new isoform did not meet our high-confidence threshold (median CPM > 1), but we report it for interest because of the gene's direct role in neurodegenerative disease. All boxplots in this panel come from n=12 biologically independent samples. All boxplots in this panel follow this format: center line, median; box limits, upper and lower quartiles; whiskers, 1.5x interquartile range.

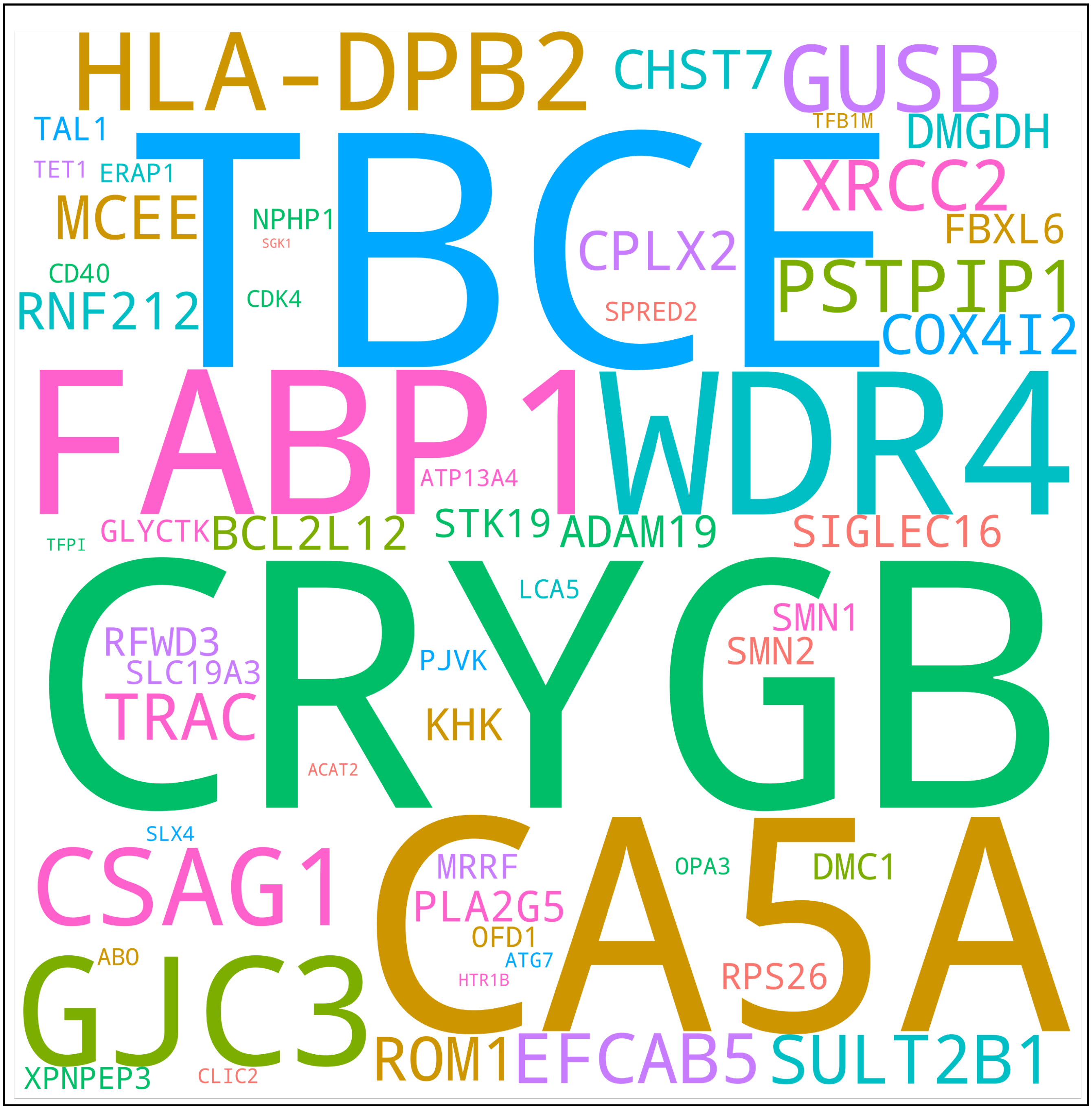

Medically relevant genes with new high-confidence RNA isoform(s). Font size is proportional to relative abundance of the new isoform(s).
TET1 = 4% | SMN1 = 11%| ADAM19= 15% | XRCC2 = 36% | FABP1= 61% | CRYGB = 95%

Supplementary Figure 30: Medically relevant genes with RNA isoforms expressed at median CPM ≤ 1 in human frontal cortex. Gene names for medically relevant genes where we discovered a new RNA isoform that was not annotated in Ensembl version 107. Only included new RNA isoforms with a median CPM ≤ 1. The size of gene name is proportional to the relative abundance of the new RNA isoform(s).

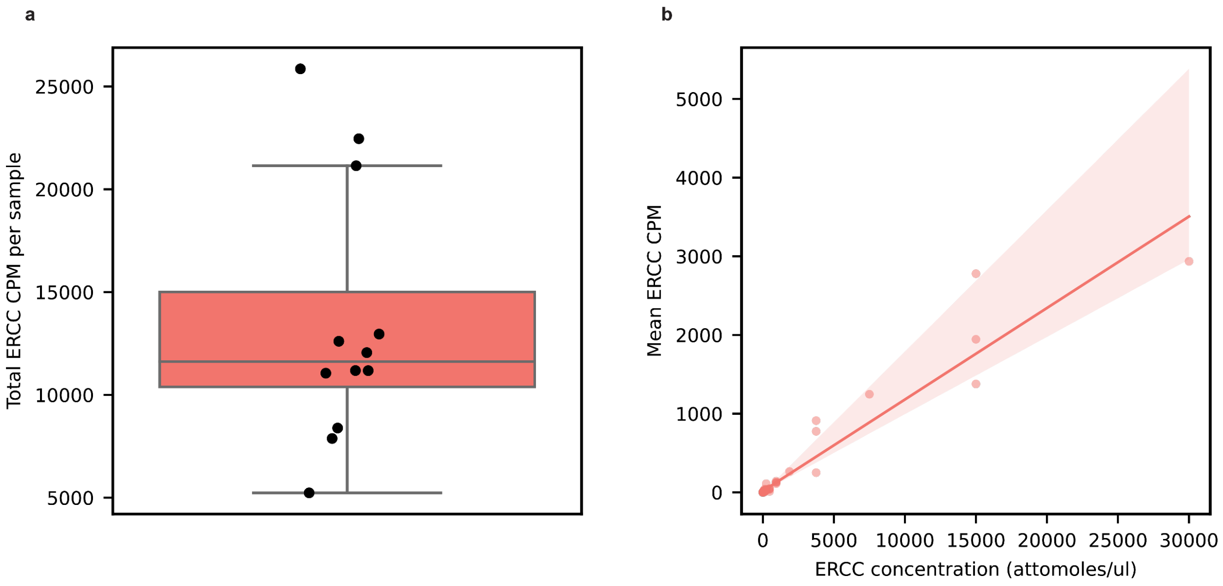

Supplementary Figure 31: ERCC spike-in expression estimates.
a, Sum of CPM for all ERCC control spike-in RNA transcripts for each sample. Definition of boxplot elements: center line, median; box limits, upper and lower quartiles; whiskers, 1.5x interquartile range. **b,** Correlation between ERCC transcript concentration and mean ERCC CPM for that same transcript in all 12 samples. Spearman correlation coefficient = 0.98. Shaded area in around the line of best fit represents the 95% confidence interval based in 1000 bootstrap resamples. All figures from this panel come from n=12 biologically independent samples.

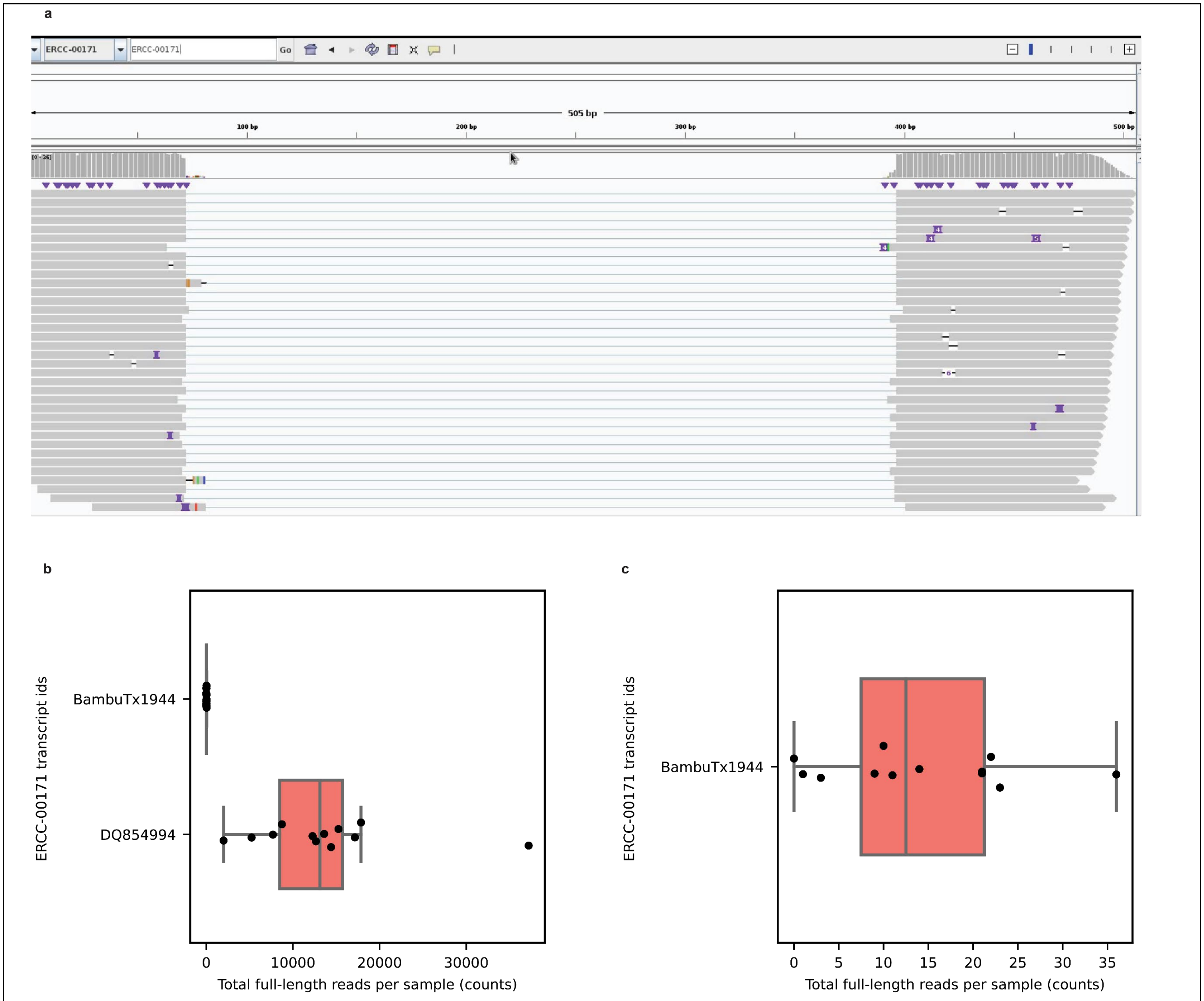

Supplementary Figure 32: Spliced ERCC spike-in isoform.
a, Interactive Genome Viewer (IGV) screenshot showing the “spliced” ERCC reads for one of our samples. **b,** Comparison between total number of full-length reads for the known ERCC transcript and the new spliced ERCC transcript that shares its nucleotide sequence. Each dot on the graph represents a sample. The new spliced ERCC transcript is present at very low concentration in comparison to the non-spliced ERCC transcript that shares its nucleotide sequence. **c,** Number of full-length reads for the new spliced ERCC transcript in each of the 12 samples. Figure **a** comes from n=1 biologically independent sample. Figures **b** and **c** come from n=12 biologically independent samples. All boxplots in this panel follow this format: center line, median; box limits, upper and lower quartiles; whiskers, 1.5x interquartile range.

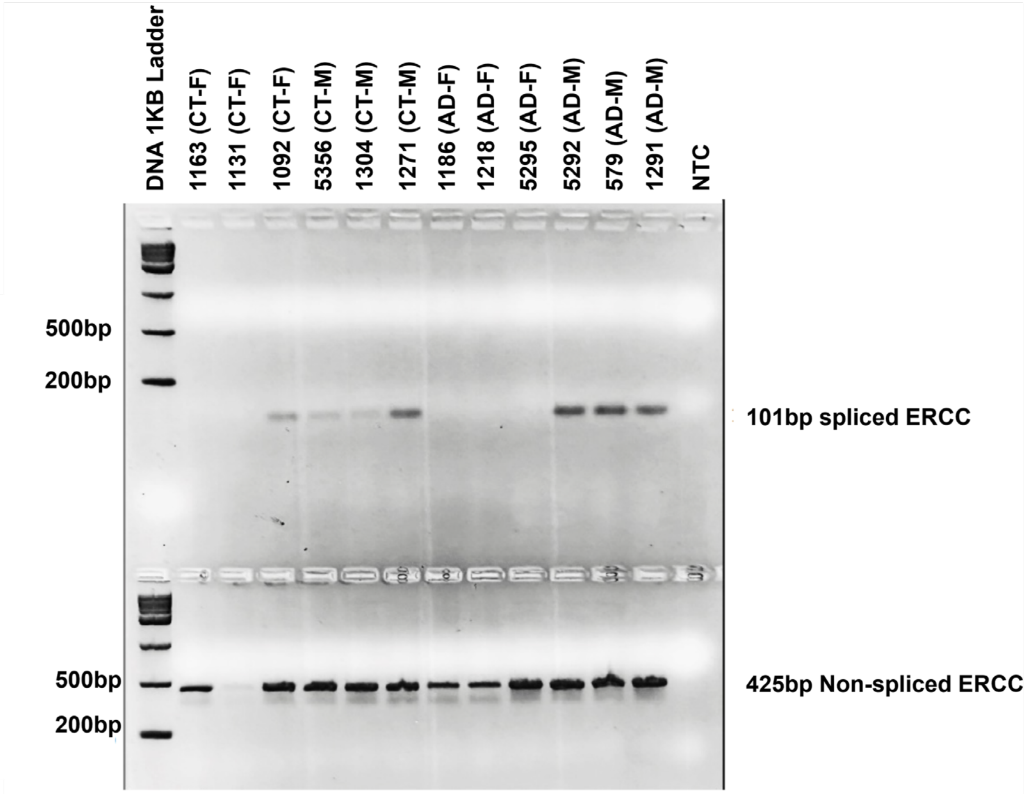

Supplementary Figure 33: PCR validation for spliced ERCC RNA isoform in 12 samples.

Number above the lanes is the sample id for the 12 samples used in this study. Labels on the right side of the figure indicate transcript id for the RNA isoform being validated and the expected product length from the PCR primers used to amplify the RNA isoform. ERCC spliced refers to the new spliced ERCC RNA isoform found in our data, which was succesfully validated. No splice ERCC refers to the regular ERCC sequence that is no spliced, which was also validated succesfully, as expected. This gel electrophoresis PCR experiment was independently performed one time with similar results.

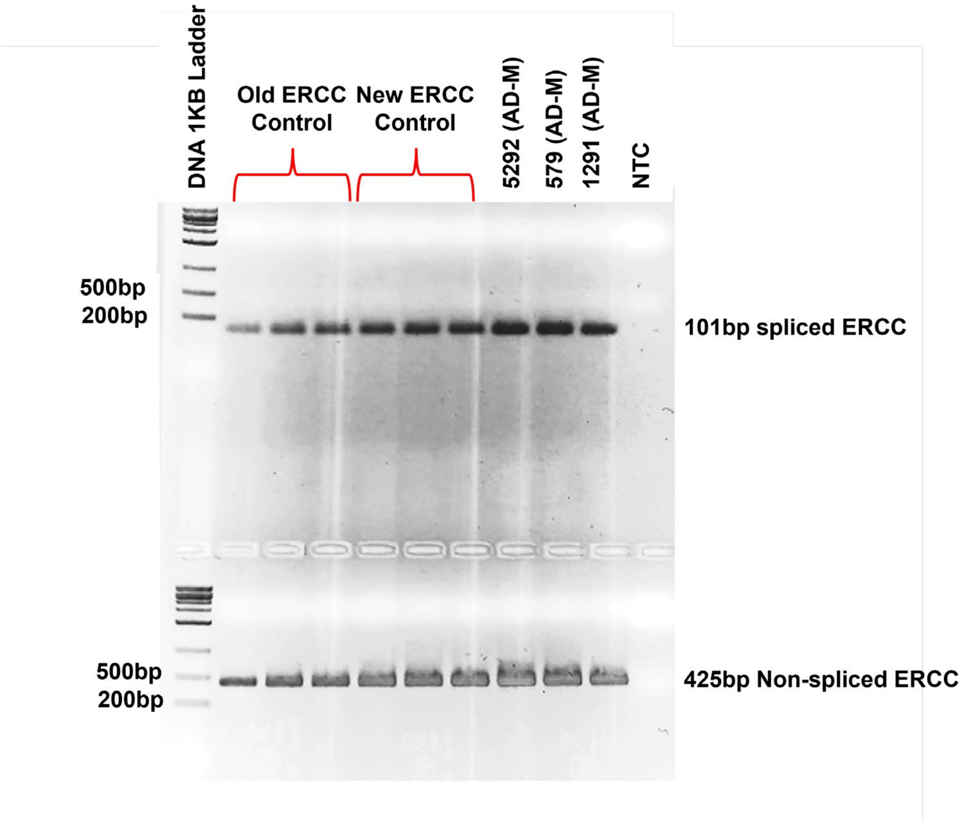

Supplementary Figure 34: PCR validation for spliced ERCC RNA isoform in two different ERCC batches and 4 samples.
Number above the lanes is the sample id for 4 samples used in this study, old ERCC control refers to an older ERCC batch whereas new ERCC refers to a fresh ERCC batch we tested with. Labels on the right side of the figure indicate transcript id for the RNA isoform being validated and the expected product length from the PCR primers used to amplify the RNA isoform. ERCC spliced refers to the new spliced ERCC RNA isoform found in our data, which was succesfully validated. No splice ERCC refers to the regular ERCC sequence that is no spliced, which was also validated succesfully as expected. This gel electrophoresis PCR experiment was independently performed one time with similar results.

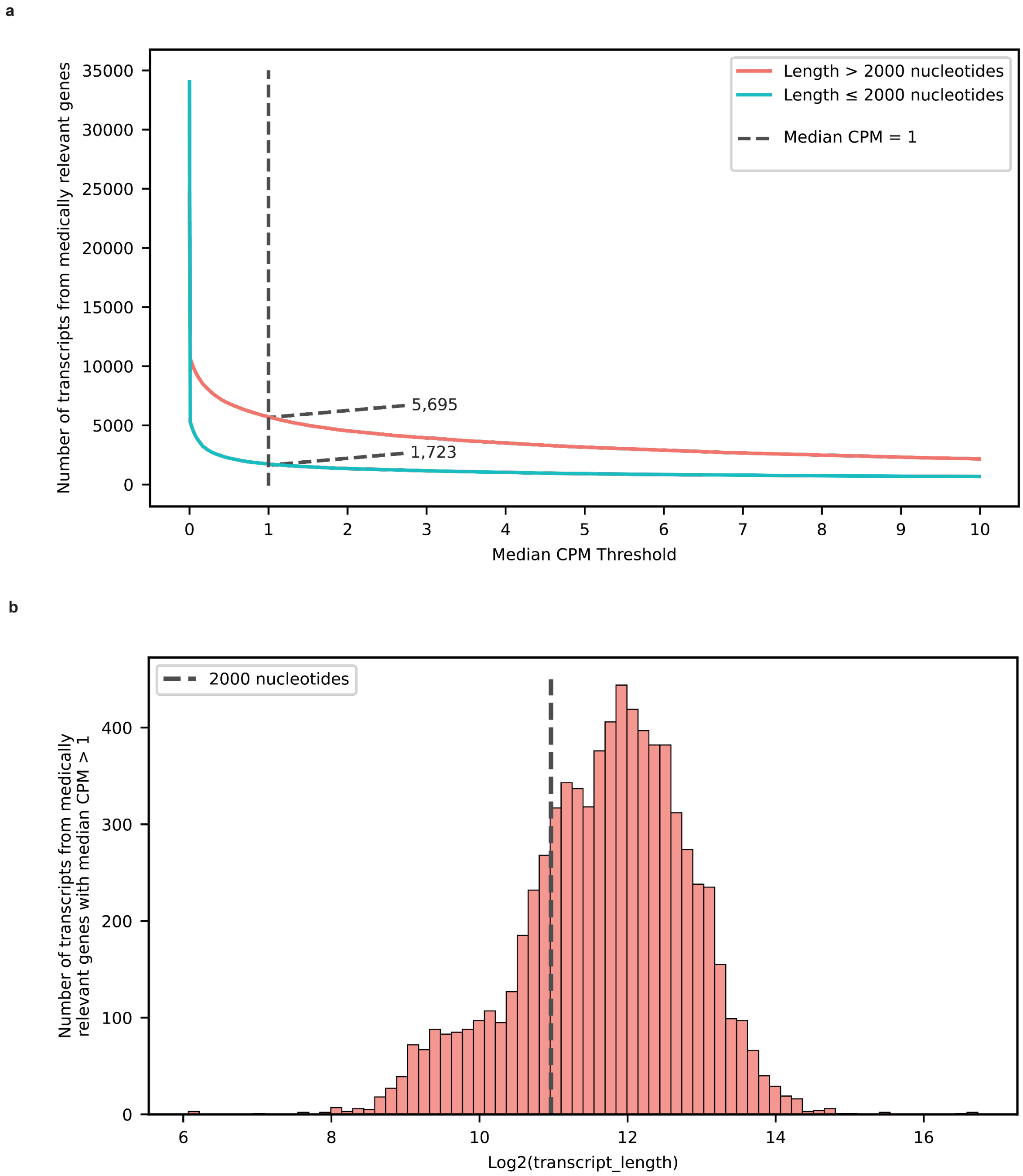

Supplementary Figure 35: Expression and length distribution for transcripts from medically relevant genes. a, Lineplot showing number of transcripts from medically relevant genes that are expressed across a range of median CPM thresholds in our data. The red/coral line show transcripts longer than 2,000 nucleotides and the blue/mint line shows trnascripts shorter or equal to 2,000 nucleotides. The dotted line is drawn at median CPM = 1 and the numbers by the line represent the number of transcripts expressed at median CPM > 1 for each transcript length category. **b,** Histogram showing the read length distribution for transcripts from medically relevant genes that our expressed in our data with median CPM > 1. Read length is shown in a log2 scale to avoid stretching the plot because of outliers.

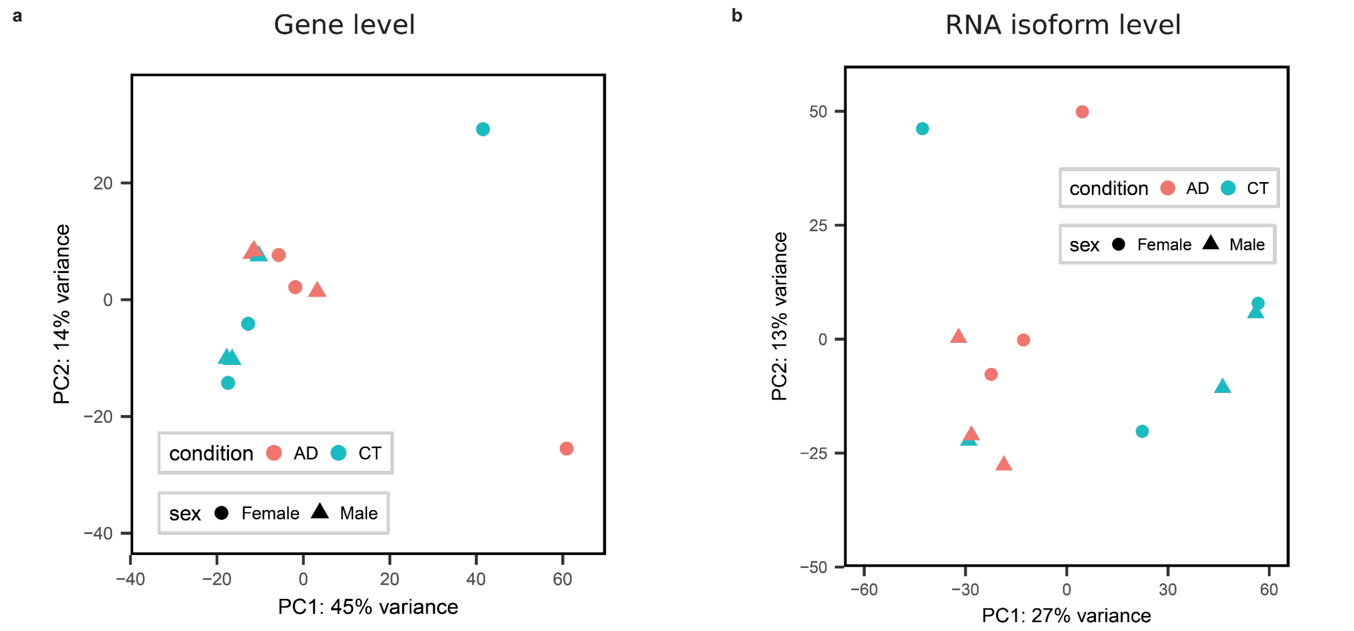

Supplementary Figure 36: Principal component analysis plots for gene level and RNA isoform level RNAseq data. a, Principal component analysis plots for gene level data generated using DEseq2 on unfiltered gene level counts. **b,** Principal component analysis plots for RNA isoform level data generated using DEseq2 on unfiltered gene level counts.

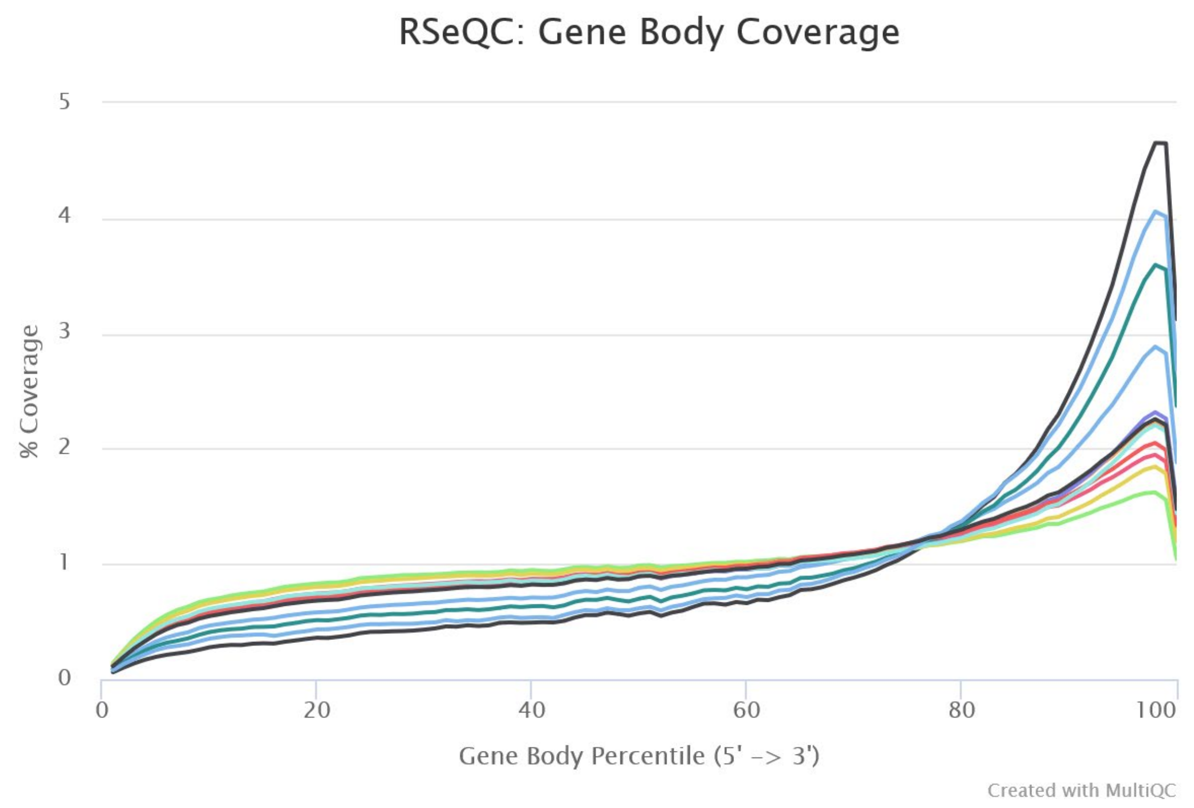

Supplementary Figure 37: 3' bias in long-read RNAseq samples from this study. Y-axis shows percent coverage of housekeeping transcripts and X-axis shows gene body percentile, where 0 is the 5' end and 100 is the 3' end. All samples have 3' bias, but some have a much stronger bias than others. Plot was generated with RseQC[1] using the "geneBody_coverage.py" module with the housekeeping BED file provided in: https://sourceforge.net/projects/rseqc/files/BED/Human_Homo_sapiens/hg38.HouseKeepingGenes.bed.gz/download.

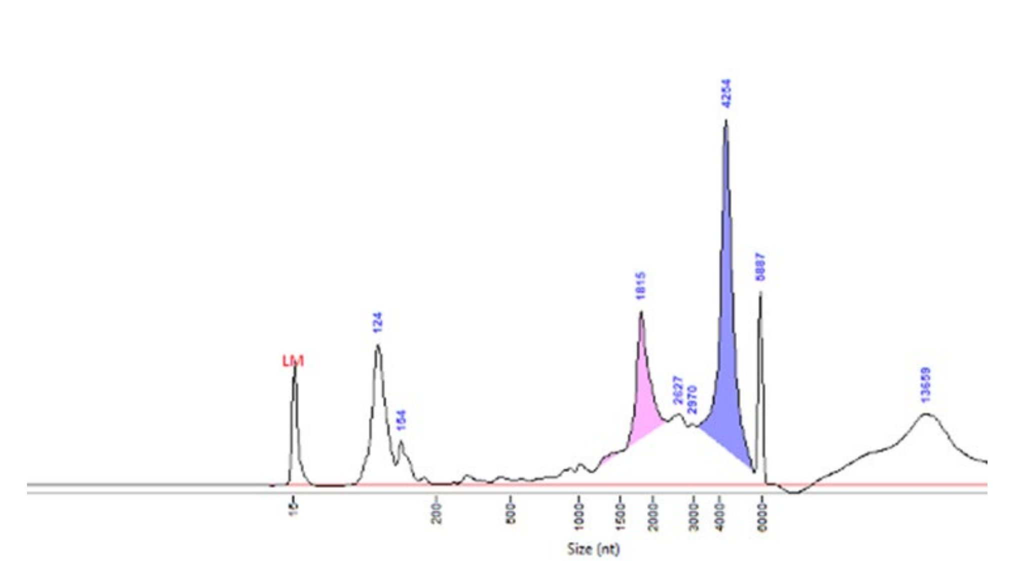

Supplementary Figure 38. Fragment Analyzer RNA trace for sample 1163 (Control female).

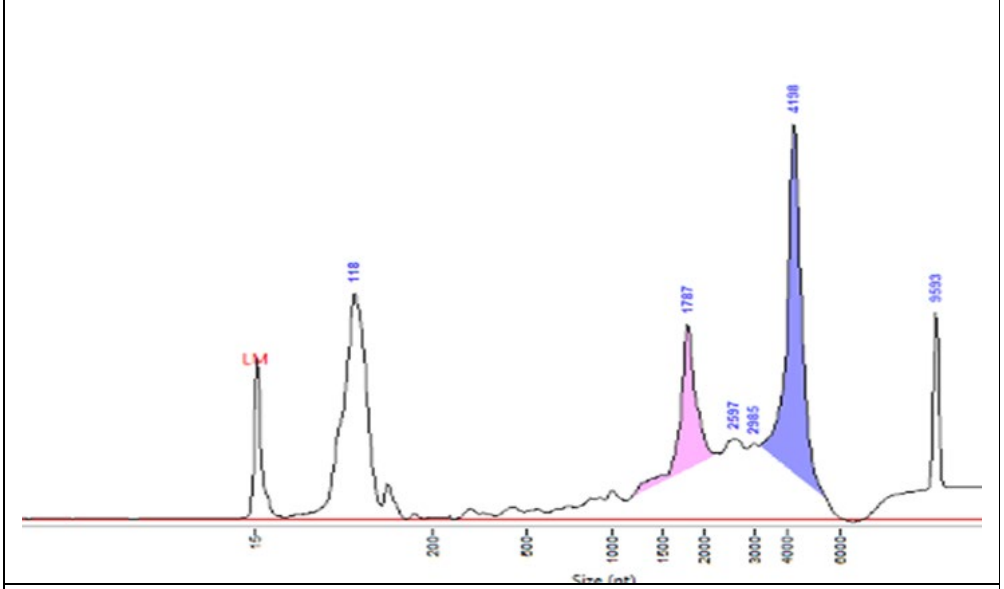

Supplementary Figure 39. Fragment Analyzer RNA trace for sample 1131 (Control female).

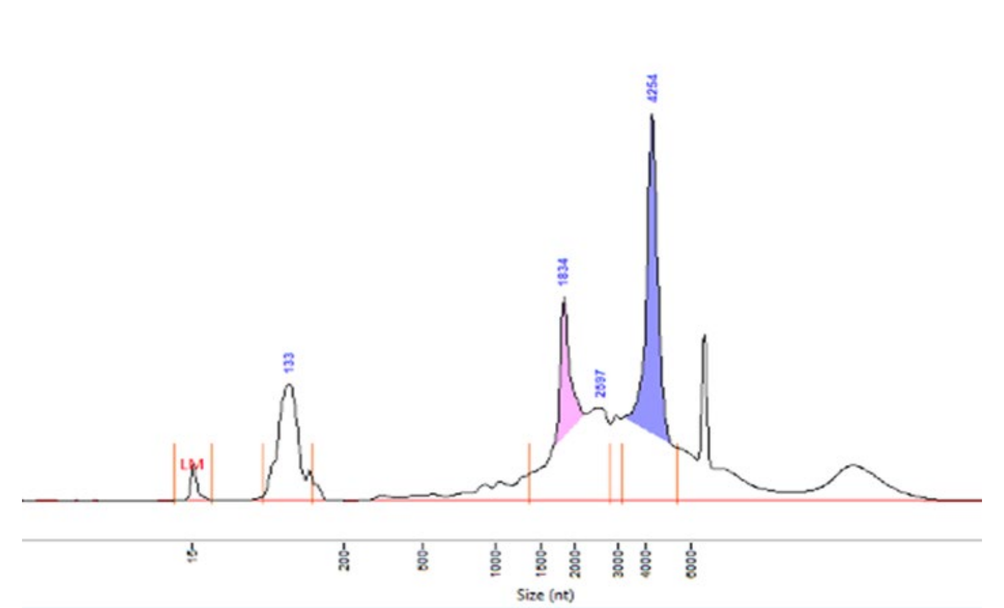

Supplementary Figure 40. Fragment Analyzer RNA trace for sample 1092 (Control female).

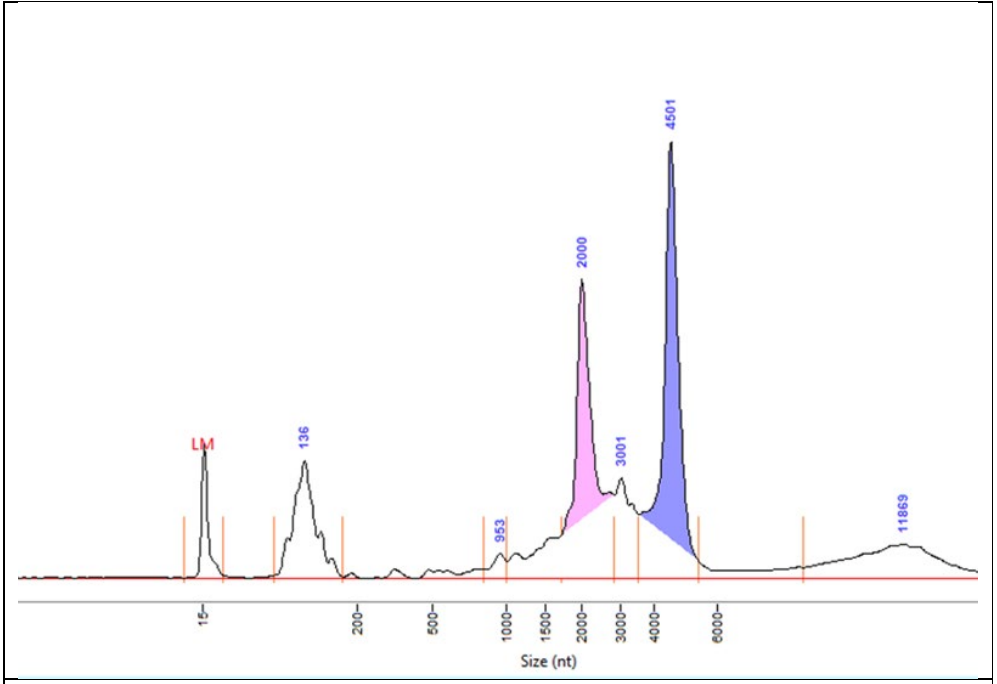

Supplementary Figure 41. Fragment Analyzer RNA trace for sample 5356 (Control male).

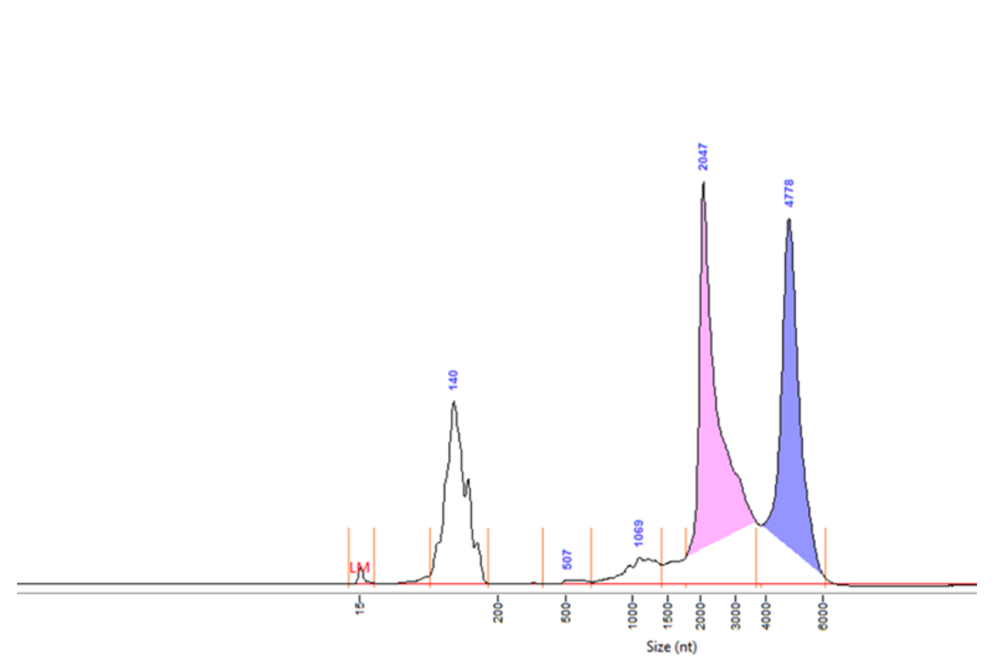

Supplementary Figure 42. Fragment Analyzer RNA trace for sample 1304 (Control male).

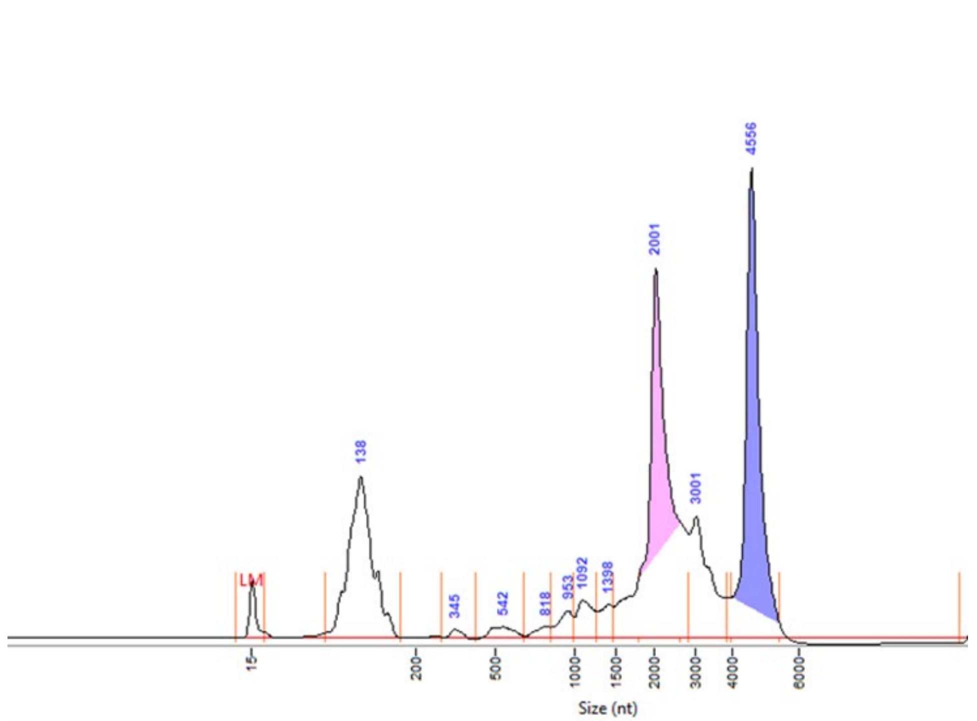

Supplementary Figure 43. Fragment Analyzer RNA trace for sample 1271 (Control male).

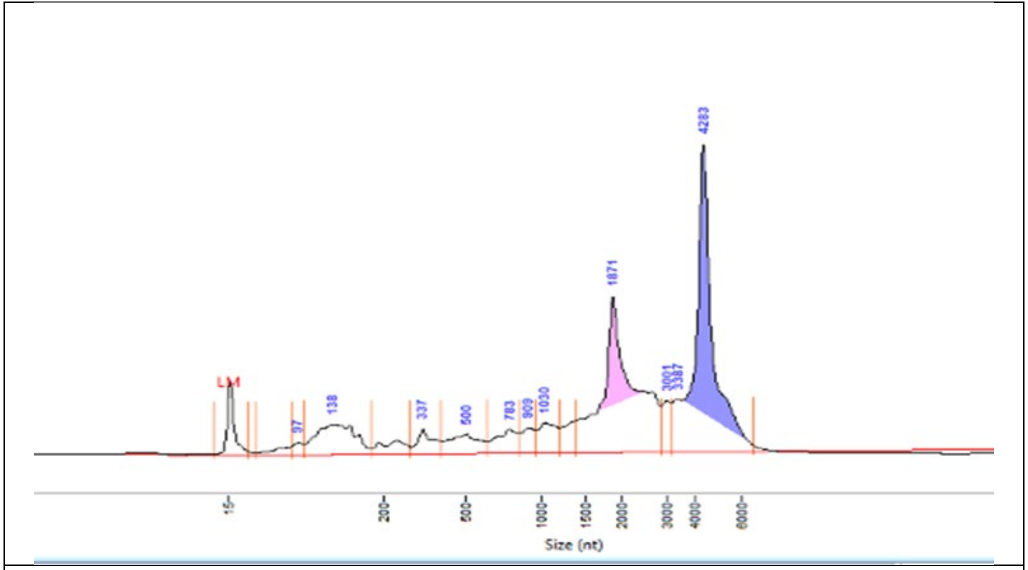

Supplementary Figure 44. Fragment Analyzer RNA trace for sample 1186 (Alzheimer's disease female).

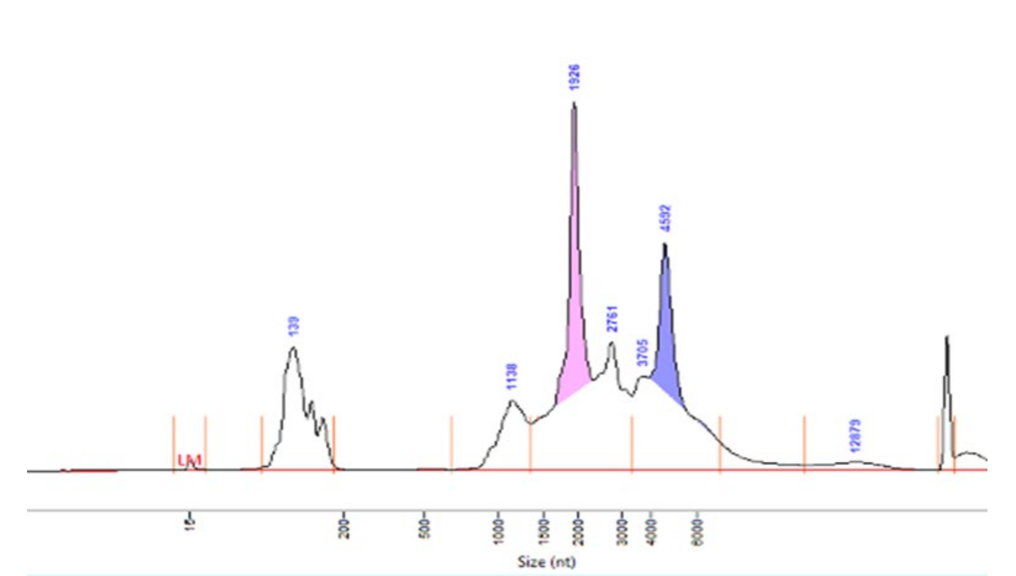

Supplementary Figure 45. Fragment Analyzer RNA trace for sample 1218 (Alzheimer's disease female).

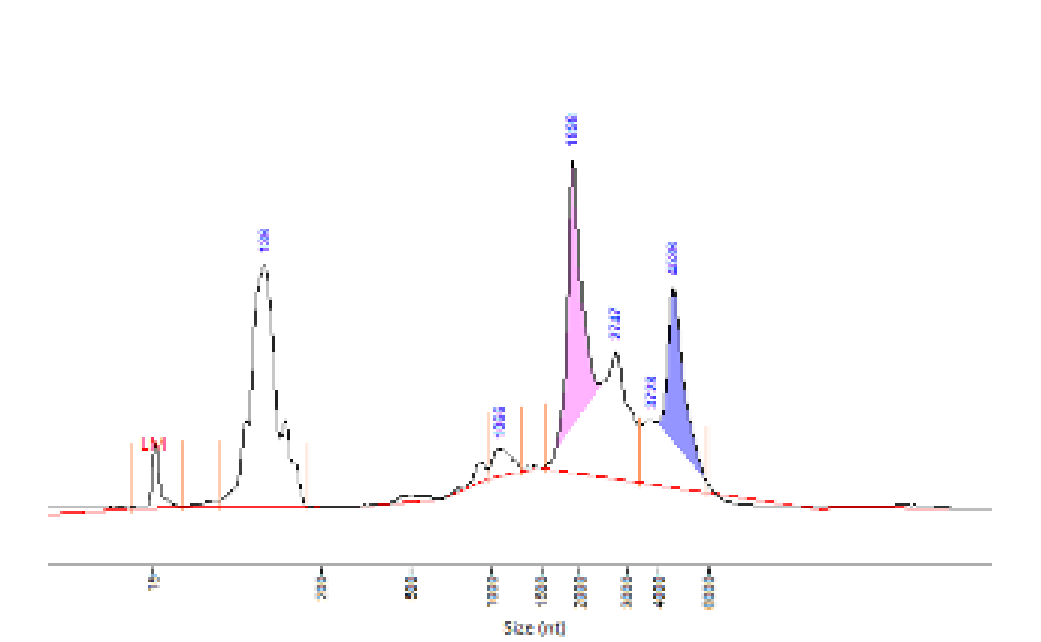

Supplementary Figure 46. Fragment Analyzer RNA trace for sample 5295 (Alzheimer's disease female).

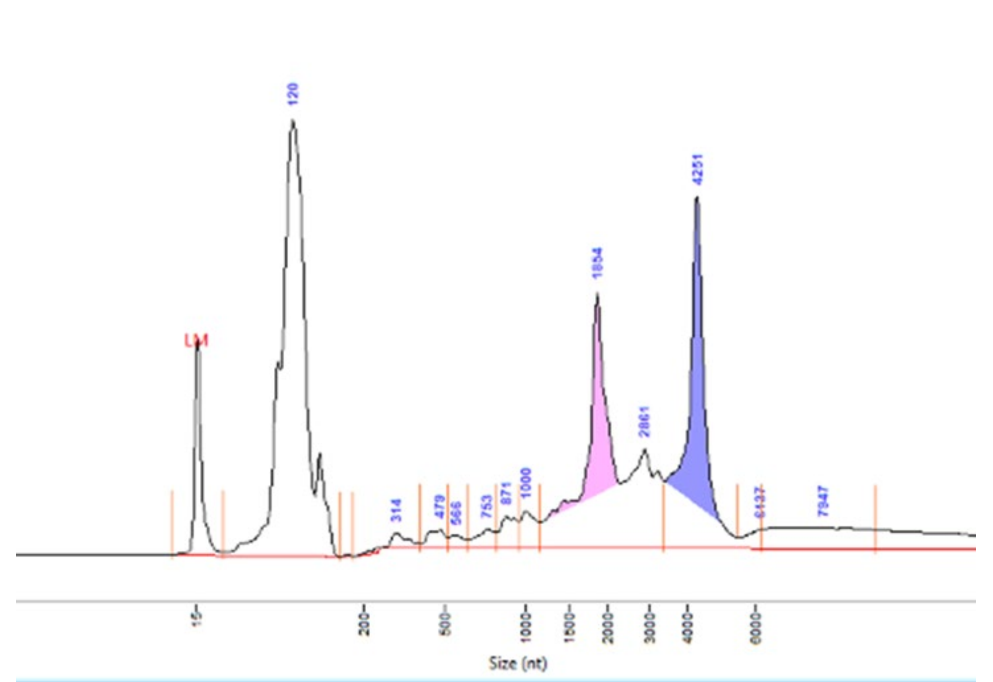

Supplementary Figure 47. Fragment Analyzer RNA trace for sample 5292 (Alzheimer's disease female).

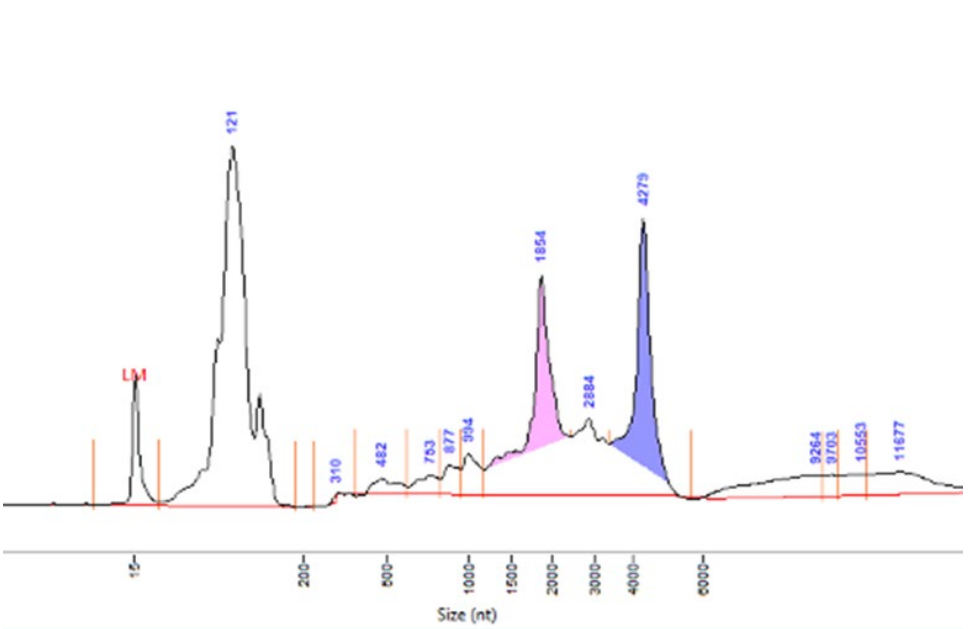

Supplementary Figure 48. Fragment Analyzer RNA trace for sample 579 (Alzheimer's disease female).

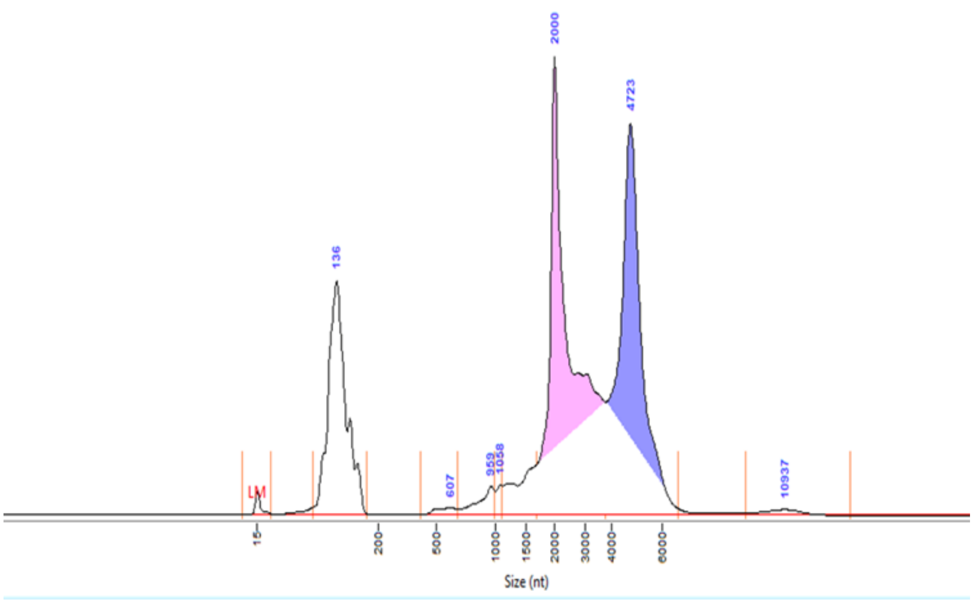

Supplementary Figure 49. Fragment Analyzer RNA trace for sample 1291 (Alzheimer's disease female).

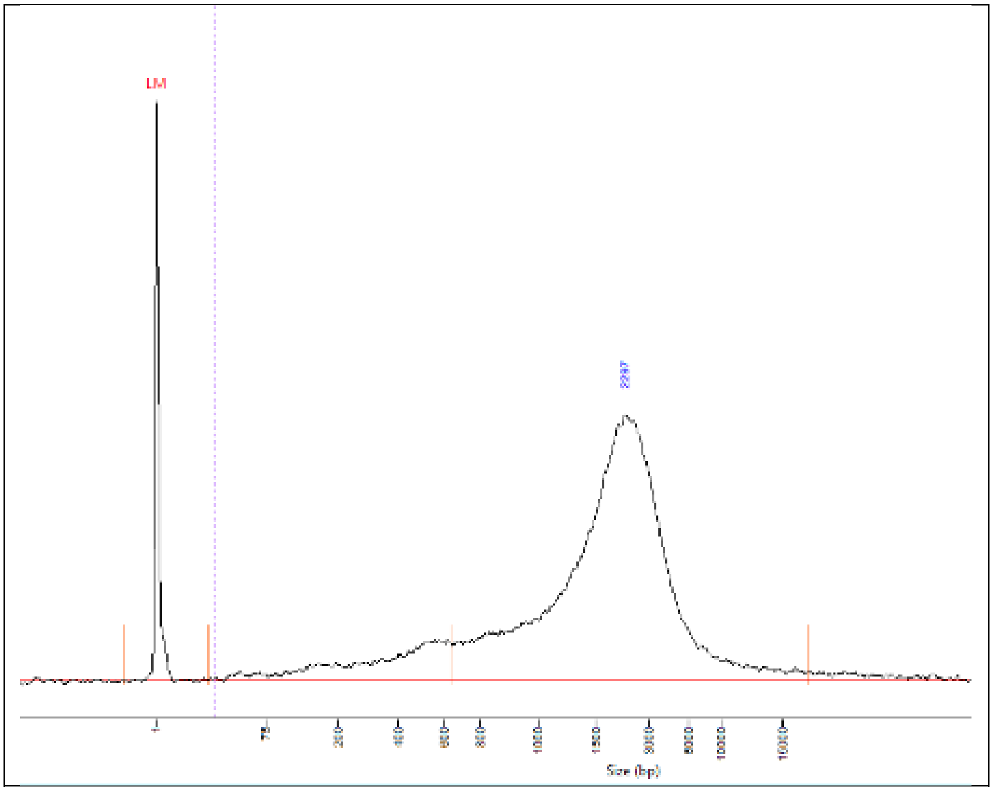

Supplementary Figure 50. Fragment Analyzer cDNA trace for sample 1163 (Control female). Peak at 2297 nucleotides.

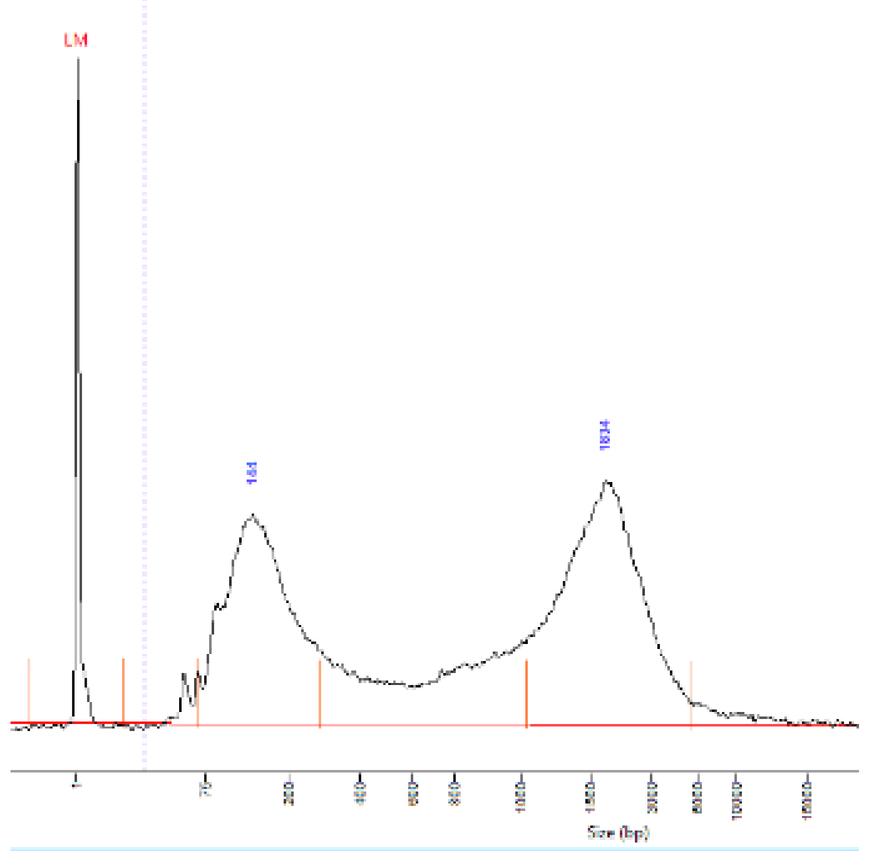

Supplementary Figure 51. Fragment Analyzer cDNA trace for sample 1131 (Control female). Lower peak at 144 nucleotides, higher peak at 1834 nucleotides

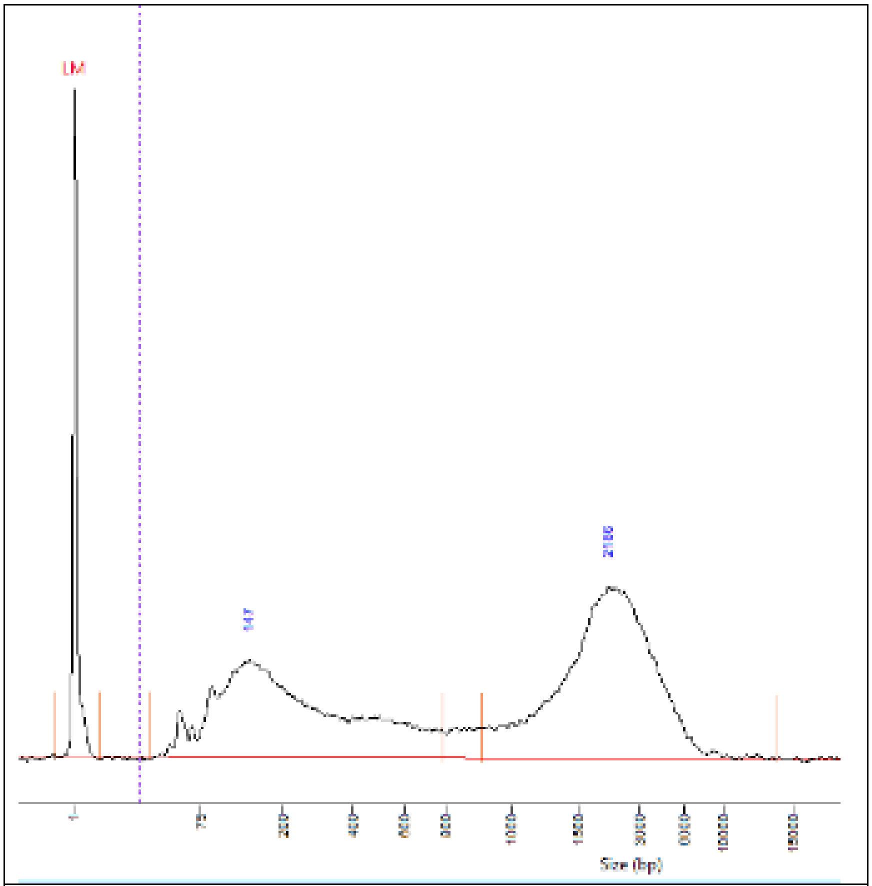

Supplementary Figure 52. Fragment Analyzer cDNA trace for sample 1092 (Control female). Lower peak at 147 nucleotides, higher peak at 2186 nucleotides

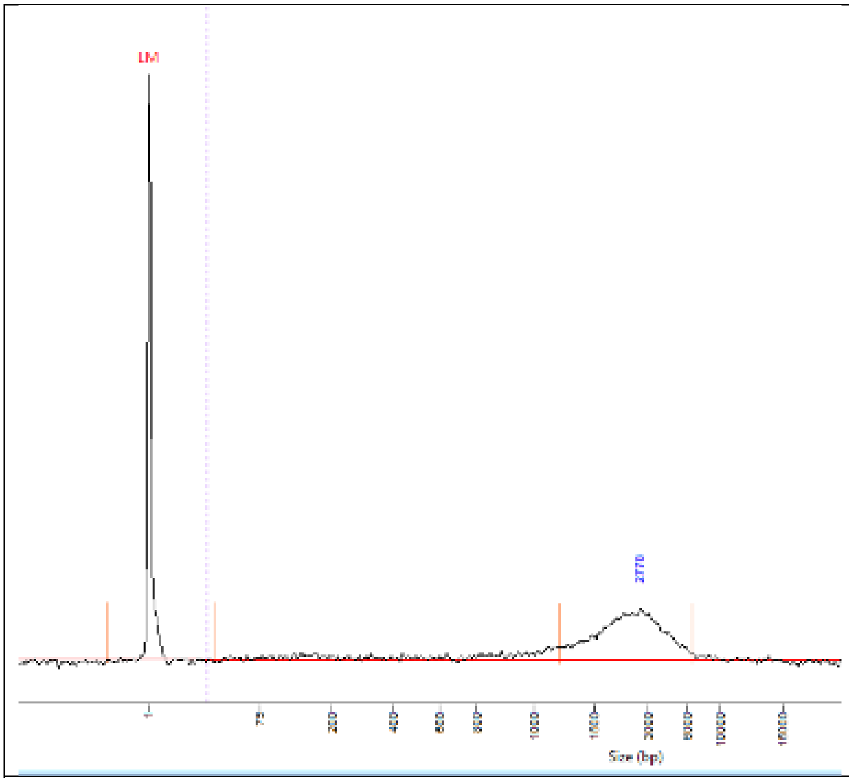

Supplementary Figure 53. Fragment Analyzer cDNA trace for sample 5356 (Control male). Peak at 2778 nucleotides.

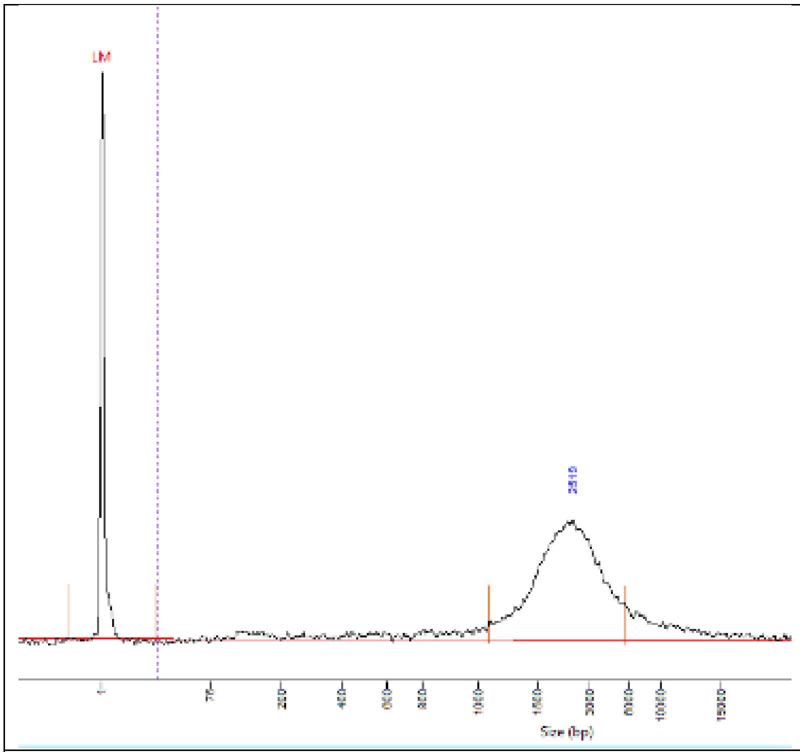

Supplementary Figure 54. Fragment Analyzer cDNA trace for sample 1304 (Control male). Peak at 2519 nucleotides.

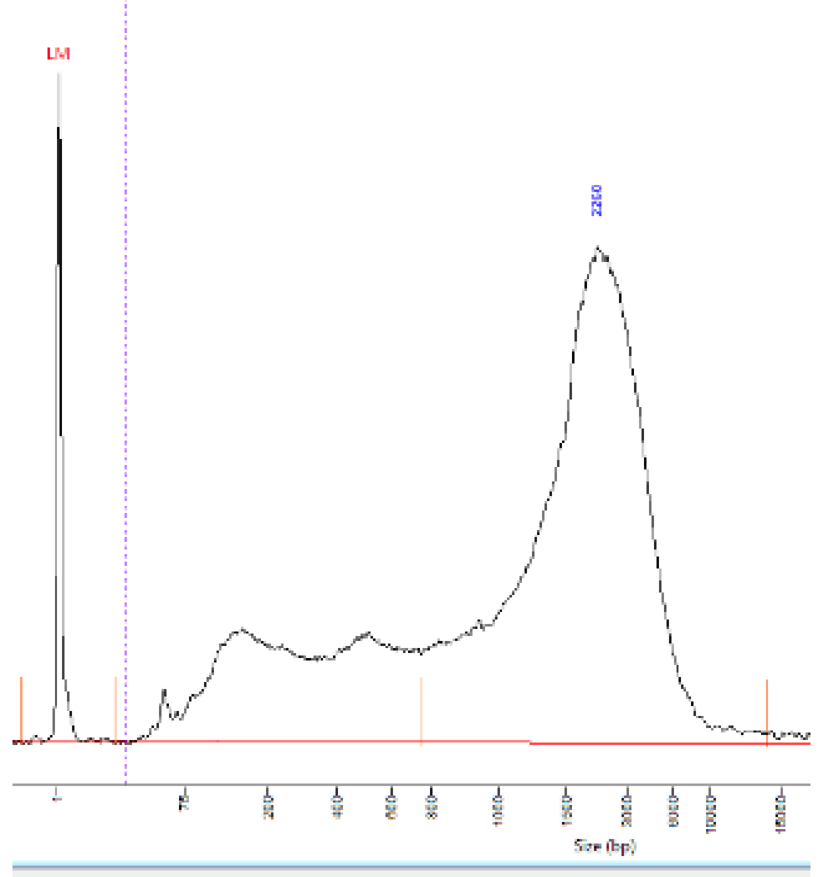

Supplementary Figure 55. Fragment Analyzer cDNA trace for sample 1271 (Control male). Peak at 2260 nucleotides.

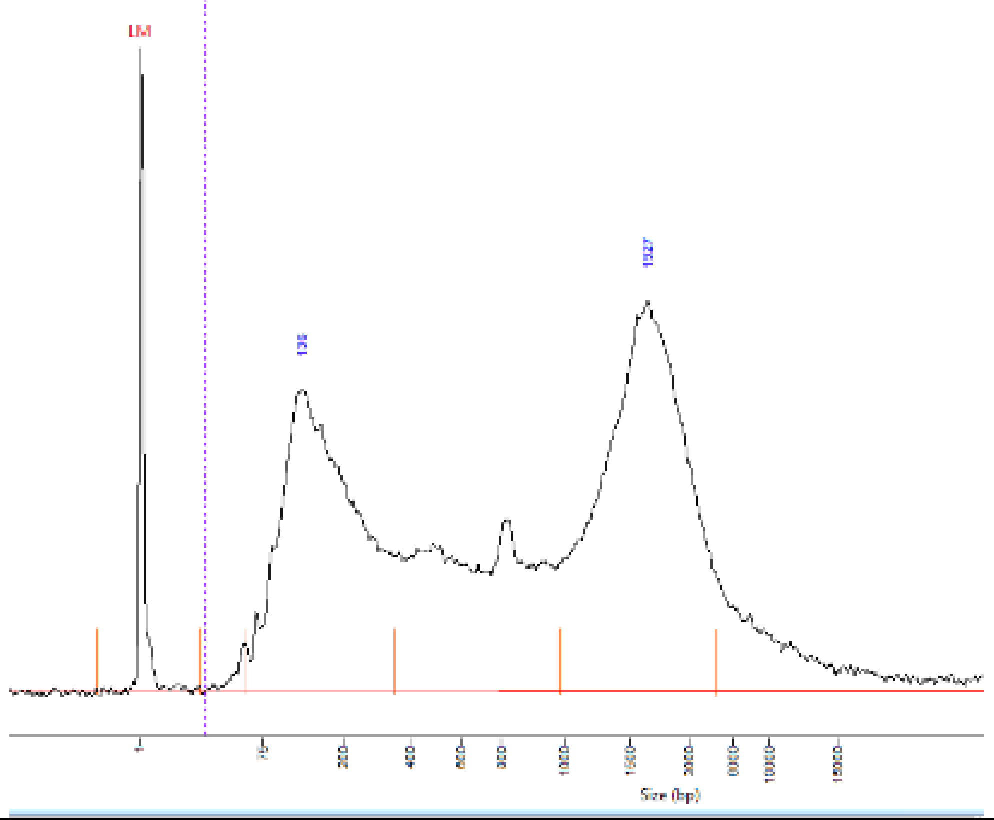

Supplementary Figure 56. Fragment Analyzer cDNA trace for sample 1186 (Alzheimer's disease female). Lower peak at 136 nucleotides, higher peak at 1927 nucleotides.

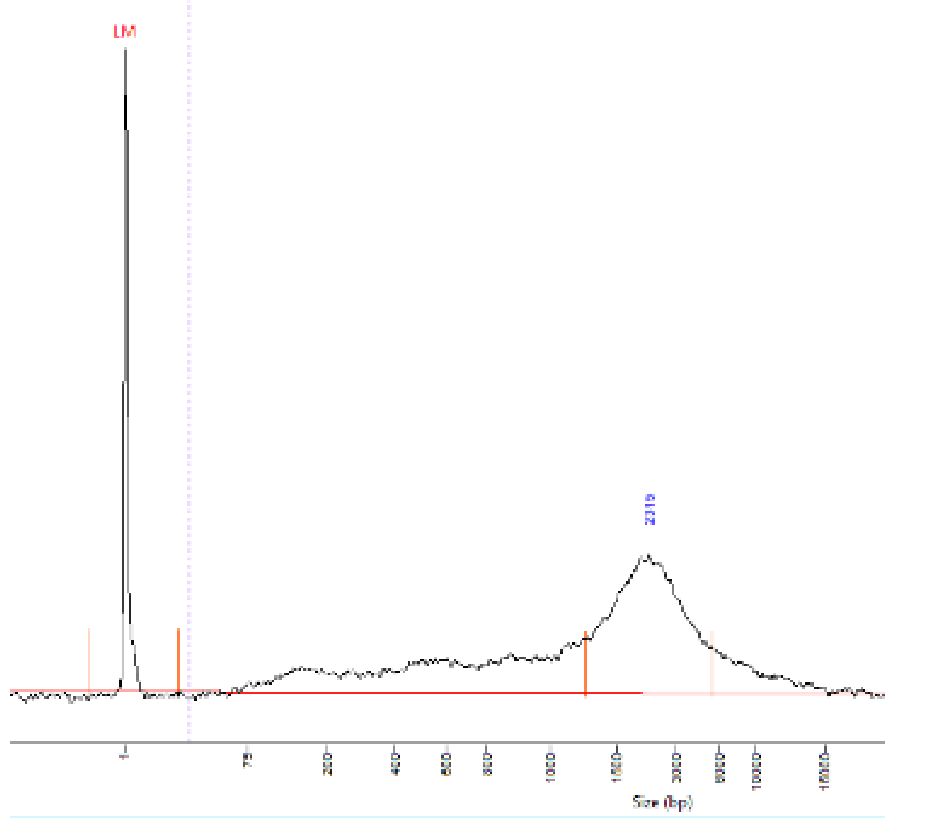

Supplementary Figure 57. Fragment Analyzer cDNA trace for sample 1218 (Alzheimer's disease female). Peak at 2315 nucleotides.

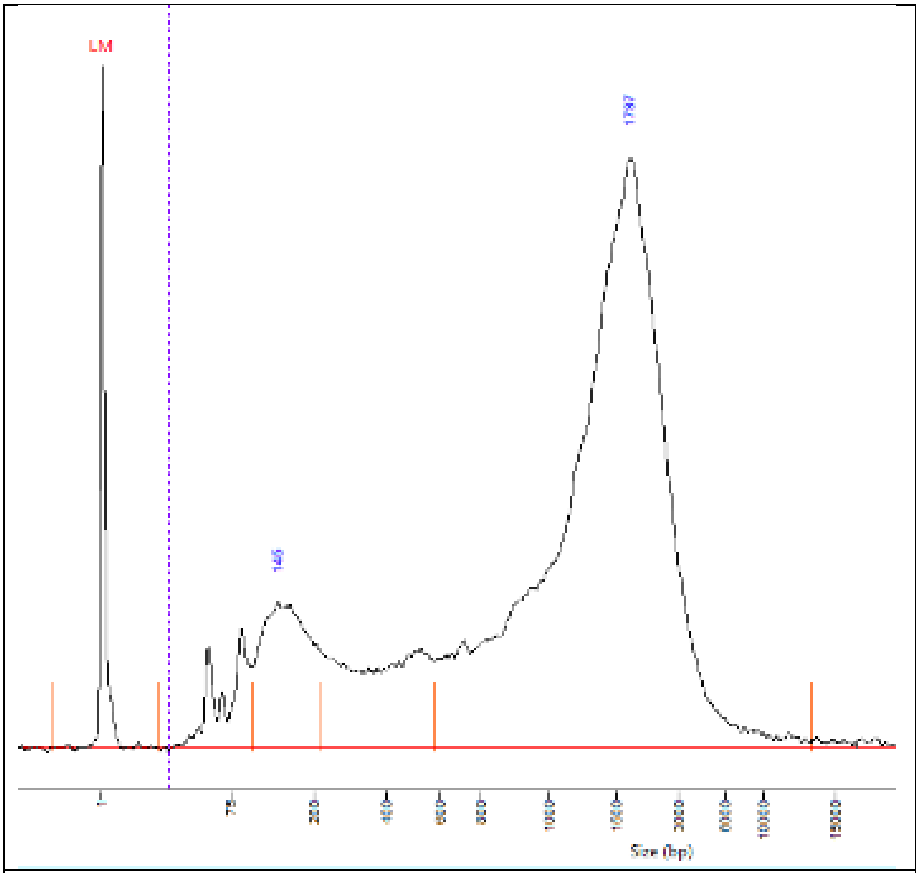

Supplementary Figure 58. Fragment Analyzer cDNA trace for sample 5295 (Alzheimer's disease female). Lower peak at 145 nucleotides, higher peak at 1797 nucleotides.

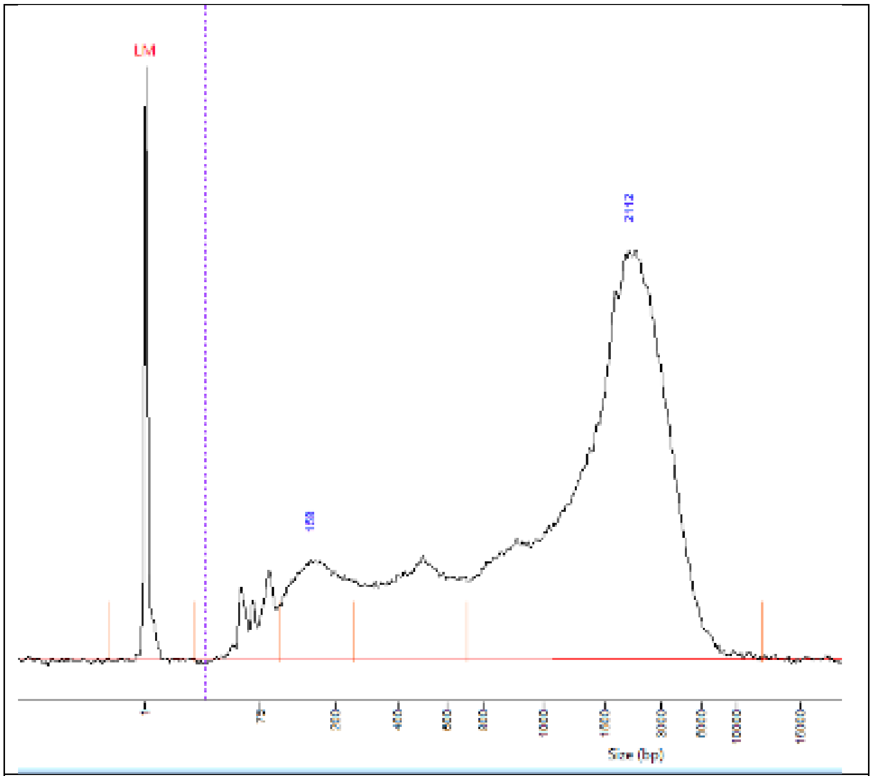

Supplementary Figure 59. Fragment Analyzer cDNA trace for sample 5292 (Alzheimer's disease female). Lower peak at 158 nucleotides, higher peak at 2112 nucleotides.

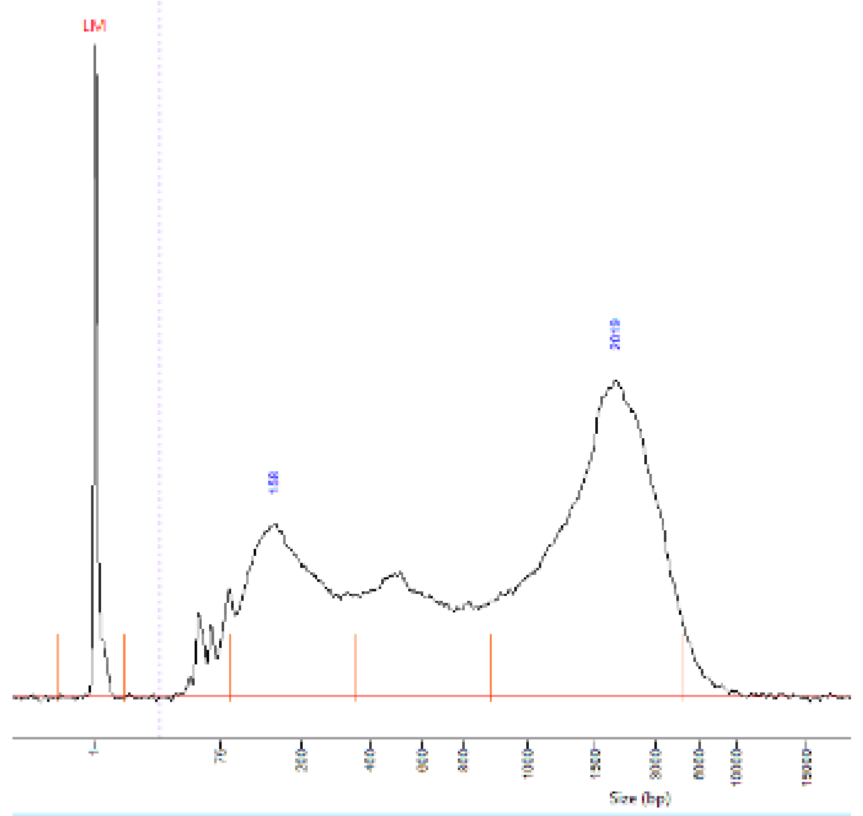

Supplementary Figure 60. Fragment Analyzer cDNA trace for sample 579 (Alzheimer’s disease female). Lower peak at 158 nucleotides, higher peak at 2019 nucleotides.

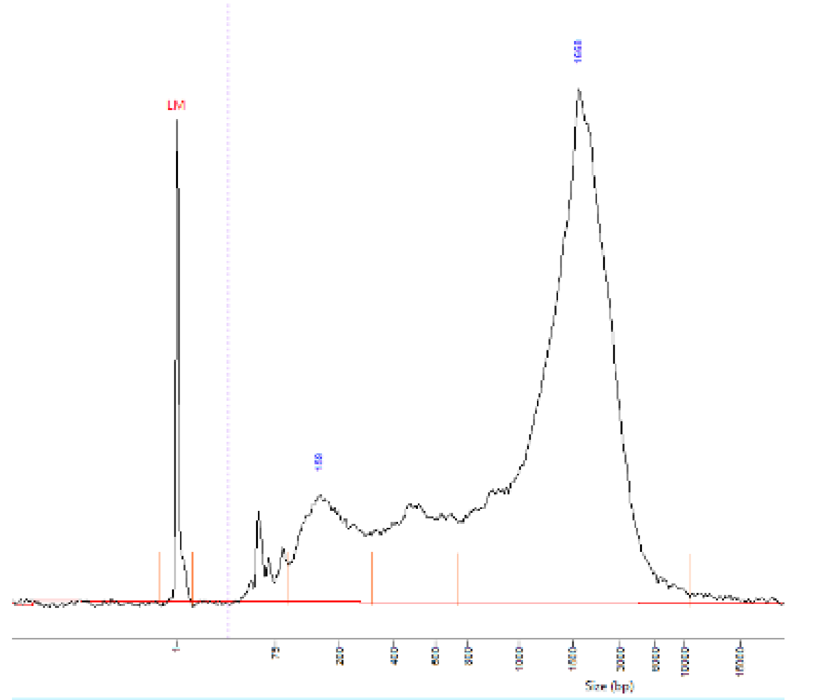

Supplementary Figure 61. Fragment Analyzer cDNA trace for sample 1291 (Alzheimer’s disease female). Lower peak at 159 nucleotides, higher peak at 1668 nucleotides.

LEXOGEN
The RNA Experts

SPLIT™

Pure RNA for all your experiments

RNA Extraction Kit
User Guide

Catalog Number:
008 (SPLIT RNA Extraction Kit)

008UG005V0320

FOR RESEARCH USE ONLY. NOT INTENDED FOR DIAGNOSTIC OR THERAPEUTIC USE.
INFORMATION IN THIS DOCUMENT IS SUBJECT TO CHANGE WITHOUT NOTICE.
Lexogen does not assume any responsibility for errors that may appear in this document.

PATENTS AND TRADEMARKS

SPLIT is a trademark of Lexogen. Lexogen is a registered trademark (EU, CH, US, CN, AU, NO, BR). Phase Lock Gel™ is a registered trademark of 5 Prime GmbH, RNA*later*™ and RNase*Zap*™ are registered trademarks of Ambion, Inc., RNasin® is a registered trademark of Promega Corporation. Bioanalyzer®, Fragment Analyzer™, and TapeStation® are trademarks of Agilent Technologies, Inc., DNA-ExitusPlus™ is a trademark of AppliChem GmbH, TRIzol® is a registered trademark of Molecular Research Center, Inc., MACS® is a registered trademark of Miltenyi Biotec B.V. All other brands and names contained in this user guide are the property of their respective owners.

Lexogen does not assume responsibility for violations or patent infringements that may occur with the use of its products.

LIABILITY AND LIMITED USE LABEL LICENSE: RESEARCH USE ONLY

This document is proprietary to Lexogen. The SPLIT RNA Extraction Kits are intended for use in research and development only. They need to be handled by qualified and experienced personnel to ensure safety and proper use. Lexogen does not assume liability for any damage caused by the improper use or the failure to read and explicitly follow this user guide. Furthermore, Lexogen does not assume warranty for merchantability or suitability of the product for a particular purpose.

The purchase of the product is subject to Lexogen general terms and conditions (www.lexogen.com/terms-and-conditions/) and does not convey the right to resell, distribute, further sublicense, repackage, or modify the product or any of its components. This document and its contents shall not be used or distributed for any other purpose and/or otherwise communicated, disclosed, or reproduced in any way without the prior written consent of Lexogen.

For information on purchasing additional rights or a license for use other than research, please contact Lexogen.

WARRANTY

Lexogen is committed to providing excellent products. Lexogen warrants that the product performs to the standards described in this user guide until the expiration date. Should this product fail to meet these standards due to any reason other than misuse, improper handling, or storage, Lexogen will replace the product free of charge or issue a credit for the purchase price. Lexogen does not provide any warranty if product components are replaced with substitutes.

Under no circumstances shall the liability of this warranty exceed the purchase price of this product.

LITERATURE CITATION

When describing a procedure for publication using this product, please refer to it as the SPLIT RNA Extraction Kit.

We reserve the right to change, alter, or modify any product without notice to enhance its performance.

CONTACT INFORMATION

Lexogen GmbH
Campus Vienna Biocenter 5
1030 Vienna, Austria
www.lexogen.com
E-mail: support@lexogen.com

Support
E-mail: support@lexogen.com
Tel. +43 (0) 1 3451212-41
Fax. +43 (0) 1 3451212-99

Table of Contents

1. Overview 4
2. Kit Components and Storage Conditions 6
3. User-Supplied Consumables and Equipment 8
4. Detailed Protocol 9
    4.1. Sample Homogenization 9
        4.1.1. Animal Tissue 9
        4.1.2. Plant Tissue 10
        4.1.3. Cultured Cells 11
        4.1.4. Fluid Samples 12
    4.2. Phenol / Chloroform Extraction 13
    4.3. Column-based Purification 14
        4.3.1. Column Loading of Total RNA 14
        4.3.2. Column Washing and Elution of RNA 15
5. Short Procedure 16
6. Appendix A: Sample Input and Extraction Efficiencies 17
7. Appendix B: RNA Quality Control 18
8. Appendix C: RNA Storage 20
9. Appendix D: Extraction of Large and Small RNA-enriched Fractions 21
    9.1. Column Loading of Large RNA 21
    9.2. Column Loading of Small RNA 22
    9.3. Short Procedure: Extraction of Large and Small RNA-enriched Fractions 23
10. Appendix E: Revision History 24

1. Overview

The SPLIT RNA Extraction Kit enables fast and highly efficient extraction of high quality, high purity RNA from a broad range of biological samples including cell culture, animal and plant tissue, and fluid samples. Thus the obtained RNA is ideal for seamless library preparation for Next Generation Sequencing and other demanding applications such as full-length reverse transcription, sample preparation for microarray analysis, or RT-qPCR. Furthermore, SPLIT recovers the complete RNA size ranges, including small RNAs (≥17 nt).

First, the sample is homogenized in a highly chaotropic isolation buffer which facilitates effortless and complete solubilization, and guarantees complete RNase inhibition.
Acidic buffer and acidic phenol are added to create a monophasic solution, a step that is essential for the efficient separation of genomic DNA into the organic phase. Chloroform is added, which creates a biphasic system, and phases are cleanly separated using Phase Lock Gel tubes. The use of these tubes mitigates the risk of contaminating the upper aqueous phase that contains RNA with the lower phenol phase that contains DNA and proteins.
The aqueous phase is mixed with isopropanol and the RNA is precipitated onto a silica column. Contaminants are efficiently cleared away by several washes and the eluted RNA is ready for any downstream application.

Please note that acidic phenol, chloroform, ethanol, and isopropanol have to be supplied by the user.

Protocols are given for RNA extraction from human cell culture, animal, and plant tissue, as well as fluid samples. Optionally, a modified protocol allows purification of small and large RNA-enriched fractions (see Appendix D, p.21).

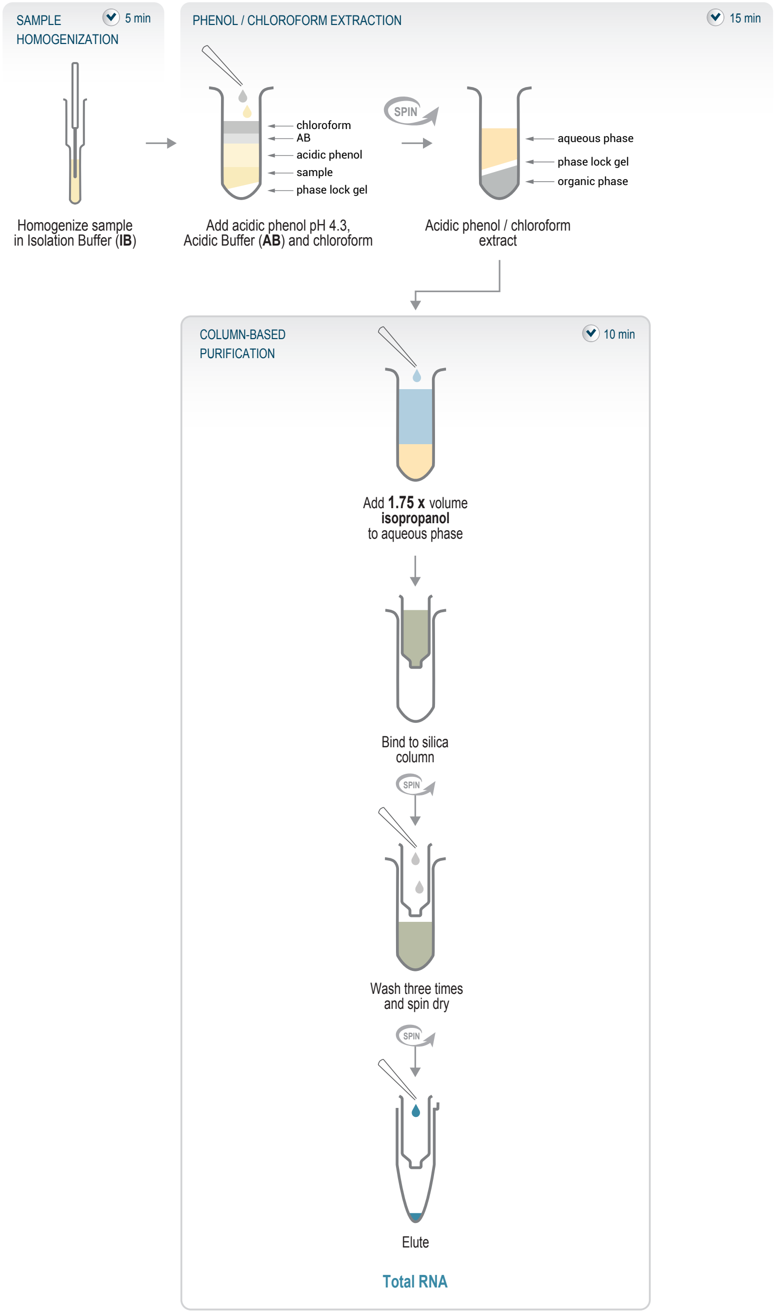

Figure 1. Schematic overview of the SPLIT workflow.

2. Kit Components and Storage Conditions

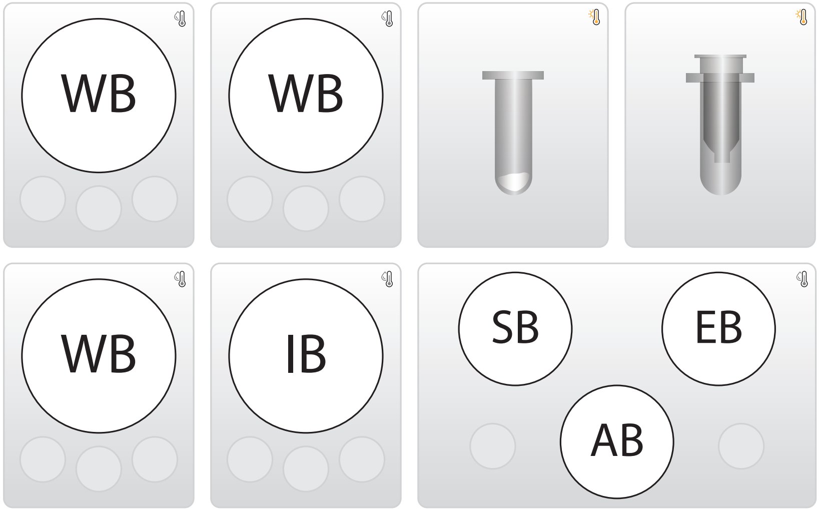

Figure 2. Location of kit components.

Kit Component	Label	Volume	Storage
**Isolation Buffer**	**IB**	21.1 ml *	+4 °C
**Acidic Buffer**	**AB**	7.9 ml *	+4 °C
**Wash Buffer**	**WB**	3 x 6.5 ml [1,*]	+4 °C
**Elution Buffer**	**EB**	2.6 ml [2,*]	+4 °C
**Storage Buffer**	**SB**	2.6 ml [2,*]	+4 °C
**Phase Lock Gel tubes**	**Phase Lock Gel tubes**	48	RT
**Purification columns**	**Purification columns**	48	RT
**Collection Tubes**	**Collection Tubes**	48	RT

*including ≥10 % surplus

[1] Excluding ethanol (to be added by the user - see bottle for volume to add).
[2] For each RNA fraction, either EB or SB is required.

Upon receiving the SPLIT (Cat. No. 008) kit, store it at +2 to +8 °C.
ATTENTION: Phase Lock Gel tubes must not be frozen.

The Isolation Buffer (**IB**) contains guanidine isothiocyanate, an irritant, which upon protocol completion is also present in flow-through and wash fractions. This chemical is harmful when in contact with the skin, inhaled, or ingested. Do not add bleach or acidic solutions directly to solutions or sample preparation waste that contains guanidine isothiocyanate, as reactive compounds and toxic gases are formed.

IB is to be used at +4 °C. All other components should be equilibrated to room temperature before use.

Check the contents of **IB**, **AB**, **WB**, and **SB** which may precipitate during shipping and storage. If a white precipitate is visible, incubate at 37 °C until buffer components dissolve completely.

Add 25 ml absolute ethanol to each of the three bottles with Wash Buffer (**WB**) concentrate and shake to combine.

3. User-Supplied Consumables and Equipment

Check to ensure that you have all of the necessary materials and equipment before beginning with the RNA extraction. All reagents, equipment, and labware must be free of nucleases and nucleic acid contamination.

ATTENTION: Before starting this protocol, please read the General Guidelines for Lexogen Kits, which are available online. These provide a detailed overview of RNA and kit component handling, as well as general RNA input requirements.

Reagents

Reagent	Volume	Comment
**Phenol solution pH 4.3**	19.2 ml	e.g., Sigma-Aldrich (P4682-100ML) or VWR (0981-400ML)
**Chloroform**	9.6 ml	
**Isopropanol**	~ 50.4 ml	2-Propanol
**Ethanol abs.**	3 x 25 ml	Added to **WB**

When working with the phenol solution and with chloroform, always work in a fume hood. Phenol is toxic and corrosive, and it should not come in contact with skin, eyes, or the respiratory tract and may cause chemical burns to the exposed area.

Equipment

- Fume hood for organic solvent handling.
- Refrigerated benchtop centrifuge (12,000 x g, rotor compatible with 1.5 ml and 2.0 ml tubes).
- Calibrated single-channel pipettes for handling 10 µl to 1,000 µl volumes.
- Vortex mixer.
- UV-spectrophotometer to quantify RNA.

Labware

- Suitable pipette tips (pipette tips with aerosol barriers recommended).
- 1.5 ml and 2.0 ml tubes with cap, low binding, certified ribonuclease-free.
- Benchtop cooler or ice pellets in ice box (for short-term storage of RNA).

Optional Equipment & Solutions

- Tissue grinder (hand-held homogenizer).
- Liquid nitrogen (for RNA extraction of plant tissue).
- Automated microfluidic electrophoresis station (Agilent Technologies 2100 Bioanalyzer).
- Agarose gels, dyes, and electrophoresis rig (for RNA quality control).
- DNA-ExitusPlus™ (AppliChem GmbH).
- RNaseZap.
- RNase inhibitor.

The complete set of materials, reagents, and labware for quality control is not listed.

4. Detailed Protocol

4.1. Sample Homogenization

4.1.1. Animal Tissue

Preparation

Tissue	Weigh and Reduce Tissue	Homogenization
**Animal tissue** – freshly harvested or frozen or thawed at +4 °C if stored in RNAlater	**Tweezers** – sterile<br>**Scalpel** – sterile<br>**Gauze pad** – sterile	**Isolation Buffer** (IB) - at +4 °C or on ice
**Fume hood** or laminar-flow cabinet	**Precision balance**	**Tissue grinder**

Homogenization

Tissue is homogenized in a highly chaotropic solution. This protocol is specific for hand-held tissue grinders (glass homogenizers with pestle) but can be easily adapted for other homogenization protocols. Optimally, the tissue should be stored at -20 °C in RNAlater (Ambion, Inc.). Tissue frozen without preservation must not be thawed before homogenization to maintain RNA integrity.

To prevent cross contamination, it is best to work in a fume hood or a laminar-flow cabinet that can be UV-irradiated.

1. Add 400 µl cold (+4 °C) Isolation Buffer (**IB**) into a glass tissue grinder.
   NOTE: Isolation Buffer (**IB**) contains detergent. Pipette carefully to avoid foaming.
2. Use sterile tweezers to transfer a tissue piece onto a fresh, sterile gauze pad. If RNAlater was used for conservation, dry the tissue by tapping onto the gauze pad.
3. Determine the weight of the tissue on a precision balance. The protocol is efficient for extraction of up to 10 µg of total RNA. See Appendix A, p.17 for details on input and extraction efficiency.
4. **OPTIONAL:** Hard to homogenize tissues such as tendons or cartilage can be reduced using a scalpel to facilitate solubilization in the next steps. Also, short incubation of tissue with Isolation Buffer (**IB**) prior to homogenization can help solubilization.
5. Using tweezers, transfer the tissue pieces quantitatively into the Isolation Buffer (**IB**) in the tissue grinder.

6 Homogenize the tissue by carefully moving the pestle up and down. Simultaneous rotation helps to dissolve also larger pieces. Do not pull out the pestle completely to avoid foaming. The tissue is usually homogenized within 2 - 3 minutes; avoid extended homogenization and warming up of Isolation Buffer (**IB**).

7 Continue immediately with the phenol / chloroform extraction at step 8 in 4.2., p.13

After use, clean the tissue grinder thoroughly with a detergent such as DNA-ExitusPlus™ (AppliChem GmbH), then rinse thoroughly with ultra-filtered water and finally with 75 % ethanol.

4.1.2. Plant Tissue

Preparation

Tissue	Weigh and Reduce Tissue	Homogenization
**Plant tissue –** freshly harvested or frozen at -80 °C or -20 °C in RNAlater or already ground and frozen in **IB** at -20 °C	**Tweezers** – sterile<br>**Scalpel** – sterile	**Liquid nitrogen**<br>**Isolation Buffer (IB)** - at +4 °C or on ice
**Fume hood** or laminar-flow cabinet	**Precision balance**	**Pestle and mortar**

Homogenization

Plant material is disrupted whilst frozen (i.e., in liquid nitrogen, or over dry ice) and homogenized in a highly chaotropic solution. Optimally, the plant material should be extracted immediately after harvesting. If storage of plant material is required, flash-freeze the sample in liquid nitrogen and store at -80 °C, or at -20 °C in RNAlater. Already ground plant tissue can also be stored in Isolation Buffer (**IB**) at -20 °C. To prevent cross contamination, it is best to work in a fume hood or a laminar-flow cabinet that can be UV-irradiated.

NOTE: Disruption of plant material can also be done using other devices such as ball mills and homogenization methods may need to be optimized for different types of input plant material (e.g., waxy, low water content tissues). For more information please contact support@lexogen.com.

1 Determine the weight of the tissue on a precision balance, working under sterile conditions (e.g., use sterile tweezers for transfer). The protocol is efficient for extraction of up to 10 µg of total RNA. See Appendix A, p.17 for details on input and extraction efficiency.

2 Quickly cut the plant tissue into small pieces using a scalpel and freeze in liquid nitrogen.

3 Grind the tissue using pestle and mortar. This can be done in liquid nitrogen, or by grinding the frozen tissue in a 1.5 ml or 2 ml tube over dry ice.

4 Allow any liquid nitrogen to evaporate.

5 Resuspend the tissue in 400 µl cold (+4 °C) Isolation Buffer (**IB**). Make sure to completely cover the tissue with **IB**.
NOTE: Isolation Buffer (**IB**) contains detergent. Pipette carefully to avoid foaming.

6 **OPTIONAL:** Further homogenize the sample by carefully moving the pestle up and down. Do not pull out the pestle completely to avoid foaming. Avoid extended homogenization and warming up of Isolation Buffer (**IB**).

7 Continue immediately with the phenol / chloroform extraction at step 8 in 4.2., p.13. **OPTIONAL:** ☞ Safe stopping point. Ground plant tissue in Isolation Buffer (**IB**) can be stored at -20 °C at this point.

After use, clean the pestle and mortar thoroughly with a detergent such as DNA-ExitusPlus™ (AppliChem GmbH), then rinse thoroughly with ultra-filtered water and finally with 75 % ethanol.

4.1.3. Cultured Cells

Preparation

Cells	Solubilization
**Cells** – freshly harvested, FACS / MACS sorted or frozen	**Isolation Buffer (IB)** – at +4 °C or on ice
**Fume hood** or laminar-flow cabinet	

Solubilization

Cells are solubilized in a highly chaotropic solution. Lyse the cells fully in the Isolation Buffer (**IB**) and proceed directly to RNA extraction. Cell lysates in **IB** can also be stored at -80 °C prior to extraction.
NOTE: Recommendations for freshly harvested and FACS / MACS sorted cells: spin down to pellet the cells and wash twice with Phosphate Buffered Saline (1x PBS). Remove PBS completely before adding IB. To prevent cross contamination, it is best to work in a fume hood or a laminar-flow cabinet that can be UV-irradiated.

ATTENTION: Do not store cells as cell pellets in IB without resuspending and lysing the cells beforehand. Cell lysis is required in order to maintain RNA integrity throughout storage in IB. Alternatively, snap freeze and store cell pellets after completely removing PBS and add IB when thawing to lyse cells before starting RNA extraction.

1 Harvest, pellet, and wash the cells. The protocol is suitable for extraction of e.g., 10^6 cells of a human suspension cell culture. SPLIT RNA extraction has also been successfully performed with 100 cells input.

2	Add 400 µl cold (+4 °C) Isolation Buffer (**IB**) to the cells.<br>**NOTE:** Isolation Buffer (**IB**) contains detergent. Pipette carefully to avoid foaming.
3	Lyse the cells by carefully pipetting up and down. Cells are usually lysed within 1 - 2 minutes.
4	Continue immediately with the phenol / chloroform extraction at step 8 in 4.2., p.13.

4.1.4. Fluid Samples

Preparation

Fluid samples	Solubilization
**e.g., plasma** – freshly harvested	**Isolation Buffer (IB)** – at +4 °C or on ice
**Centrifuge** – at +4 °C<br>**Fume hood** or laminar-flow cabinet	

Solubilization

The solubilization / homogenization step of the SPLIT protocol can be applied to a whole range of cells in fluids (aspirates, viral supernatants, plasma, urine etc.). Depending on the sample a homogenization step might be necessary or you can proceed directly to the phenol / chloroform extraction in 4.2., p.13, step 8. To prevent cross contamination, it is best to work in a fume hood or a laminar-flow cabinet that can be UV-irradiated.

NOTE: For sample types other than plasma, prior centrifugation may or may not be required. Use up to 200 µl of the liquid sample as input, then add 200 µl of Isolation Buffer (IB). For RNA extraction from whole blood, use up to 50 µl of a blood sample as an input.

ATTENTION: If the liquid sample volume is < 200 µl, add extra IB to bring the total volume of the sample / IB mix to 400 µl.

1	Centrifuge 300 - 400 µl of plasma at 12,000 x g for 5 minutes at 4 °C to pellet the cell debris.
2	Transfer 200 µl of the supernatant to a new tube. Take care to avoid carry-over of cell debris.
3	Add 200 µl Isolation Buffer (**IB**) and mix properly.<br>**NOTE:** Isolation Buffer (**IB**) contains detergent. Pipette carefully to avoid foaming.
4	Continue immediately with the phenol / chloroform extraction at step 8 in 4.2., p.13.

4.2. Phenol / Chloroform Extraction

Preparation

	For each sample	Temperature
**Phenol solution pH 4.3**[1]<br>**Acidic buffer (AB)**<br>**Chloroform**[1]	400 µl<br>150 µl<br>200 µl	+4 °C<br>RT<br>RT
**Phase Lock Gel tube**<br>**2 ml tube**	1<br>1	RT<br>RT
**Centrifuge** – at 18 °C<br>**Fume hood**<br>**Vortex mixer**		+18 °C

[1] **Caution:** When working with phenol or chloroform always use a fume hood and discard waste according to applicable Health and Safety regulations.

Phenol / Chloroform Extraction

Utilizing a highly specific phenol / chloroform extraction, RNA is partitioned into the upper, aqueous phase whereas DNA and proteins are partitioned into the lower, organic phase. The Phase Lock Gel matrix will act as a barrier in between the two phases.

8 For each sample, centrifuge one Phase Lock Gel tube for 1 minute at 12,000 x g at +18 °C. This collects the gel on the bottom of the tube. **ATTENTION:** Phase Lock Gel tubes should be equilibrated for 30 minutes at room temperature before use!

9 Transfer the homogenized sample in Isolation Buffer (**IB**) into a Phase Lock Gel tube.

10 Add 400 µl phenol solution pH 4.3 and mix by inverting the tube 5 times. **NOTE:** The phenol solution should be used at its storage temperature of +4 °C.

11 Add 150 µl Acidic Buffer (**AB**) and mix by pipetting.

12 Add 200 µl of chloroform.

13 Mix thoroughly by repeatedly inverting the tubes for 15 seconds (do not vortex!). **ATTENTION:** Thorough mixing is essential to disperse the chloroform efficiently and effectively separate all the phenol that will contain gDNA and protein into the organic and interphase.

14 Incubate for 2 minutes at room temperature.

15 Centrifuge for 2 minutes at 12,000 x g at +18 °C. **ATTENTION:** Temperatures below +18 °C can negatively influence phase separation. Repeat centrifugation at +18 °C if phase separation is incomplete.

16 Transfer the upper phase to a new 2 ml tube by decanting. **ATTENTION:** Do not transfer the upper phase by pipetting to avoid carry-over of the Phase Lock Gel.

17	For the purification of total RNA, proceed with **step 18 in 4.3.1.** For the purification of the large and small RNA-enriched fraction, proceed with **step 18 in Appendix D, p.21.**

4.3. Column-based Purification

Preparation

	Total RNA	Temperature
**Isopropanol**<br>**Wash Buffer (WB)**[1]<br>**Elution Buffer (EB) or**<br>**Storage Buffer (SB)**[2]	~1,050 µl<br>1,500 µl<br>50 µl	RT<br>RT<br>RT
**Purification column**<br>**Collection tube**<br>**1.5 ml tube**	1<br>1<br>1	RT<br>RT<br>RT
**Centrifuge**<br>**Vortex mixer**		+18 °C

[1] **Caution:** Discard waste containing guanidine isothiocyanate, phenol, and chloroform according to applicable Health and Safety regulations.

[2] See Appendix C, p.20 whether **EB** or **SB** should be used for elution.

NOTE: Repeat centrifugation or increase centrifugation time if sample did not pass filter completely.

4.3.1. Column Loading of Total RNA

The total RNA is precipitated onto a silica column by addition of 1.75x volume of isopropanol.

NOTE: To isolate the large and small RNA-enriched fractions (cutoff at ~ 150 nt), please see Appendix D, p.21.

18	Determine the volume of the aqueous phase, which may vary, depending on the sample volume and volume transfer efficiency during homogenization and extraction. Add isopropanol at 1.75x of this volume. Mix by vortexing for 10 seconds.<br>**EXAMPLE:** Add 1,050 µl isopropanol to 600 µl sample.
19	Place a purification column in a collection tube.
20	Apply a maximum of 800 µl of the mixture from step 18 (aqueous phase with isopropanol) to the column.
21	Centrifuge for 20 seconds at 12,000 x g at +18 °C and discard the contents of the collection tube.
22	Repeat steps 20 - 21 until the mixture is loaded completely then proceed to column washing and elution at step 23 in 4.3.2., p.15.

4.3.2. Column Washing and Elution of RNA

Column Washing

The RNA is further purified by washing on the column.

23	Apply 500 µl of Wash Buffer (**WB**) to the column and centrifuge for 20 seconds at 12,000 x g at +18 °C. Empty the collection tube. Repeat this step twice for a total of three washes.
24	Centrifuge for 1 minute at 12,000 x g at +18 °C.<br>**ATTENTION:** This step is essential to remove all traces of ethanol.
25	Discard the collection tube and place the purification column in new 1.5 ml tube.
26	Make sure that no ethanol traces are carried to the new tube.

Elution of RNA

The RNA is eluted into an elution or storage buffer.

27	Pre-warm the Elution Buffer (**EB**) or Storage Buffer (**SB**) for 5 minutes at 70 °C.
28	Add 10 - 50 µl of the pre-warmed Elution Buffer (**EB**) or Storage Buffer (**SB**) to the column and incubate for 1 minute at room temperature.
29	Centrifuge for 1 minute at 12,000 x g at +18 °C.
30	At this point the total RNA is purified and ready for quality control (Appendix B, p.18) and downstream applications.
31	**OPTIONAL:** Add RNase inhibitor (not included). See Appendix C, p.20 for RNA storage. Note that the RNase inhibitor might absorb at 230 nm, therefore use buffer with RNase inhibitor added as blank for OD measurements.

5. Short Procedure

Extraction of Total RNA

ATTENTION: All centrifugation steps are at 12,000 x g and +18 °C!

20 min Homogenization and Phenol / Chloroform Extraction

	**Homogenization**
☐	Homogenize sample in 400 µl **IB**.
	**Phenol / Chloroform Extraction**
☐	Centrifuge 1 Phase Lock Gel tube for 1 min.
☐	Transfer homogenate into a Phase Lock Gel tube.
☐	Add 400 µl phenol solution pH 4.3, mix by inverting the tube 5 times.
☐	Add 150 µl **AB**, mix by pipetting.
☐	Add 200 µl chloroform and mix by repeatedly inverting the tube for 15 sec.<br>**ATTENTION:** Do not vortex!
☐	Incubate for 2 min at RT.
☐	Centrifuge for 2 min (or longer if phase separation is incomplete).
☐	Decant the upper phase into a 2 ml tube.<br>**ATTENTION:** Do not transfer the upper phase by pipetting!

10 min Purification of Total RNA

	**Column Loading Total RNA**
☐	Measure volume of transferred upper phase.
☐	Add 1.75x vol. isopropanol to the upper phase.
☐	Mix by vortexing for 10 sec.
☐☐	Load max. 800 µl onto purification column in collection tube. Centrifuge for 20 sec and discard flow-through. Repeat until mixture is loaded completely.
	**Column Washing**
☐☐☐	Apply 500 µl **WB** and centrifuge for 20 sec. Empty collection tube. Repeat this step twice for a total of three washes.
☐	Centrifuge for 1 min to spin dry column.
	**Elution**
☐	Place purification column in a 1.5 ml tube.
☐	Pre-warm **EB** or **SB** for 5 min at 70 °C.
☐	Apply 10 - 50 µl of pre-warmed **EB** or **SB**, incubate for 1 min at RT.
☐	Centrifuge for 1 min.
☐	**OPTIONAL:** Add RNase inhibitor (not included).

NOTE: Repeat the centrifugation or increase centrifugation time if sample did not fully pass through the filter completely.

6. Appendix A: Sample Input and Extraction Efficiencies

If immediate RNA extraction is not possible, tissue samples can be either flash-frozen with liquid nitrogen and stored at -80 °C, or preserved in RNAlater (Ambion, Inc.) and stored at -20 °C or -80 °C. Tissues / cells frozen without RNAlater preservation must only be thawed during the homogenization step in cold Isolation Buffer (+4 °C) to keep RNases inactive.

RNA extraction efficiency for mouse liver is typically 4.0 - 4.5 µg total RNA / mg tissue (3.0 - 3.5 µg large RNA and 0.6 µg small RNA / mg tissue). The maximum binding capacity of the purification column is 10 µg RNA, which should not be exceeded for optimal results. For mouse liver tissue, this translates into an upper limit of 2.5 mg input per extraction. Other tissues have different RNA content, and the input might have to be adjusted accordingly.

The SPLIT RNA Extraction Kit has been used for isolation of RNA from different organisms including animal (e.g., mouse, human) and plant tissues (e.g., *A. thaliana*, *Picea abies*), insects (e.g., drosophila), cell lines (e.g., human), fluid samples (e.g., plasma), and others (jellyfish, fungi, bacteria). Please contact support@lexogen.com for information on protocol adaptations for other sample types.

7. Appendix B: RNA Quality Control

RNA Integrity

The integrity of an RNA sample can be assessed with a variety of methods (see Fig. 3). We recommend the use of a microfluidics assay such as the RNA6000 series for the 2100 Bioanalyzer (Agilent Technologies Inc.). However, RNA quality can also be assessed with denaturing agarose gel electrophoresis if such a device is not available. Most microfluidics platforms will carry out an automated peak analysis and generate a quality score (RIN or RQN) in addition to the 28S / 18S rRNA ratio. The quality of RNA extracted with the SPLIT RNA Extraction Kit almost exclusively depends on the extraction source: a RIN of 10 and a 28S / 18S rRNA ratio of 2.7 can be obtained from human cell culture homogenized according to 4.1.3. Extractions from tissue samples usually result in RNA with a RIN of 8.0 - 9.5.

Potential Contaminants

RNA samples should be free of salts, metal ions, and organic solvents, which can be carried over from the RNA extraction. Several sources of contamination can be detected with a UV-Vis spectrophotometer. An acceptably pure RNA sample should have an A260 / A280 ratio between 1.8 and 2.1. The A260 / A230 ratio should also be greater than 1.8. Several common contaminants including proteins, chaotropic salts and phenol absorb strongly between 220 and 230 nm and can often be identified as peaks in this region. Contamination with any of these generates a lower A260 / A230 ratio. Phenol also has an absorption maximum between 250 and 280 nm, which overlaps that of nucleic acid, so high 230 nm absorbance combined with a biphasic or broad peak between 250 and 280 nm may indicate contamination with phenol rather than chaotropic salts.

Genomic DNA Contamination

The SPLIT RNA Extraction Kit was designed for minimizing the genomic DNA (gDNA) content in the RNA sample. gDNA is indistinguishable from RNA on a spectrophotometer, and many of the dyes used in RNA microfluidics assays stain single-stranded nucleic acids much more intensely than double-stranded. Hence, low to moderate amounts of gDNA may not be readily visible with an RNA-specific microfluidics assay. We highly recommend examining all RNA samples on a denaturing agarose gel or using a fluorometric assay with DNA- and RNA-specific dyes to check samples for DNA contamination. On an agarose gel, gDNA can appear as either a dark mass which remains in the slot if relatively intact or as a high molecular weight smear if it has been sheared during extraction. For RNA-Seq, qRT-PCR, and other sensitive downstream applications, DNase I treatment in solution is recommended. For more information, please contact support@lexogen.com.

Typical Results

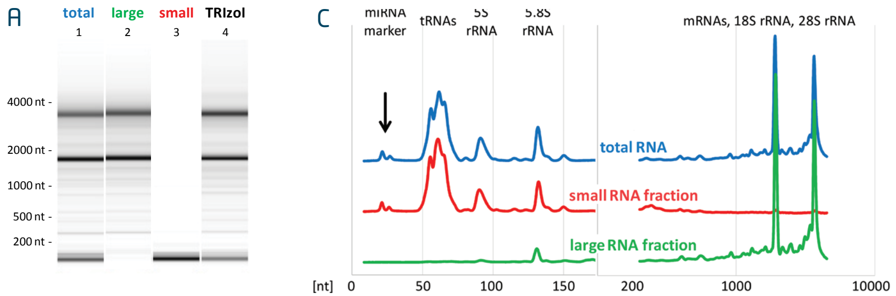

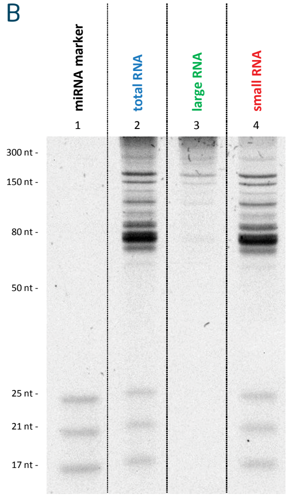

Figure 3. Analysis of SPLIT kit extracted RNA. A) Gel-like representation of Agilent Bioanalyzer traces. RNA from mouse liver stored in RNAlater was extracted either as total RNA (lane 1) or as large and small RNA-enriched fractions (lanes 2 and 3). In the split sample RNAs shorter than 150 nt are confined to the small RNA fraction. A control sample was extracted following a TRIzol protocol (lane 4). **B)** A miRNA marker was spiked into mouse liver homogenate, which was then extracted using the SPLIT kit. Analysis on a 15 % denaturing polyacrylamide gel demonstrates that small RNA down to at least 17 nt is efficiently recovered in the total RNA sample and in the small RNA -enriched fraction. The theoretical maximum spike-in RNA recovery amount was loaded in lane 1. **C)** Bioanalyzer evaluation of miRNA-spiked samples on a small RNA chip (10 - 200 nt, linear scale) and on an RNA 6000 pico chip (200 - 500 nt, log scale). The traces from the two chips are shown alongside for illustrative purposes, the Y-axes do not correspond quantitatively.

8. Appendix C: RNA Storage

After extraction, RNA can be stored in Elution Buffer (**EB**, 10 mM Tris-HCl pH 7.0) at -20 °C or -80 °C. This minimal buffer stabilizes the pH without any other components that might interfere with downstream applications. When eluting in **EB** we highly recommend the addition of RNase inhibitors to block any accidentally introduced RNases.
The Storage Buffer (**SB**, 10 mM Tris-HCl pH 7.0, 10 mM DTT, and 0.1 mM EDTA) supplied with these kits can be used for intermediate storage of the RNA at -20 °C or -80 °C. DTT (antioxidant) and EDTA (chelating agent) both minimize the threat of RNA degradation, especially at non-freezing conditions. For long-term storage, we recommend keeping aliquots of the RNA as sodium acetate / ethanol precipitate at -80 °C to avoid accidental RNase contamination as well as RNA degradation due to freeze-thaw cycles.
We suggest checking the RNA quality after extended periods of storage for changes in integrity and quantity e.g., on a microfluidics system.

9. Appendix D: Extraction of Large and Small RNA-enriched Fractions

RNA can be split into large and small RNA-enriched fractions following the optional column loading protocols below.

ATTENTION: The SPLIT RNA Extraction Kit contains reagents for the isolation of total RNA or the large RNA-enriched fraction from 48 samples, or small and large RNA-enriched fractions from 24 samples.

9.1. Column Loading of Large RNA

The large RNA-enriched fraction is precipitated onto a silica column by adding 0.33x volume of isopropanol. The small RNA-enriched fraction will be in the flow-through and can be further purified (see the protocol below).

18	Determine the volume of the aqueous phase, which may vary, depending on the tissue volume and volume transfer efficiency during homogenization and extraction. Add isopropanol at 0.33x of this volume (e.g., 200 µl isopropanol to 600 µl sample). Mix by vortexing for 10 seconds.<br>**ATTENTION:** For best reproducibility of the size cutoff it is essential to quantify the volume of the aqueous phase exactly.
19	Place a purification column in a collection tube.
20	Apply a maximum of 800 µl of the mixture from step 18 (aqueous phase with isopropanol) to the column.
21	Centrifuge for 20 seconds at 12,000 x g at +18 °C.<br>**ATTENTION:** To isolate the small RNA fraction, pipette the flow-through into a 2 ml tube.
22	Repeat steps 20 - 21 until the mixture is loaded completely and then proceed to column washing and elution at step 23 in 4.3.2., p.15.<br>**NOTE:** If only the small RNA-enriched fraction is of interest, discard the spin-column containing the large RNA and collect the flow-through into the 2 ml tube. Continue to Column Loading of Small RNA at step 18 in 9.2. (below). Else discard the flow-through.

9.2. Column Loading of Small RNA

The flow-through obtained in steps 21 - 22 of 9.1. (Column Loading of Large RNA) contains the small RNA fraction and is recovered by precipitation onto a new purification column with the addition of 1x volume of isopropanol.

18	Determine the total volume of the flow-through in the 2 ml tube (from step 22, 9.1.) and add the same volume of isopropanol. Mix by vortexing for 10 seconds. **EXAMPLE:** 800 µl isopropanol to 800 µl flow-through.
19	Place a purification column in a collection tube.
20	Apply a maximum of 800 µl of the mixture from step 18 (aqueous phase with isopropanol) to the column.
21	Centrifuge for 20 seconds at 12,000 x g at +18 °C and discard the content of the collection tube.
22	Repeat steps 20 - 21 until the mixture is loaded completely then proceed to column washing and elution at step 23 in 4.3.2., p.15.

9.3. Short Procedure: Extraction of Large and Small RNA-enriched Fractions

ATTENTION: All centrifugation steps are at 12,000 x g and +18 °C!

20 min Homogenization and Phenol / Chloroform Extraction

Homogenization

- ☐ Homogenize sample in 400 µl **IB**.

Phenol / Chloroform Extraction

- ☐ Centrifuge 1 Phase Lock Gel tube for 1 min.
- ☐ Transfer homogenate into a Phase Lock Gel tube.
- ☐ Add 400 µl phenol solution pH 4.3, mix by inverting the tube 5 times.
- ☐ Add 150 µl **AB**, mix by pipetting.
- ☐ Add 200 µl chloroform and mix by repeatedly inverting the tube for 15 sec.

ATTENTION: Do not vortex!

- ☐ Incubate for 2 min at RT.
- ☐ Centrifuge for 2 min.
- ☐ Decant the upper phase into a 2 ml tube.

ATTENTION: Do not transfer the upper phase by pipetting!

15 min Purification of Large / Small RNA-enriched Fraction(s)

Column Loading Large RNA-enriched Fraction

- ☐ Measure volume of transferred upper phase.
- ☐ Add 0.33x vol. isopropanol to the upper phase.
- ☐ Mix by vortexing for 10 sec.
- ☐☐ Load max. 800 µl onto purification column in collection tube. Centrifuge for 20 sec. Repeat until mixture is loaded completely.

ATTENTION: Keep flow-through and transfer into a 2 ml tube if small RNA extraction is desired. If only small RNA extraction is desired, discard the spin column.

Column Loading Small RNA-enriched Fraction

- ☐ Measure flow-through volume in 2 ml tube and add 1x vol. isopropanol. Mix by vortexing for 10 sec.
- ☐☐ Load max. 800 µl onto new purification column in a collection tube. Centrifuge for 20 sec. Discard flow-through and repeat until mixture is loaded completely.

Column Washing

- ☐☐☐ Apply 500 µl **WB** to each column and centrifuge for 20 sec. Empty collection tube(s). Repeat this step twice for a total of three washes.
- ☐ Centrifuge for 1 min to spin dry column(s).

Elution

- ☐ Place purification column(s) in a 1.5 ml tube.
- ☐ Pre-warm **EB** or **SB** for 5 min at 70 °C.
- ☐ Apply 10 - 50 µl of pre-warmed **EB** or **SB**, incubate for 1 min at RT.
- ☐ Centrifuge for 1 min.
- ☐ **OPTIONAL:** Add RNase inhibitor (not included).

NOTE: Repeat the centrifugation or increase centrifugation time if sample did not pass through the filter completely.

10. Appendix E: Revision History

Publication No. / Revision Date	Change	Page
008UG005V0320<br>Oct. 10, 2022	Legal terms and conditions statements updated.	2
	Schematic overview updated.	5
	Kit components figure updated.	6
	Guidelines replaced with the hyperlink to General Guidelines for Lexogen Kit Use.	8
	Protocol for Extraction of Large and Small RNA-enriched Fractions moved to Appendix.	21 - 23
	SPLIT for Blood Cat. No. 099 removed from the User Guide.	14
008UG005V0100<br>Aug. 19, 2013	Initial Release.	

The RNA Experts

Associated Products:

015 (QuantSeq 3' mRNA-Seq Library Prep Kit for Illumina (FWD))
016 (QuantSeq 3' mRNA-Seq Library Prep Kit for Illumina (REV) with Custom Sequencing Primer)
025, 050, 051, 141 (SIRVs Spike-in RNA Variant Control Mixes)
052 (Small RNA-Seq Library Prep Kit for Illumina)
070 (RS-Globin Block, *homo sapiens*)
095 (CORALL Total RNA-Seq Library Prep Kit)
113 - 115, 129 - 131 (QuantSeq 3' mRNA-Seq Library Prep Kit FWD with UDIs)
117 - 119, 132 - 134 (CORALL Total RNA-Seq Library Prep Kit with UDIs)
128 (TraPR Small RNA Isolation Kit)
144 (RiboCop rRNA Depletion Kit for Human/Mouse/Rat (HMR) V2)
145 (RiboCop rRNA Depletion Kit for Human/Mouse/Rat Plus Globin (HMR+Globin))
171 - 176 (CORALL RNA-Seq V2 Library Prep Kit with UDIs)

SPLIT RNA Extraction Kit · User Guide

Lexogen GmbH
Campus Vienna Biocenter 5
1030 Vienna, Austria
Telephone: +43 (0) 1 345 1212-41
Fax: +43 (0) 1 345 1212-99
E-mail: support@lexogen.com
© Lexogen GmbH, 2022

Lexogen, Inc.
51 Autumn Pond Park
Greenland, NH 03840, USA
Telephone: +1-603-431-4300
Fax: +1-603-431-4333
www.lexogen.com
