# Supplementary material for: Mapping medically relevant RNA isoform diversity in the aged human frontal cortex with deep long-read RNA-seq
Source: Nat Biotechnol. 2024 May 22;43(4):635–46. doi: 10.1038/s41587-024-02245-9 (PMC11863200; doi:10.1038/s41587-024-02245-9)
Supplement: Supplementary file 2 — Reporting Summary [file 41587_2024_2245_MOESM2_ESM.pdf]

nature portfolio

Corresponding author(s): Mark T. W. Ebbert

Last updated by author(s): Feb 5, 2024

Reporting Summary

Nature Portfolio wishes to improve the reproducibility of the work that we publish. This form provides structure for consistency and transparency in reporting. For further information on Nature Portfolio policies, see our Editorial Policies and the Editorial Policy Checklist.

Statistics

For all statistical analyses, confirm that the following items are present in the figure legend, table legend, main text, or Methods section.

n/a	Confirmed	
☐	☒	The exact sample size (*n*) for each experimental group/condition, given as a discrete number and unit of measurement
☐	☒	A statement on whether measurements were taken from distinct samples or whether the same sample was measured repeatedly
☐	☒	The statistical test(s) used AND whether they are one- or two-sided<br>*Only common tests should be described solely by name; describe more complex techniques in the Methods section.*
☒	☐	A description of all covariates tested
☐	☒	A description of any assumptions or corrections, such as tests of normality and adjustment for multiple comparisons
☐	☒	A full description of the statistical parameters including central tendency (e.g. means) or other basic estimates (e.g. regression coefficient) AND variation (e.g. standard deviation) or associated estimates of uncertainty (e.g. confidence intervals)
☐	☒	For null hypothesis testing, the test statistic (e.g. F, t, r) with confidence intervals, effect sizes, degrees of freedom and P value noted<br>*Give P values as exact values whenever suitable.*
☒	☐	For Bayesian analysis, information on the choice of priors and Markov chain Monte Carlo settings
☒	☐	For hierarchical and complex designs, identification of the appropriate level for tests and full reporting of outcomes
☐	☒	Estimates of effect sizes (e.g. Cohen's *d*, Pearson's *r*), indicating how they were calculated

Our web collection on statistics for biologists contains articles on many of the points above.

Software and code

Policy information about availability of computer code

Data collection

MinKNOW version 23.04.5

Data analysis

Guppy GPU basecaller version 6.3.9 ; pychopper version 2.7.2 ; minimap2 version 2.22-rll0l ; samtools version 1.6

PycoQC version 2.5.2 ; bambu version 3.0.5 ; R version 4.2.2 (all data analysis besides website development)

Online meme suite tool version 5.5.3 (https://meme-suite.org/meme/tools/meme) ; gffcompare version 0.11.2

Python version 3.10.8 ; DESeq2 version 1.38.3 ; tidyverse version 1.3.2 ; EnhancedVolcano 1.18.0 ; gffread version 0.12.7

ggtranscript 0.99.3 ; numpy version 1.24.1 ; pandas version 1.5.2 ; regex version 2022.10.31 ; plotly version 5.11.0

trim galore version 0.6.6 ; matplotlib version 3.6.2 ; seaborn version 0.12.2 ; matplotlib_venn version 0.11.7 ; wordcloud version 1.8.2.2

notebook version 6.5.2 Online NCBI primer design tool <no version provided> (https://www.ncbi.nlm.nih.gov/tools/primer-blast/)

pypGATK67 version 0.0.23 ; FragPipe version 20.0 ; nextflow version 23.04.1.5866 ; singularity version 3.8.0-1.el8 ;

STAR aligner version 2.7.l0b ; salmon version 0.13.1 ; Rshiny version 1.7.4 ; R version 4.2.1 (only for website development) ;

Online protein-protein NCBI blast tool <no version provided> (blastp - https://blast.ncbi.nlm.nih.gov/Blast.cgi?PAGE=Proteins)

All code used in the manuscript is publicly available at: https://github.com/UK-SBCoA-EbbertLab/brain_cDNA_discovery

For manuscripts utilizing custom algorithms or software that are central to the research but not yet described in published literature, software must be made available to editors and reviewers. We strongly encourage code deposition in a community repository (e.g. GitHub). See the Nature Portfolio guidelines for submitting code & software for further information.

Data

Policy information about availability of data

All manuscripts must include a data availability statement. This statement should provide the following information, where applicable:

- Accession codes, unique identifiers, or web links for publicly available datasets
- A description of any restrictions on data availability
- For clinical datasets or third party data, please ensure that the statement adheres to our policy

- Raw long-read RNAseq data generated and utilized in this manuscript are publicly available in Synapse: https://www.synapse.org/#!Synapse:syn52047893.
- Raw long-read RNAseq data generated and utilized in this manuscript are also publicly available in NIH SRA (accession number: SRP456327): https://trace.ncbi.nlm.nih.gov/Traces/?view=study&acc=SRP456327
- Output from long-read RNAseq and proteomics pipelines, reference files, and annotations are publicly available here: https://doi.org/10.5281/zenodo.8180677-
- Long-read RNAseq results from this article can be easily visualized through this web application: https://ebbertlab.com/brain_rna_isoform_seq.html
- Raw cell-line deep proteomics data used utilized in this article are publicly available here: https://proteomecentral.proteomexchange.org/cgi/GetDataset?ID=PXD024364
- Raw brain proteomics data from round 2 of the ROSMAP TMT study are publicly available here: https://www.synapse.org/#!Synapse:syn17015098
- GTEx long-read RNAseq data used for validation of our study results is available here: https://anvil.terra.bio/#workspaces/anvil-datastorage/AnVIL_GTEx_V9_hg38
- ROSMAP short-read RNAseq data used for validation of our study results is available here: https://www.synapse.org/#!Synapse:syn21589959
- CHM13 reference genome sequence can be found here: https://s3-us-west-2.amazonaws.com/human-pangenomics/T2T/CHM13/assemblies/analysis_set/chm13v2.0.fa.gz
- CHM13 reference GFF3 annotation can be found here: https://s3-us-west-2.amazonaws.com/human-pangenomics/T2T/CHM13/assemblies/annotation/chm13.draft_v2.0.gene_annotation.gff3
- The transcript annotation from Glinos et al.19 was retrieved from: https://storage.googleapis.com/gtex_analysis_v9/long_read_data/flair_filter_transcripts.gtf.gz.
- The transcript annotation from Leung et al.20 was retrieved from: https://zenodo.org/record/7611814/preview/Cupcake_collapse.zip#tree_item12/HumanCTX.collapsed.gff.

Research involving human participants, their data, or biological material

Policy information about studies with human participants or human data. See also policy information about sex, gender (identity/presentation), and sexual orientation and race, ethnicity and racism.

Reporting on sex and gender	Our sample consisted of six females (sex) and six males (sex). Out of the six females, three were pathologically confirmed Alzheimer's diseases cases and 3 were controls. Out of the six males, three were pathologically confirmed Alzheimer's disease cases and three were controls.
Reporting on race, ethnicity, or other socially relevant groupings	Six Alzheimer's disease cases and six controls. The samples were aged, with ages ranging between 71 and 94 years of age. All samples came from Caucasian individuals.
Population characteristics	Six Alzheimer's disease cases and six controls. The samples were aged, with ages ranging between 71 and 94 years of age. All samples came from Caucasian individuals.
Recruitment	Recruitment efforts (beginning fall 1989) involved contacting potential volunteers from a registry of over 4,500 community residents over 60 years of age, who had earlier indicated a willingness to participate in research, following a mailing to all registered voters in Fayette County, Kentucky. Potential participants received an introductory letter summarizing the importance of autopsy, followed by a visit with a center staff member to provide information about the project. Other volunteers came to the program following articles in the local press and broadcast news media.
Ethics oversight	University of Kentucky Institutional Review Board (IRB).

Note that full information on the approval of the study protocol must also be provided in the manuscript.

Field-specific reporting

Please select the one below that is the best fit for your research. If you are not sure, read the appropriate sections before making your selection.

☒ Life sciences ☐ Behavioural & social sciences ☐ Ecological, evolutionary & environmental sciences

For a reference copy of the document with all sections, see nature.com/documents/nr-reporting-summary-flat.pdf

Life sciences study design

All studies must disclose on these points even when the disclosure is negative.

Sample size

12 samples - Sample size calculation is unnecessary in exploratory studies aimed at observing trends rather than conclusive findings. Sample size chosen based on practical concerns and estimation of enough preliminary data to observe trends and inform future larger studies.

Data exclusions

None

Replication

New RNA isoforms from known gene bodies:

We attempted to validate 18 new high-confidence isoforms through PCR and gel electrophoresis and successfully validated nine of them (Fig. 2g, Supplementary Fig. 5-26, Supplementary Table 4). We then attempted to validate the nine RNA isoform that failed via standard PCR (no visible band on gel) using RT-qPCR—a more sensitive method compared to PCR and gel electrophoresis—and successfully validated seven of them (Supplementary Table 5). MIQE guidelines by Bustin et al.25 suggests Ct < 40 as a cutoff for RT-qPCR validation, but we used a more stringent cutoff of Ct < 35 to be more conservative. Out of the 16 transcripts that were successfully validated through PCR and gel electrophoresis or RT-qPCR, 11 are unique to this study.

We attempted to validate our new high-confidence transcripts from known genes using publicly available long-read RNAseq data from five GTEx18 brain (Brodmann area 9) samples and short-read RNAseq data from 251 ROSMAP21 brain samples (Brodmann area 9/46). We observed that 98.8% of the new high-confidence transcripts from known gene bodies had at least one uniquely mapped read in either GTEx or ROSMAP data and 69.6% had at least 100 uniquely mapped reads in either dataset (Extended Data Fig. 7, Supplementary Table 8). Importantly, while a single unique read within short-read data may seem like a soft threshold, only a small percentage of short reads are unique to a single isoform for genes expressing multiple isoforms.

New RNA isoforms from new gene bodies:

We attempted to validate 12 new high-confidence RNA isoforms from new gene bodies through PCR and gel electrophoresis and successfully validated 11 of them (Fig. 4h, Supplementary Fig. 5-26, Supplementary Table 4). The one RNA isoform that failed to validate via standard PCR (no clear band on gel) successfully validated through RT-qPCR (mean Ct = 23.2; Supplementary Table 5). MIQE guidelines by Bustin et al.25 suggests Ct < 40 as a cutoff for RT-qPCR validation, but we used a more stringent cutoff of CT < 35 to be more conservative. All 12 new RNA isoforms from new gene bodies that were validated through PCR and gel electrophoresis and RT-qPCR are unique to this study.

We attempted to validate our new high-confidence transcripts using publicly available long-read RNAseq data from five GTEx18 brain samples (Brodmann area 9) and short-read RNAseq data from 251 ROSMAP21 brain samples (Brodmann area 9/46). Over 94.4% of the new high-confidence transcripts from new gene bodies had at least one uniquely mapped read in either GTEx or ROSMAP data and over 44.2% had at least 100 uniquely mapped reads in either dataset (Extended Data Fig. 7, Supplementary Table 8).

New RNA isoforms from spliced mitochondrial RNA isoforms:

We attempted to validate three new high-confidence mitochondrially encoded isoforms through PCR and successfully validated two of them (Supplementary Fig. 25,26). It was not possible to design specific primers for the other two new high-confidence mitochondrial isoforms because of low sequence complexity or overlap with other lowly expressed (low-confidence) mitochondrial RNA isoforms found in our data; thus, we did not attempt to validate them using PCR. While there are more advanced and traditional methods to validate these experimentally, we feel that direct validation of the other spliced mitochondrial isoforms we discovered via PCR, combined with the exceptional and thorough work by Herai et al.56 demonstrates that at least some, if not all of those we observed are real, which is our primary objective. Notably, however, we were able to validate all five high-confidence spliced mitochondrial transcripts in the data from Glinos et al.18, as each had at least 100 uniquely aligned counts across each of the 5 GTEx brain samples (Extended Data Fig. 7).

Randomization

Not relevant (observational study). Controlling for covariates is unnecessary in exploratory proof of concept studies aimed at observing trends rather than conclusive findings.

Blinding

None, blinding is unnecessary due to the objective, automated nature of RNA sequencing analysis, minimizing subjective bias.

Reporting for specific materials, systems and methods

We require information from authors about some types of materials, experimental systems and methods used in many studies. Here, indicate whether each material, system or method listed is relevant to your study. If you are not sure if a list item applies to your research, read the appropriate section before selecting a response.

Materials & experimental systems

n/a	Involved in the study
☒	☐ Antibodies
☒	☐ Eukaryotic cell lines
☒	☐ Palaeontology and archaeology
☒	☐ Animals and other organisms
☒	☐ Clinical data
☒	☐ Dual use research of concern
☒	☐ Plants

Methods

n/a	Involved in the study
☒	☐ ChIP-seq
☒	☐ Flow cytometry
☒	☐ MRI-based neuroimaging

Plants

Seed stocks

Does not apply.

Novel plant genotypes

Does not apply.

Authentication

Does not apply.
